# PDK4-dependent hypercatabolism and lactate production of senescent cells promotes cancer malignancy

**DOI:** 10.1038/s42255-023-00912-w

**Published:** 2023-10-30

**Authors:** Xuefeng Dou, Qiang Fu, Qilai Long, Shuning Liu, Yejun Zou, Da Fu, Qixia Xu, Zhirui Jiang, Xiaohui Ren, Guilong Zhang, Xiaoling Wei, Qingfeng Li, Judith Campisi, Yuzheng Zhao, Yu Sun

**Affiliations:** 1 Key Laboratory of Tissue Microenvironment and Tumour, Shanghai Institute of Nutrition and Health, University of Chinese Academy of Sciences, Chinese Academy of Sciences, Shanghai, China; 2 Department of Pharmacology, Institute of Aging Medicine, Binzhou Medical University, Yantai, China; 3 Department of Urology, Zhongshan Hospital, Fudan University, Shanghai, China; 4 Optogenetics & Synthetic Biology Interdisciplinary Research Center, State Key Laboratory of Bioreactor Engineering, Shanghai Frontiers Science Center of Optogenetic Techniques for Cell Metabolism, School of Pharmacy, East China University of Science and Technology, Shanghai, China; 5 Research Unit of New Techniques for Live-cell Metabolic Imaging, Chinese Academy of Medical Sciences, Beijing, China; 6 Department of General Surgery, Pancreatic Disease Institute, Ruijin Hospital, Shanghai Jiao Tong University School of Medicine, Shanghai, China; 7 Department of Pharmacology, Shandong Technology Innovation Center of Molecular Targeting and Intelligent Diagnosis and Treatment, Binzhou Medical University, Yantai, China; 8 Department of Endodontics, Shanghai Stomatological Hospital and School of Stomatology, Fudan University, Shanghai, China; 9 Shanghai Key Laboratory of Craniomaxillofacial Development and Diseases, Fudan University, Shanghai, China; 10 Department of Plastic & Reconstructive Surgery, Shanghai Ninth People’s Hospital, Shanghai Jiao Tong University School of Medicine, Shanghai, China; 11 Buck Institute for Research on Aging, Novato, CA USA; 12 Lawrence Berkeley National Laboratory, University of California, Berkeley, CA USA; 13 Department of Medicine and VAPSHCS, University of Washington, Seattle, WA USA

**Keywords:** Senescence, Cancer microenvironment, Ageing, Metabolism

## Abstract

Senescent cells remain metabolically active, but their metabolic landscape and resulting implications remain underexplored. Here, we report upregulation of pyruvate dehydrogenase kinase 4 (PDK4) upon senescence, particularly in some stromal cell lines. Senescent cells display a PDK4-dependent increase in aerobic glycolysis and enhanced lactate production but maintain mitochondrial respiration and redox activity, thus adopting a special form of metabolic reprogramming. Medium from PDK4^+^ stromal cells promotes the malignancy of recipient cancer cells in vitro, whereas inhibition of PDK4 causes tumor regression in vivo. We find that lactate promotes reactive oxygen species production via NOX1 to drive the senescence-associated secretory phenotype, whereas PDK4 suppression reduces DNA damage severity and restrains the senescence-associated secretory phenotype. In preclinical trials, PDK4 inhibition alleviates physical dysfunction and prevents age-associated frailty. Together, our study confirms the hypercatabolic nature of senescent cells and reveals a metabolic link between cellular senescence, lactate production, and possibly, age-related pathologies, including but not limited to cancer.

## Abstract (web summary)

PDK4-dependent lactate production by senescent stromal cells is shown to promote cancer growth and drug resistance and might have a broader role in the emergence of age-associated diseases.

## Main

Cellular senescence was initially identified as a program characterized with loss of proliferative capacity after exhaustive passaging in culture, which is known as replicative senescence (RS)^1^. Later studies demonstrated that senescence is indeed inducible by multiple types of inherent or environmental stresses, including oncogenic activation (oncogene-induced senescence; OIS) or therapeutic insults (therapy-induced senescence; TIS)^2^. Senescent cells exhibit phenotypic alterations, such as morphological flattening, nuclear expansion, epigenetic reorganization and metabolic alterations^3,4^. They also exhibit cell non-autonomous activities, particularly chronic secretion of numerous pro-inflammatory cytokines and chemokines, a phenotype termed the senescence-associated secretory phenotype (SASP)^5^. The SASP plays a context-dependent role in organismal aging and diverse age-related disorders^4^. The net effect of the SASP is mostly detrimental in advanced life stages, as it contributes to pathological incidence and clinical mortality^6^.

Single-cell profiling at both transcriptomic and proteomic levels suggests that senescent cells undergo intense metabolic reprogramming to maintain their cycle-arrested but viable status, and upregulate the expression of proteins essential to sustain the highly complex, dynamic and heterogeneous SASP^3,7^. In fact, several forms of metabolic stresses can both drive senescence and trigger the SASP. Many drivers of mitochondrial dysfunction contribute to cellular senescence, through disruption of cytosolic nicotinamide adenine dinucleotide (NAD^+^ and NADH), production of reactive oxygen species (ROS) and potentially other mechanisms. Specifically, the mitochondrial dysfunction-associated senescence (MiDAS) phenotype lacks some pro-inflammatory components of the SASP, including those associated with the interleukin (IL)-1-dependent inflammatory arm, yet these cells instead exhibit a distinct set of SASP factors^8^. MiDAS is primarily driven by the accumulation of cytosolic NADH, which is usually oxidized by mitochondria to NAD^+^, causing a reduced NAD^+^/NADH ratio in cytosol and preventing the IL-1-associated SASP via AMPK-mediated p53 activation^8^.

The level of NAD^+^ decreases with age in various senescent cell-residing and metabolically active tissues in a CD38^+^ macrophage-dependent manner, causally linking NAD^+^ exhaustion to both senescence and aging^9^; however, a wider landscape of metabolic activities especially those correlated with glucose consumption and energy production, aspects essential to support the distinct protein synthesis machinery in senescent cells, as well as underlying mechanisms, is largely lacking. In this Article, we aimed to characterize the senescence-associated metabolism and uncovered that that pyruvate dehydrogenase kinase isoform 4 (PDK4), a pyruvate dehydrogenase (PDH) inhibitory kinase modulating glucose metabolic flexibility, is upregulated in senescent cells. Although displaying a reduced NAD^+^/NADH ratio, senescent cells maintain a hypercatabolic activity and produce more pyruvate and lactate, metabolites correlated with enhanced glycolysis. PDK4 upregulation in stromal cells causes elevated cancer aggressiveness, particularly drug resistance, while targeting PDK4 restrains cancer malignancy in vitro, promotes tumor regression in vivo and extends animal post-treatment survival. We further unmasked the implication of lactate in promoting NOX1-dependent ROS production, a process that exacerbates DNA damage and supports the SASP. In advanced stage, suppressing PDK4 activity mitigates physical dysfunction and alleviates frailty, thus improving health conditions. Together, there is an inherent link between cellular senescence, PDK4 upregulation, lactate production and age-related systemic degeneration, which may culminate during chronic disease development such as cancer progression. We propose a senescence-specific metabolic axis involving PDK4, which functionally underlies metabolic reprogramming and may be exploited therapeutically to counteract human aging and age-related pathologies.

## Results

### Genotoxicity induces cellular senescence and PDK4 expression

**Fig. 1:**
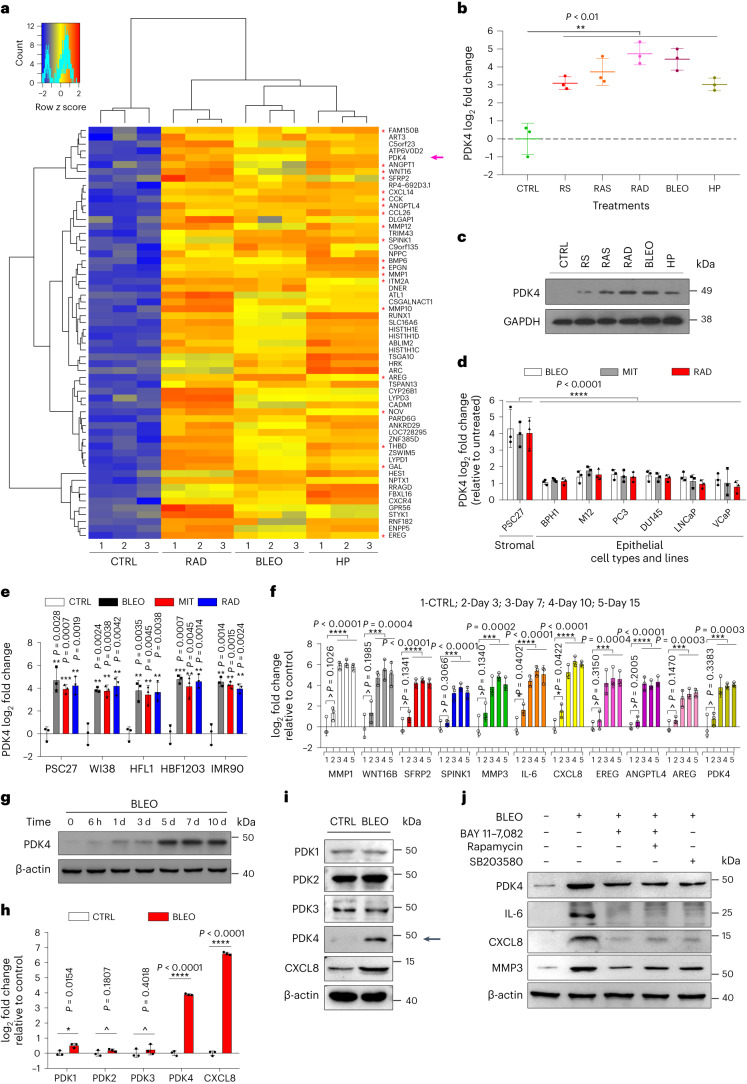
Genotoxicity induces PDK4 upregulation and a full-spectrum SASP. **a**, Expression profiling of primary human stromal line PSC27 by microarray. CTRL, control; RAD, radiation; BLEO, bleomycin. Red highlight indicates SASP factors. Purple arrow indicates PDK4. Microarray data are adapted from Sun et al. with permission from *Nature Medicine*^14^. **b**, Quantitative PCR with reverse transcription to determine PDK4 expression. Signals are normalized to CTRL. RS, replicative senescence; RAS, lentiviral transduction of human oncogene *HRAS*^G12V^. Data are shown as mean ± s.d. in scatter-dot blot. **c**, Immunoblot analysis of PDK4 expression as delineated in **b**. GAPDH, loading control. **d**, Comparative RT–PCR assay of PDK4 expression after treatment of PSC27 or prostate epithelial cells. Signals are normalized to CTRL. BPH1, M12, PC3, DU145, LNCaP and VCaP, human epithelial lines of prostate origin. **e**, Comparative RT–PCR assay of PDK4 expression. WI38, HFL1, HBF1203 and BJ, human stromal lines of different origins; MIT, mitoxantrone. **f**, A time-course RT–PCR assessment of the expression of PDK4 and a subset of typical SASP factors. Numeric numbers indicate the individual days after treatment (indexed at the top line). **g**, Immunoblot measurement of PDK4 expression at the protein level at the individual time points as indicated. β-actin, loading control. **h**, Comparative appraisal of human PDK family expression at transcript level in PSC27. Signals are normalized to untreated sample per gene. CXCL8, experimental control as a hallmark SASP factor. **i**, Immunoblot assessment of the expression of PDK family members at protein level. β-actin, loading control. **j**. Immunoblot analysis of the expression of indicated factors at protein level after treatment of cells with BLEO in the absence or presence of several chemical inhibitors as indicated. β-actin, loading control. Data in **b**,**d**–**f**,**h** are shown as mean ± s.d. and represent three biological replicates. Data in **c**,**g**,**i**,**j** are representative of two independent experiments. *P* values were calculated by one-way ANOVA (**b**,**f**), two-way analysis of variance (ANOVA) (**d**) or two-sided unpaired Student’s *t*-tests (**e**,**f**,**h**). ^*P* > 0.05; *P < 0.05; **P < 0.01; ***P < 0.001; ****P < 0.0001. Source data

**Extended Data Fig. 1:**
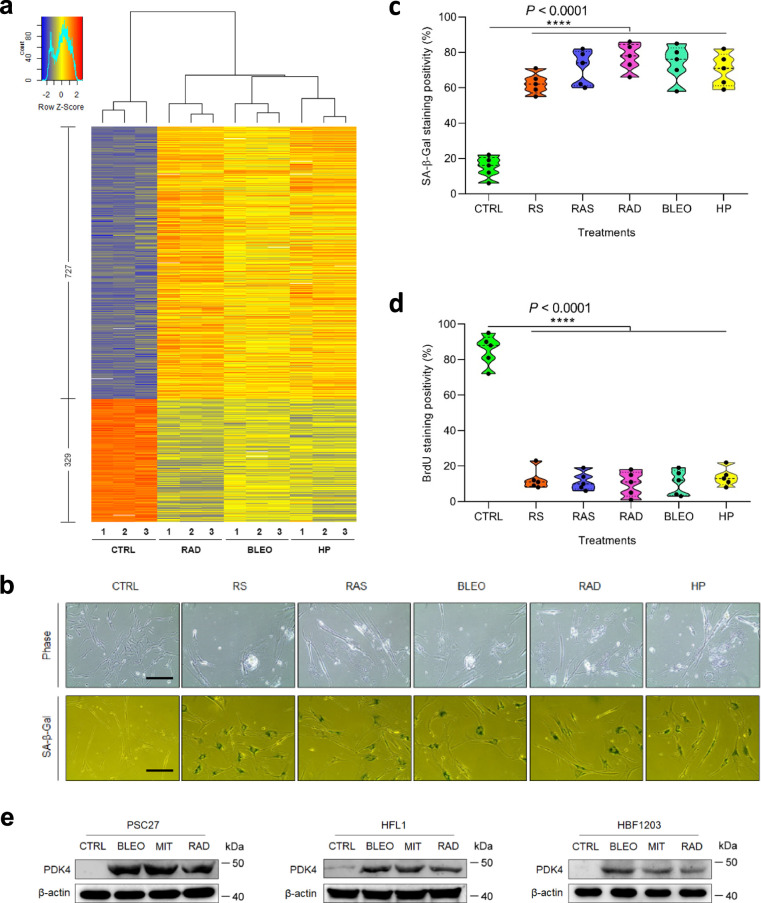
Profiling of global gene expression and senescence induction upon in vitro treatment. **a**. Heat map depiction of expression changes for transcripts up- or downregulated in PSC27 stromal cells after induction of senescence with individual agents. **b**. Senescence assessment with SA-β-Gal staining of human stromal cells that experienced either lentiviral transduction of oncogenic *HRAS*^*G12V*^ (RAS) or anticancer treatments (RS, RAD, BLEO and HP) as indicated. Representative images are shown. Scale bars, 50 μm. **c**. Comparative statistics of senescence positivity upon appraisal with SA-β-Gal staining of stromal cells as described in **b**. **d**. Comparative statistics of senescence induction upon evaluation of BrdU staining positivity of PSC27 cells as described in **b**. **e**. Immunoblot analysis of PDK4 induction in PSC27, HFL1 and HBF1203 cell lines. In each line, cells were exposed to bleomycin (BLEO), mitoxantrone (MIT) or ionizing radiation (RAD). β-actin, loading control. Data in **c** and **d** are presented as violin graphs and represent 5 biological replicates. Data in **e** are representative of two independent experiments. *P* values were calculated by one-way ANOVA (**c**-**d**). **** *P* < 0.0001. Source data

Pyruvate enters the tricarboxylic acid (TCA) cycle through PDH, whereas PDK molecules (PDK1–PDK4) inhibit PDH activity and promotes switch from mitochondrial oxidation to cytoplasmic glycolysis. PDK4 is located in mitochondrial matrix and inhibits the PDH complex by phosphorylating its E1α subunit, thereby regulating glucose metabolism^10^. To date, insights into PDK4 expression in healthy tissue microenvironments and its inducibility in response to stressful insults remain limited, in contrast to former studies documenting PDK4 implications in various cancer types^11–13^. We recently noticed that the stromal cell line PSC27 (of human prostate origin and consisting of mainly fibroblasts but with a minor percentage of non-fibroblast stromal cell lineages) produces a large array of SASP factors upon exposure to cytotoxic insults^14,15^. Notably, PDK4 emerged as an upregulated factor, together with a list of typical SASP components, as revealed by our previous microarray profiling (Fig. 1a and Extended Data Fig. 1a)^14^. To confirm, we expanded by using alternative approaches to induce senescence, including replicative exhaustion (RS) and overexpression of *HRAS*^G12V^ (RAS). We observed comprehensive cellular senescence, phenocopying DNA-damaging agents such as radiation (RAD), bleomycin (BLEO) and hydrogen peroxide (HP) (Extended Data Fig. 1b–d). In each case, there was a pronounced induction of PDK4 in senescent cells (Fig. 1b,c).

Expression analysis of several cell lines of human prostate origin suggested that stromal cells are indeed more PDK4-inducible than cancer epithelial cells, implying a special mechanism supporting PDK4 production in prostate stromal cells (Fig. 1d). Data from several additional fibroblast lines consistently supported a robust induction of PDK4 upon treatment by genotoxic agents (Fig. 1e). Notably, the expression pattern of PDK4 resembled that of hallmark SASP factors, including MMP1, WNT16B, SFRP2, SPINK1, MMP3, IL-6, CXCL8, EREG, ANGPTL4 and AREG, which exhibited a gradual increment until cells entered a platform within 5–7 d after treatment (Fig. 1f,g). In the human PDK family (PDK1–PDK4 isozymes), PDK4 seemed to be the only member readily inducible by genotoxic stress, with a tendency similar to that of CXCL8, an index of SASP expression (Fig. 1h,i). Data from immunoblots after pharmacological treatments targeting p38, mTOR and NF-κB, respectively, suggested that PDK4 expression in senescent cells may be regulated by a intracellular mechanism commonly shared by typical SASP factors such as IL-6, CXCL8 and MMP3 (Fig. 1j).

### PDK4 expression in stroma predicts adverse clinical outcomes

**Extended Data Fig. 2:**
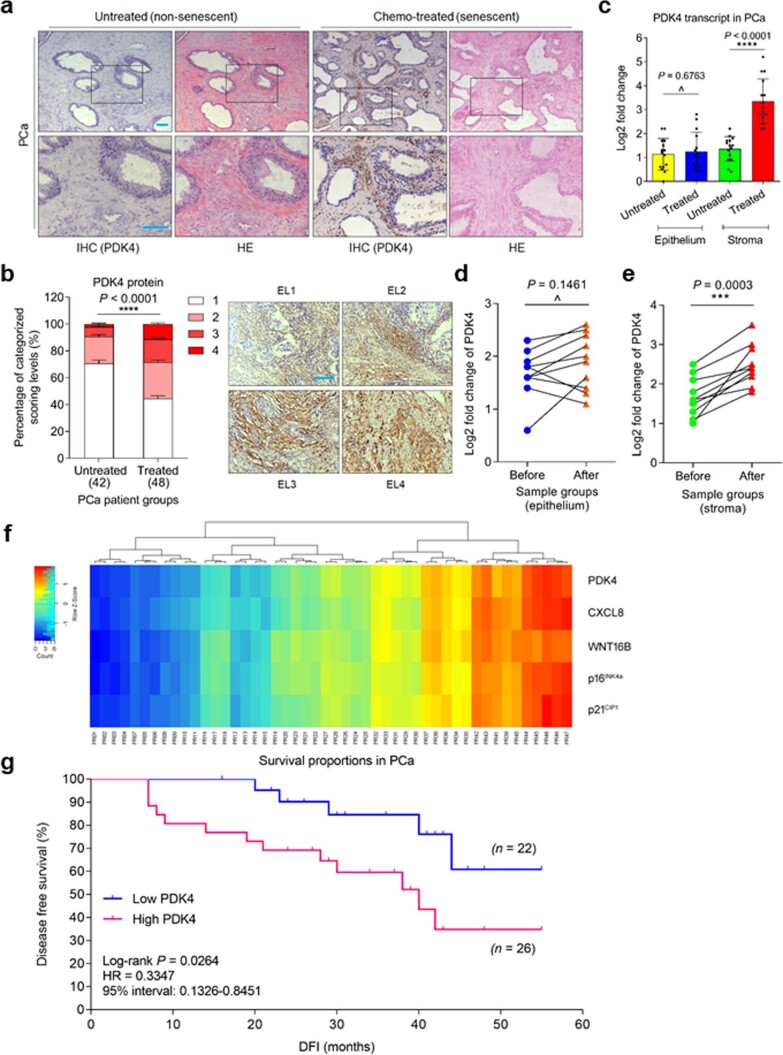
PDK4 is expressed in stroma after chemotherapy and negatively correlated with post-treatment survival. **a**. Representative images of PDK4 expression in biospecimens of human prostate cancer (PCa) patients after histological examination. Left, untreated; right, chemo-treated. Rectangular regions selected in upper images *per* staining amplified into lower images. Scale bars, 100 μm. **b**. Pathological assessment of stromal PDK4 expression in PCa tissues. Patients pathologically assigned into 4 categories *per* IHC staining intensity of PDK4 in stroma. 1, negative; 2, weak; 3, moderate; 4, strong expression. Left, statistical comparison. Right, representative images of each category. EL, expression level. Scale bar, 100 µm. **c**. Boxplot summary of PDK4 transcript expression by qRT-PCR analysis upon laser capture microdissection of cells from tumor and stroma, respectively. Signals normalized to the lowest value in untreated epithelium group, with comparison performed between untreated and treated samples *per* cell lineage. **d**. Comparative analysis of PDK4 expression between epithelial cells before and after chemotherapy. Each dot represents an individual patient, with the data of “before” and “after” connected to allow direct assessment of PDK4 induction in a same patient. **e**. Comparative analysis of PDK4 expression at transcription level between stromal cells collected before and after chemotherapy. Presentation follows **d**. **f**. Pathological correlation between designated factors in the stroma of PCa patients after treatment. Scores from assessment of molecule-specific IHC staining, with expression levels colored to reflect low (blue) via modest (turquoise) and fair (yellow) to high (red) signal intensity. **g**. Kaplan–Meier analysis. Disease-free survival (DFS) stratified according to PDK4 expression. DFS represents length (months) of period calculated from date of PCa diagnosis to point of first time disease relapse. HR, hazard ratio. PCa patients (48 totally) in analysis from treated group of **b**. Data in all bar plots are shown as mean ± S.D. and represent 3 biological replicates. *P* values were calculated by two-sided unpaired Student’s *t*-tests (**c**-**e**), two-way ANOVA (**b**) or Log-rank (Mantel–Cox) tests (**g**). ^, *P* > 0.05. *** *P* < 0.001. **** *P* < 0.0001. Source data

The in vitro results prompted us to further determine whether PDK4 induction occurs within the tumor microenvironment (TME), a pathological entity where many benign stromal cells reside. We first chose to analyze clinical samples of a cohort of patients with prostate cancer (PCa) who developed primary tumors in prostate and underwent neoadjuvant regimen involving a genotoxic agent (mitoxantrone; MIT). PDK4 was found markedly expressed in prostate tissues of these patients after neoadjuvant chemotherapy, but not before (Extended Data Fig. 2a). Basically, in line with our in vitro data, upregulated PDK4 was generally localized in stroma, in a sharp contrast to the adjacent cancer epithelium, which had limited or no staining.

PDK4 production in patient samples pre- versus post-chemotherapy was quantitatively measured by a pre-established pathological assessment procedure, which allowed precise evaluation of a target protein per immunohistochemistry (IHC) staining intensity (Extended Data Fig. 2b). Transcript analysis upon laser capture microdissection (LCM) of cell lineages from primary tissues suggested that PDK4 was more readily induced in stromal rather than cancer compartments (*P* < 0.0001 versus *P* > 0.05) (Extended Data Fig. 2c). To substantiate PDK4 inducibility in vivo, we profiled a subset of patients with PCa whose pre- and post-chemotherapy biospecimens were both accessible, and found notably upregulated PDK4 in stroma, but not cancer epithelium, of each individual post-chemotherapy (Extended Data Fig. 2d,e). We noticed that the dynamics of PDK4 expression in the damaged TME were largely in parallel with that of CXCL8 and WNT16B, two canonical SASP components (Extended Data Fig. 2f). The expression pattern of these factors was largely consistent with that of senescence markers p16^INK4a^ and p21^CIP1^ in tumor foci, suggesting an inherent correlation of PDK4 induction with cellular senescence and the SASP (Extended Data Fig. 2f). Of note, Kaplan–Meier analysis of patients with PCa stratified according to PDK4 expression in the tumor stroma suggested a significant but negative correlation between PDK4 protein level and disease-free survival (DFS) in the treated cohort (*P* < 0.05, log-rank test) (Extended Data Fig. 2g).

The distinct pathological properties of PDK4 in PCa were subsequently reproduced by an extended study involving clinical cohorts of human patients with breast cancer (BCa) (Supplementary Fig. 1a–d). Implicating the functional roles of PDK4, such as acting as a critical regulator of epithelial-to-mesenchymal transition and drug resistance of human cancers^12^, data from gene expression profiling interactive analysis with the cancer genome atlas (TCGA) and genotype-tissue expression (GTEx) databases indicated that PDK4 expression in cancer cells per se is associated with the poor prognosis of some, but not all cancer types (Supplementary Fig. 1e,f). Thereby, in contrast to former studies mainly focusing on PDK4 expression in cancer cells per se, our data consistently suggest that PDK4 induction in the tumor stroma may represent an SASP-associated independent predictor of clinical prognosis, holding the potential to be exploited for stratifying the risk of disease relapse and clinical mortality. Given such a pathological relevance, it is reasonable to speculate that PDK4 production by stroma may have a causal role in senescence-related conditions, such as cancer progression.

### Senescent cells have a distinct glucose metabolism profile

Cancer cells actively reprogram energy metabolism to fuel their expansion and survival, while enhanced mitochondrial function plays important roles in tumor development^16^. One of the major hallmarks of senescent cells is that they remain metabolically active and synthesize a plethora of protein factors (SASP) with the capacity to affect other cells of the host microenvironment^17^. Former studies on senescent cell metabolism revealed elevated levels of both glucose consumption and lactate production during senescence^18^. While increased expression of glucose transporter and glycolytic enzymes during cellular senescence was observed, to date relevant data mostly derived from cancers such as lymphomas and melanomas, or senescent cells induced by activation of oncogenes (OIS) such as BRAF^V600E^, with the metabolic signaling axis specifically elucidated in senescent cancer cells^19,20^. In contrast, the metabolic feature of glucose, a major energy source of senescent cells, especially those of stromal origin and noncancerous entity, as well as influence of such a metabolic profile on surrounding tissue homeostasis, remains yet unclear and merits in-depth understanding.

**Fig. 2:**
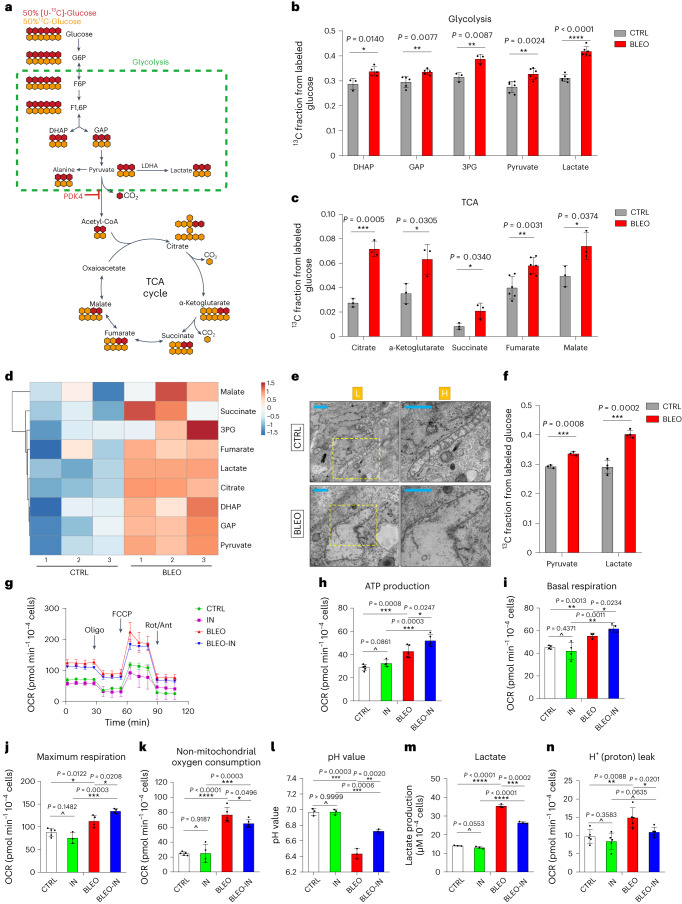
Senescent cells display a distinct glucose metabolism profile. **a**, A schematic molecular roadmap briefly outlining the landscape of glucose metabolism in mammalian cells. **b**, Partial metabolic profiling (glycolysis) of senescent cells induced by BLEO and incubated with uniformly labeled [U-^13^C_6_]-glucose. Results from GC–MS analysis of metabolites as indicated. **c**, Partial metabolic profiling (TCA cycle) of senescent cells induced by BLEO and incubated with uniformly labeled [U-^13^C_6_]-glucose. Results from GC–MS analysis of metabolites as indicated. **d**, Heat map depicting changes of glucose catabolism-associated metabolites as measured for senescent cells by GC–MS. **e**, Representative TEM images showing the ultrastructural profile of mitochondria in PSC27. L, low resolution; H, high resolution. Scale bars, 1.0 μm. **f**, Measurement of extracellular fluids with an XF24 Extracellular Flux Analyzer. Pyruvate and lactate were assayed. **g**, OCR of stromal cells was measured using an XF24 Extracellular Flux Analyzer. All Seahorse data were normalized with cell numbers, with metabolic parameters automatically calculated by WAVE software equipped in Seahorse. OCR, oxygen consumption rate; Oligo, oligomycin; FCCP, carbonyl cyanide 4-(trifluoromethoxy) phenylhydrazone; Rot, rotenone; Ant, antimycin; IN, PDK4-IN (PDK4 inhibitor, 5 μM). **h**, Measurement of ATP production by PSC27. ATP production measured as (last rate measurement before Oligo injection) minus (minimum rate measurement after Oligo injection). **i**, Assessment of basal respiration as an essential element of the senescence-associated metabolism program. **j**, Examination of maximal respiration as another fundamental element of the senescence-associated metabolism program. **k**, Assessment of non-mitochondrial oxygen consumption in stromal cells. **l**, Measurement of pH values in stromal cells. **m**, Determination of lactate production in stromal cells. **n**, Examination of the leak of H^+^ (proton) from mitochondria of stromal cells. Data in all bar plots are shown as mean values ± s.d. and represent 3 (**l**,**m**) or 3–6 (**b**,**c**,**f**–**k**,**n**) biological replicates. *P* values were calculated by two-sided unpaired Student’s *t*-tests (**b**,**c**,**f**,**h**–**n**). ^*P* > 0.05; *P < 0.05; **P < 0.01; ***P < 0.001; ****P < 0.0001. Source data

**Extended Data Fig. 3:**
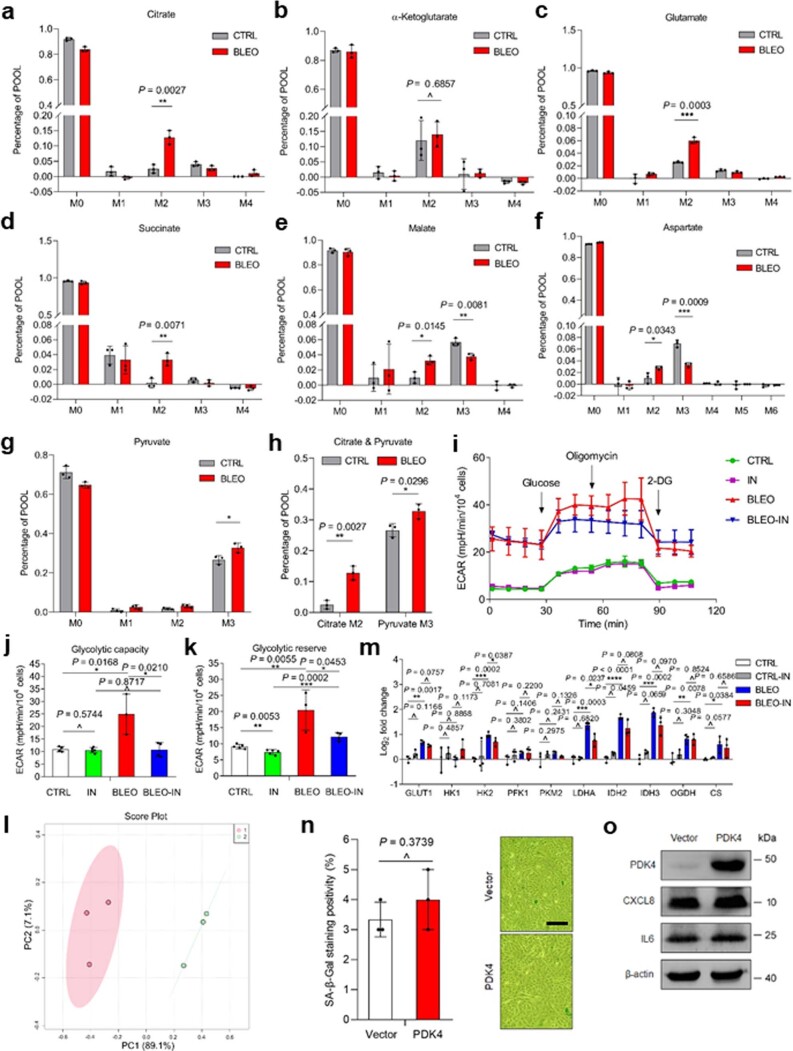
Senescent cells manifest glucose metabolic properties distinct from normal and cancer cells. **a-g**. Analysis of gas chromatography-mass spectrometry (GC–MS) to determine isotope labeling of individual specific metabolites. Retrieved data comprised mass intensities for the lightest isotopomer (without any heavy isotopes, M0) and isotopomers with increasing unit mass (up to M6) relative to M0. Mass distributions normalized by dividing by the sum of M0 to M6 and corrected for the natural abundance of heavy isotopes of the elements H, N, O, Si and C, with matrix-based probabilistic methods as described in Methods, and implemented in MATLAB. **h**. Statistic comparison of citrate M2 and pyruvate M3 in control and senescent cells induced by BLEO treatment. **i**. Extracellular acidification rate (ECAR) profile of stromal cells was determined using a Glycolysis Stress Test kit. **j**. Measurement of the glycolyic capacity of stromal cells. Data derived from ECAR assays and presented in mpH/min. **k**. Assessment of the glycolytic reserve of stromal cells. Data derived from ECAR assays and presented in mpH/min. **l**. Principal component analysis (PCA) of global metabolites assayed by metabolite profiling approaches. **m**. Quantitative RT-PCR to examine expression of glucose uptake and metabolism-associated genes after PSC27 cells were subject to individual treatment as indicated. **n**. Evaluation of cellular senescence of PSC27 cells by SA-β-Gal staining. Left, comparative statistics. Right, representative images. Scale bar, 100 μm. **o**. Immunoblot analysis of PDK4, CXCL8, IL6 expression in stromal cells expressing exogenous PDK4. Vector, control cells transduced with an empty vector. PDK4, cells transduced with a PDK4 construct. Data in all bar plots are shown as mean ± S.D. and represent 3-5 biological replicates. Data in **o** are representative of two independent experiments. *P* values were calculated by two-sided unpaired Student’s *t*-tests (**a**-**h**, **j**-**k**, **m**-**n**). ^, *P* > 0.05. * *P* < 0.05. ** *P* < 0.01. *** *P* < 0.001. **** *P* < 0.0001. Source data

Glucose is the primary carbon source to the TCA cycle, followed by glutamate and aspartate (non-protonatable amino acids as glutamine or asparagine, respectively) as secondary sources (Fig. 2a)^21^. We first interrogated the metabolic pattern of glucose upon uptake by senescent cells, as glucose acts as a principal contributor to TCA cycle when cells enter senescence, a stage allowing cells to sustain metabolic activity^22^. Experimental data from analysis of mitochondrial dynamics and cellular bioenergetics with gas chromatography–mass spectrometry (GC–MS) indicated notably elevated glycolytic activity in senescent human stromal cells, as reflected by enhanced production of metabolites, including but not limited to dihydroxyacetone phosphate (DHAP), glyceraldehyde-3-phosphate (GAP) and 3-phosphoglycerate (3-PG) (Fig. 2b). Increased levels of GAP and 3-PG imply further utilization of a number of middle metabolites, such as citrate, α-ketoglutarate, glutamate, succinate, fumarate and malate in the TCA cycle, all metabolically derived from pyruvate and substantiated by metabolic profiling with GC–MS (Fig. 2c and Extended Data Fig. 3a–g).

To establish these metabolic changes, we employed MIT, RAD and FCCP, the latter a potent uncoupler of oxidative phosphorylation (OXPHOS) in mitochondria that disrupts ATP synthesis by transporting protons across cell membranes, to treat cells in parallel assays. The results suggested that both MIT and RAD, genotoxic agents causing remarkable PDK4 induction (Extended Data Fig. 1e), were able to reproduce the effect of BLEO, whereas such metabolic fluctuations were not observed in FCCP-treated cells (Supplementary Fig. 2a–f). The differential impact of these agents on metabolic activities were largely consistent with PDK4 induction in affected cells (Supplementary Fig. 2g). Thus, PDK4 upregulation and metabolic alterations were intimately correlated and engaged upon genotoxicity-induced cellular senescence, rather than simply a stress response caused by mitochondrial damage. Notably, bioactivities of both glycolysis and the TCA cycle were significantly enhanced in senescent cells, as reflected by metabolic profiling with assays of stable isotope labeling with a uniformly labeled U-^13^C_6_ glucose tracer and fractioning of metabolites derived from labeled glucose and revealed by GC–MS (Fig. 2d).

Entry of glucose-derived and PDH-catalyzed flow of carbon into the TCA cycle generates isotopomer species with two labeled carbons (M2), whereas species with more labeled carbons (M3 and M4) arise from the addition of labeled acetyl-CoA to labeled oxaloacetate produced by TCA cycling (Fig. 2a)^23^. Compared to their proliferating counterparts, senescent cells displayed an increased rather than decreased citrate M2/pyruvate M3 ratio, further implying enhanced TCA cycle activity alongside the simultaneously increased glycolytic capacity (Extended Data Fig. 3h), a feature that makes them remarkably distinct from various cancer cell types.

We noticed that these metabolic changes were accompanied by substantial perturbations in mitochondrial ultrastructure of senescent cells, particularly enlarged sizes and abnormal shapes as revealed by transmission electron microscopy (TEM), a phenomenon indicative of ultrastructural damage of mitochondria and suggesting potential mitochondrial dysfunction associated with oxidative stress upon cellular senescence (Fig. 2e). Appearance of ultrastructural changes were indeed accompanied by a significant increase of mitochondrial number and mass in senescent cells, relative to their proliferating counterparts (Supplementary Fig. 2h,j). These observations are largely in line with former studies regarding abnormal phenotypes of mitochondria including mass, dynamics and structure upon senescence^24^.

We next measured the levels of extracellular fluids. Notably, amounts of both pyruvate and lactate released to the extracellular space were considerably enhanced in senescent cells (Fig. 2f). These changes were accompanied by alterations in oxygen consumption rate (OCR) and extracellular acidification rate (ECAR) as determined by an XF24 Extracellular Flux Analyzer, suggesting elevated metabolic activities associated with glucose utilization (Fig. 2g and Extended Data Fig. 3i–k). Correspondingly, we observed elevated ATP production, basal respiration, maximum respiration in senescent cells, a pattern indicative of tight connection of the TCA cycle and oxidative phosphorylation (OXPHOS) but further promoted when PDK4-IN-1, an anthraquinone derivative and a potent inhibitor of PDK4 (PDK-IN hereafter)^25^, was applied to culture (Fig. 2h–j); however, treatment with PDK4-IN reversed changes in non-mitochondrial oxygen consumption, pH fluctuation, lactate production and H^+^ (proton) leak, with the overall metabolic data validated by principal-component analysis scores (PC1 versus PC2) (Fig. 2k–n and Extended Data Fig. 3l). These alterations occurred in parallel with expression changes of glucose uptake-associated molecules and metabolism-related enzymes, including glucose transporter 1 (GLUT1), hexokinase 2 (HK2), lactate dehydrogenase A (LDHA), isocitrate dehydrogenase 2 (IDH2), isocitrate dehydrogenase 3 (IDH3), oxoglutarate dehydrogenase (OGDH) and citrate synthase (CS) (Extended Data Fig. 3m). HK2 and LDHA are glycolysis-related factors, whereas IDH2, IDH3, OGDH and CS are TCA cycle-involving enzymes. As overexpression of PDK4 per se in normal cells neither caused or abrogated senescence, nor affected the SASP (Extended Data Fig. 3n,o), we reasoned that senescent cell metabolism was correlated with and likely underpinned by expression of key factors involved in glucose consumption and linked with production of pyruvate, lactate and multiple other metabolites. Notably, elevated levels of glycolysis and oxidative phosphorylation were simultaneously observed, suggesting essentially reprogrammed glucose metabolism upon senescence.

Former studies reported that senescent cells exhibit increased glucose transporter and glycolytic enzyme expression after chemotherapeutic treatment^19^, a feature basically confirmed by our data (Extended Data Fig. 3m). Steady-state glucose concentrations tend to be higher in senescent cells than their proliferating counterparts, suggesting an elevated glucose avidity upon senescence. These findings are confirmed by metabolomics profiling, which underscores the global catabolic nature of senescent cells. To expand, we examined the metabolic profiles of HFL1 and HBF1203, two stromal lines derived from human lung and breast tissues, respectively, and holding potential to upregulate PDK4 expression upon senescence (Extended Data Fig. 1e). Indeed, both lines reproduced the senescence-associated metabolic changes observed in PSC27 (Supplementary Fig. 3a–h). Together, senescent cells develop a distinctive hypermetabolic phenotype characterized of enhanced glycolysis, TCA cycle activity and ATP-boosting OXPHOS. Increased energy production is a common denominator of senescent cells, which exhibit specific utilization of energy-generating metabolic pathways, a phenomenon partially reminiscent of the ‘Warburg effect’ observed in cancer cells^19^.

### Senescent cells produce lactate via PDK4 expression

**Fig. 3:**
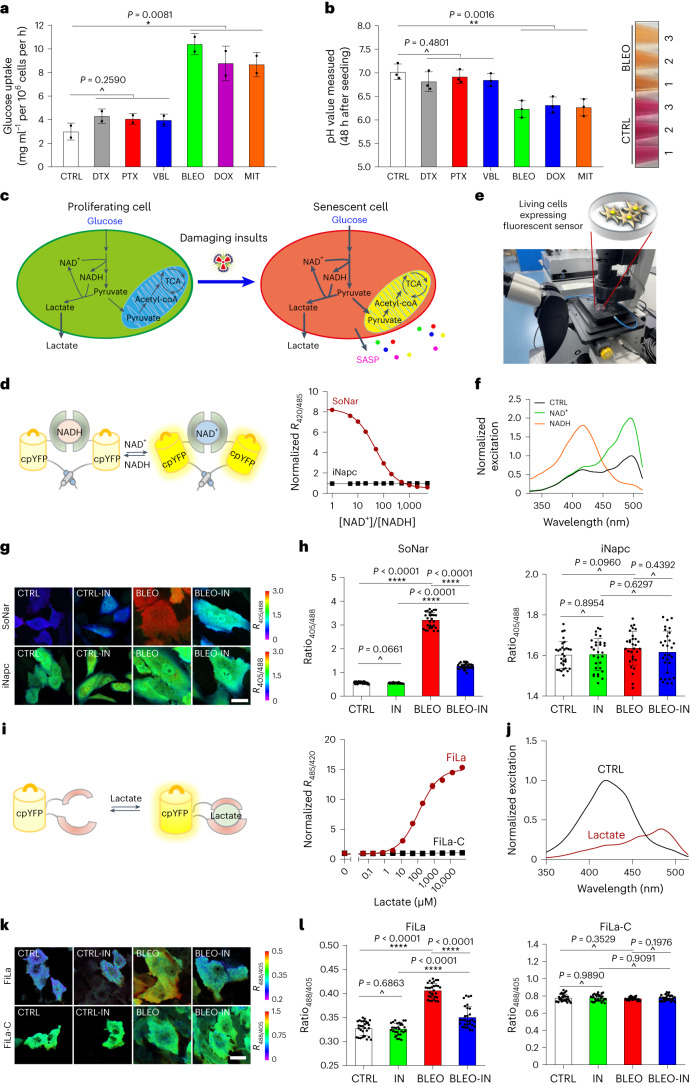
Senescent cells exhibit altered NAD^+^/NADH and lactate production. **a**, Glucose uptake measurement of PSC27 upon senescence. DTX, docetaxel; PTX, paclitaxel; VBL, vinblastine; BLEO, bleomycin; DOX, doxorubicin; MIT, mitoxantrone. **b**, Examination of pH value of cells treated in **a**. Representative images of CM from proliferating and senescent cells, respectively (right). **c**, Schematic illustration of potential changes in cell metabolic activities during stress-induced senescence. **d**, Graphic model for design of SoNar. Fluorescence ratios plotted against the NAD^+^/NADH ratio at 400 μM total NAD (right). Fluorescence ratios normalized to the CTRL condition (*n* = 3). iNapc, a control sensor, which displays pH sensitivities similar to SoNar. **e**, Technical overview for in vitro imaging of living cells with confocal laser-scanning microscopy. **f**, Excitation spectra of purified SoNar in the control condition (black) and after addition of 20 μM NAD^+^ (green) or NADH (orange), normalized to the peak intensity in control. Emission measured at 530 nm. **g**, Fluorescence imaging of SoNar in CTRL and senescent (BLEO) cells, in the absence or presence of PDK inhibitor (IN). Scale bar, 20 μm. **h**, Quantification of SoNar or iNapc fluorescence (*n* = 30 cells). SoNar (left). iNapc (right). **i**, Schematic representation of molecular design for lactate sensor FiLa. Lactate titration curves (right). Data are normalized to initial value (*n* = 3). FiLa-C, a control sensor, which displays pH sensitivities similar to FiLa. **j**, Excitation spectra of purified FiLa in control (black) and saturated with lactate (dark red). **k**, Fluorescence imaging of FiLa in CTRL and senescent (BLEO) cells. Scale bar, 20 μm. **l**, Quantification of FiLa (left) and FiLa-C (right) fluorescence (*n* = 30 cells). Data in all bar plots are shown as mean ± s.d. and represent 3 (**a**,**b**) or 30 biological replicates (**h**,**l**). Pseudocolors were employed to allow straightforward visualization of the fluorescence images (**g**,**k**). *P* values were calculated by one-way ANOVA (**a**,**b**) or two-sided unpaired Student’s *t*-tests (**h**,**l**). ^*P* > 0.05; *P < 0.05; **P < 0.01; ***P < 0.001; **** *P* < 0.0001. Source data

**Extended Data Fig. 4:**
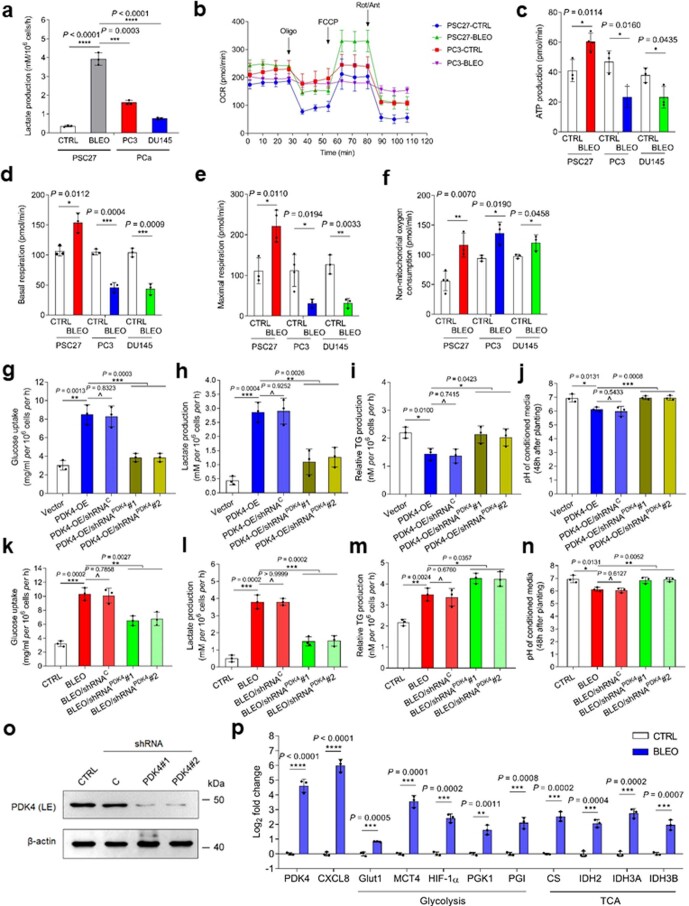
Senescent stromal cells reprogram glucose metabolism via PDK4 expression. **a**. Measurement of lactate production by comparing proliferating and senescent PSC27 cells as well as stromal and typical PCa lines (PC3 and DU145). **b**. OCR assessment of human stromal cells with XF24 extracellular flux analyzer. Oligo, oligomycin. FCCP, carbonyl cyanide 4-(trifluoromethoxy) phenylhydrazone. Rot, rotenone. Ant, antimycin. **c**. Examination of ATP upon bleomycin (BLEO)-induced senescence. **d**. Evaluation of basal respiration of indicated lines. **e**. Appraisal of maximal respiration of indicated lines. **f**. Assessment of non-mitochondrial oxygen consumption of indicated lines. **g**. Glucose uptake measurement of PSC27 cells transduced with a PDK4 construct or depleted of PDK4 via small hairpin RNA. OE, overexpression. C, scramble control. **h**. Lactate production assessment of PSC27 sublines as described in **g**. **i**. Relative TG production assay of PSC27 sublines as described in **g**. **j**. Determination of the pH of conditioned media of PSC27 sublines as described in **g**. **k**. Glucose uptake measurement of PSC27 cells upon BLEO-induced senescence (TIS) in the presence or absence of PDK4, the latter mediated by shRNA knockdown. **l**. Lactate production measurement of PSC27 cells as described in **k**. **m**. Relative TG production assay of PSC27 cells as described in **k**. **n**. Determination of the pH of conditioned media of PSC27 cells as described in **k**. **o**. Immunoblot assessment of PDK4 expression upon transduction of cells with constructs encoding shRNAs. C, scramble. LE, long exposure. **p**. Comparative RT-PCR assay of PDK4, CXCL8, glycolysis-related genes (Glut1, MCT4, HIF1α, PGK1 and PGI) as well as and TCA-related genes (CS, IDH2, IDH3A and IDH3B) expression in human stromal cells 7 d after treatments. Data in all bar plots are shown as mean ± S.D. and represent 3 (**a**, **d**, **g-n**, **p**) or 3-5 (**b, c**, **e**, **f**) biological replicates. Data in **o** are representative of two independent experiments. *P* values were calculated by two-sided unpaired Student’s *t*-tests (**a**-**n**, **p**) or one-way ANOVA (**g**-**n**). ^, *P* > 0.05. * *P* < 0.05. ** *P* < 0.01. *** *P* < 0.001. **** *P* < 0.0001. Source data

Previous studies indicated that senescent cells are in a hypermetabolic status, more specifically, these cells display a hypercatabolic nature^19^, thus prompting us to interrogate whether these cells have a glucose uptake capacity distinct from proliferating cells. To address this, we performed another set of metabolic assays. Not surprisingly, a significant increase of glucose uptake by senescent cells was observed, although changes were preferentially detected upon genotoxicity-induced senescence (GIS), which usually involves DNA damage (Fig. 3a). The pH of conditioned medium (CM) from senescent cells was markedly decreased, a property that again seemed to be more dramatic for GIS (Fig. 3b). Given the results indicative of elevated acidification as revealed by ECAR assay (Extended Data Fig. 3i), we reasonably speculated extracellular formation of an acidic microenvironment by senescent cells, whose metabolism seemed to be markedly reprogrammed and characterized with increased secretion of acidic metabolites. Notably, senescent cells generated an increased amount of lactate, in contrast to their cycling controls (Extended Data Fig. 4a). Cancer cells exhibit increased lactate production, OCR level and ATP output, a series of metabolic changes correlated with enhanced glycolysis^26,27^. We noticed that many relevant activities of senescent cells were even higher than their cancer cell counterparts selected as of the same organ origin (herein, prostate), such as PC3 and DU145, although with several key features showing changes evidently opposite to those of examined cancer lines (Extended Data Fig. 4a–f).

PDK4 is a key enzyme involved in regulation of glucose and fatty acid metabolism as well as tissue homeostasis, while its overexpression inactivates the PDH complex by phosphorylating the targets and contributes to metabolic flexibility. We assessed the influence of PDK4 expression by transducing a PDK4 construct to human stromal cells and noticed significantly altered metabolic profile, including glucose uptake, lactate and triglyceride (TG) production, although these changes were largely reversed upon genetic eliminated of PDK4 (Extended Data Fig. 4g–i). A decreased pH of the CM was observed upon PDK4 overexpression in proliferating cells, but subject to counteraction by PDK4 suppression (Extended Data Fig. 4j). We further measured these parameters with BLEO-induced senescent cells and found markedly increased glucose uptake, lactate and TG production, but reduced pH of the CM (Extended Data Fig. 4k–n); however, almost all these metabolic changes were substantially reversed upon PDK4 depletion, except TG levels, a case suggesting PDK4-mediated antagonism against TG synthesis throughout the TCA cycle in senescent cells (Extended Data Fig. 4k–o). We noticed that factors functionally supporting glycolysis and TCA, including GLUT1, MCT4, HIF1α, PGK1, PGI, CS, IDH2, IDH3A and IDH3B were concurrently upregulated upon GIS, further indicating an overall enhancement of cellular metabolism (Extended Data Fig. 4p).

NAD^+^ and its reduced form, NADH, are pivotal coenzymes for redox reactions and play critical roles in energy metabolism^28^. The intracellular level of NAD^+^ is frequently altered during aging and upon age-related pathologies. We previously generated SoNar, an intensely fluorescent, rapidly responsive, pH-resistant and genetically encoded sensor for tracking subtle changes in cytosolic NAD^+^ and NADH redox states by imaging and quantifying the NAD^+^/NADH ratio in living cells and in vivo^29^, but the metabolic profile NAD^+^ and NADH in senescent cells remains largely undefined (Fig. 3c). We first measured the intracellular NAD^+^/NADH redox state of PSC27 cells utilizing SoNar’s fluorescence (Fig. 3d–f). The data indicated a declined NAD^+^/NADH ratio upon TIS (evidenced by increased NADH/NAD^+^), but essentially subject to reversal by the PDK4 inhibitor, suggesting an elevated reduction of NAD^+^ to NADH, a process accompanied by enhanced glycolysis (Fig. 3g,h). We further designed FiLa, a highly responsive, ratiometric and genetically encoded lactate sensor to monitor the production and consumption of lactate at subcellular resolution^30^ (Fig. 3i,j). We observed a marked increase in cytosolic lactate upon senescence, albeit essentially abrogated in the case of PDK4 suppression (Fig. 3k,l). The results from fluorescence sensors suggest that lactate production augments in parallel to the NAD^+^/NADH ratio drop in senescent cells, whereas both changes are correlated with PDK4 activity. Our data not only disclose the concurrent fluctuation of NAD^+^/NADH conversion and lactate generation, but further substantiate the central role of PDK4 in orchestrating a metabolic profile specifically associated with cellular senescence.

### PDK4^+^ stromal cells enhance cancer cell malignancy

**Fig. 4:**
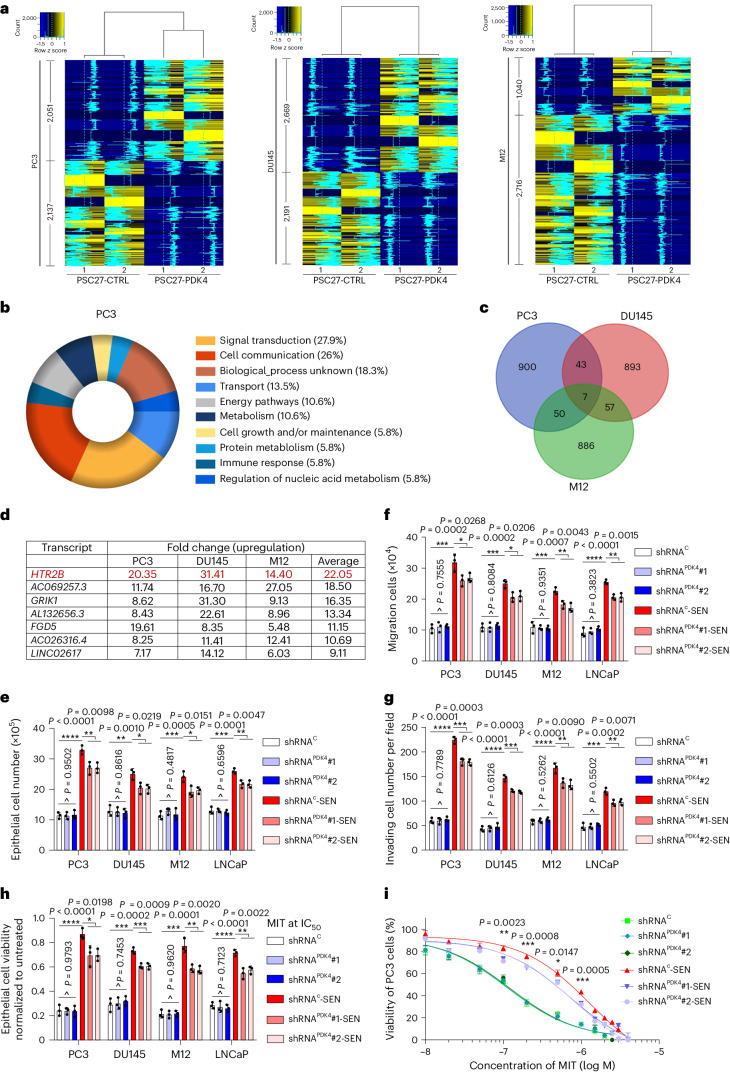
Stromal PDK4 expression enhances cancer cell malignancy. **a**, Heat map depicting differentially expressed human transcripts in PCa lines after a 3-d culture with the CM of PSC27 cells overexpressing PDK4 (PSC27-PDK4). In contrast to cancer cells cultured with control CM (PSC27-CTRL), the number of genes up- and downregulated per PCa line are indicated. Intensity of tracing lines consistent with the relative expression fold change averaged per up- or downregulated genes. **b**, Graphical visualization of pathways by Gene Ontology profiling (pie chart depicting biological processes). Genes significantly enriched in upregulated list were sorted according to fold change in PC3 cells exposed to the CM of PSC27-PDK4 cells. **c**, Venn diagram displaying the overlap of transcripts co-upregulated in PC3, DU145 and M12 cells (per 2 or 3 lines) upon treatment with the CM from PSC27-PDK4 in contrast to those treated with the CM of PSC27-CTRL. **d**, Summary of transcripts co-upregulated in PCa lines (top ranked, with a fold change ≥ 5.0 and false discovery rate (FDR) < 0.01) upon treatment with the CM of PSC27-PDK4. Red highlight indicates HTR2B. **e**, Measurement of PCa line proliferation in different conditions. Human PDK4 was knocked down from PSC27 cells. C, scramble control. **f**, Examination of migration activity in different conditions. Cells were treated in a manner similar to that described in **e**. **g**, Evaluation of invasion ability in different conditions. Cells were treated in a manner similar to that described in **e**. **h**, Determination of resistance to MIT upon exposure to the CM of PSC27. MIT, mitoxantrone, a chemotherapeutic agent applied at the half-maximum inhibitory concentration (IC_50_) concentration per PCa line. **i**, Dose–response curves plotted from MIT-based viability assays of PC3 exposed to the CM of PSC27 and treated by MIT. *P* values indicate the significance of difference between shRNA^C^-SEN and shRNA^PDK4^-SEN groups. Data in all bar and curve plots (**e**–**i**) are shown as mean values ± s.d. and averaged from three biological replicates. *P* values were calculated by two-sided unpaired Student’s *t*-tests (**e**–**h**) or one-way ANOVA (**e**–**i**). ^*P* > 0.05. *P < 0.05. **P < 0.01. ***P < 0.001. Source data

**Extended Data Fig. 5:**
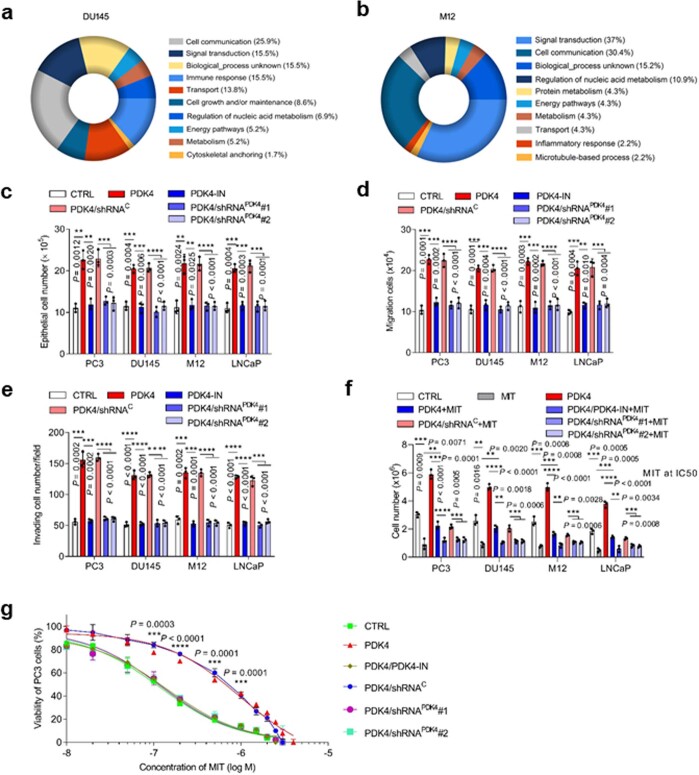
PDK4^+^ stromal cells induce substantial changes of PCa cell expression and malignancy. **a**. Graphical visualization of pathways by GO profiling (pie chart depicting biological processes). Those significantly enriched genes in the upregulated list were sorted according to their fold change in DU145 cells exposed to the CM of PSC27-PDK4 stromal cells. **b**. Graphical visualization of pathways by GO profiling in a manner resembling that represented in **a**. Those significantly enriched genes in the upregulated list were sorted according to their fold change in M12 cells exposed to the CM of PSC27-PDK4 stromal cells. **c**. Appraisal of the proliferation capacity of PCa lines upon exposure to the CM of PSC27 stromal cells. PDK4 knockdown was performed through shRNA-encoding constructs. C, scramble control. **d**. Measurement of the migration ability of PCa lines upon exposure to the CM of PSC27 stromal cells. **e**. Examination of the invasiveness of PCa lines upon exposure to the CM of PSC27 stromal cells. **f**. Determination of the resistance of PCa lines to MIT upon exposure to the CM of PSC27 stromal cells. MIT, mitoxantrone, a chemotherapeutic agent applied at the IC50 concentration *per* cell line established prior to the assay. **g**. Dose-response curves (non-linear regression/curve fit) plotted from MIT-based viability assays of PC3 exposed to the CM of PSC27 stromal cells and treated by a range of concentrations of MIT. *P* values indicate the significance of difference between PDK4/shRNA^C^ and PDK4/shRNA^PDK4^ groups. Data in all bar and curve plots (**c**-**g**) are shown as mean ± S.D. and represent 3 biological replicates. All *P* values were calculated by two-sided unpaired Student’s *t*-tests (**c**-**f**) or one-way ANOVA (**c**-**g**). ^, *P* > 0.05. *, *P* < 0.05. **, *P* < 0.01. ***, *P* < 0.001. ****, *P* < 0.0001. Source data

We next sought to determine the influence of PDK4-expressing stromal cells on their surrounding microenvironment. As PSC27 is originally derived from the human prostate, we first chose to examine PCa cells. PSC27-derived CM was prepared to treat PCa cells in culture, with cancer cells subject to genome-wide analysis. Data from RNA sequencing (RNA-seq) indicated 4,188 transcripts significantly upregulated or downregulated (fold change > 2, *P* < 0.05) in PC3 cells, with 4,860 and 3,756 transcripts changed in DU145 and M12 cells, respectively (Fig. 4a). We noticed remarkable and comprehensive changes in the biological processes of PCa cells, as evidenced by considerably affected activities in signal transduction, cell communication, intracellular transport, energy pathways and metabolism regulation (Fig. 4b and Extended Data Fig. 5a,b). The data suggest a salient capacity of PDK4-expressing stromal cells in reprogramming transcriptomic expression of recipient cancer cells through CM production.

Among the transcripts significantly upregulated by PSC27 cell-derived CM (*P* < 0.05, FDR < 0.01, top 1,000 shown per PCa line; Supplementary Table 1), there were seven transcripts showing up and commonly expressed by PC3, DU145 and M12 cells (fold change > 4, *P* < 0.01) (Fig. 4c,d). Specifically, HTR2B seemed to be the most upregulated in PCa lines upon exposure to PDK4^+^ stromal cell-derived CM, validating future efforts to determine whether it accounts for a principal force driving malignant changes of recipient cancer cells. Further data from in vitro assays indicated significantly enhanced capacity of proliferation, migration and invasion of individual examined PCa lines upon exposure to PDK4^+^-stromal cell CM (Extended Data Fig. 5c–e). More notably, we found that resistance of these cells to MIT, a DNA-targeting chemotherapeutic agent administered to patients with cancer, including those developing PCa^31,32^, was also increased (Extended Data Fig. 5f). Survival curves of cancer cells under genotoxic stress of MIT displayed an apparent shift toward higher concentrations of this drug, as exemplified by the case of PC3 (Extended Data Fig. 5g). Of note, either suppression of PDK4 activity by PDK4-IN or knockdown of PDK4 via small hairpin RNAs (shRNAs) remarkably deprived cancer cells of these gain-of-functions conferred by PDK4^+^-stromal cell CM, substantiating the key role of PDK4 in governing the potential of PDK4^+^-stromal cell CM to modify cancer cell behaviors (Extended Data Fig. 5c–g). We further collected the CM from senescent stromal cells to treat PCa lines, with resultant data suggesting the capacity of senescent stromal cell-produced CM in conferring cancer cells with enhanced proliferation, migration, invasion and chemoresistance, a tendency weakened upon PDK4 elimination from stroma cells (Fig. 4e–i). These results consistently support a key role of PDK4 in mediating the generation of senescent cell-specific extracellular niche, which substantially promotes recipient cancer cell malignancy, albeit relevant mechanisms need future substantiation.

**Extended Data Fig. 6:**
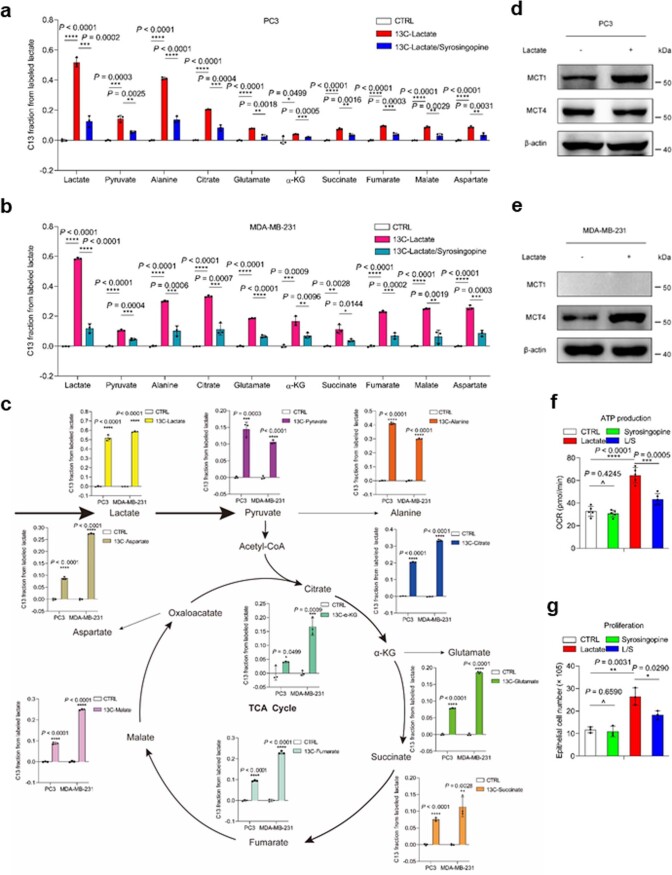
Metabolic landscape of cancer cells upon uptake of exogenous lactate. **a**. Partial metabolic profiling (glycolysis) of recipient prostate cancer cells (PC3) exposed to exogenous lactate pre-labeled with a stable isotope tracer [U-^13^C_6_]. Results from gas chromatography-mass spectrometry (GC–MS) analysis of metabolites including lactate, pyruvate, alanine, citrate, glutamine, α-KG, succinate, fumarate, malate and aspartate are shown. Syrosingopine, a dual inhibitor of lactate transporters including MCT1 and MCT4. **b**. A partial metabolic profiling (glycolysis) of recipient breast cancer cells (MDA-MB-231) exposed to exogenous lactate pre-labeled with the stable isotope tracer [U-^13^C_6_]. Results derived from cell treatments and gas chromatography-mass spectrometry (GC–MS) analysis of metabolites as indicated in **a**. **c**. A landscape map showing overall profile of glucose metabolism-associated catabolic metabolites in PC3 and MDA-MB-231 cells upon uptake of [U-^13^C_6_]-labeled lactate in culture. **d**. Immunoblot assessment of MCT1 and MCT4 expression in PC3 cells upon exposure to [U-^13^C_6_]-labeled lactate in culture. β-actin, loading control. **e**. Immunoblot assessment of MCT1 and MCT4 expression in MDA-MB-231 cells upon exposure to [U-^13^C_6_]-labeled lactate in culture. β-actin, loading control. **f**. Measurement of ATP production by PC3 cells. After exposure to lactate (10 mM), syrosingopine (10 μM), or both, cells were subject to ATP production appraisal. ATP production was measured as (last rate measurement before oligomycin injection) minus (minimum rate measurement after oligomycin injection). L, lactate. S, syrosingopine. **g**. Measurement of PC3 cell proliferation. Cells treated as described in **f** before examination of proliferation in culture conditions. L, lactate. S, syrosingopine. Data in all bar plots are shown as mean ± S.D. Data in **a**-**c, g** represent 3 biological replicates, while those in **f** represent 5 biological replicates. Data in **d**-**e** are representative of two independent experiments. All *P* values were calculated by two-sided unpaired Student’s *t*-tests (**a**-**c, f**-**g)**. *, *P* < 0.05. **, *P* < 0.01. ***, *P* < 0.001. ****, *P* < 0.0001. Source data

As export of lactate into the microenvironment maintains intracellular pH and recycles NADH, both essential for sustaining metabolic activities, we queried the human cancer cell-associated uptake pattern and utilization profile of exogenous lactate. To this end, we chose to supplement with L-[1-^13^C] lactate (10 mM) in culture, a condition that largely mimics the concentration of lactate produced by senescent stromal cells and allows tracing and fractioning of metabolites of recipient cells. The input of L-[1-^13^C] lactate in culture led to intracellular enrichment of exogenous lactate in both PC3 and MDA-MB-231 cells (higher than 5.0%) (Extended Data Fig. 6a,b). The presence of ^13^C-enriched pyruvate and alanine was observed, albeit the latter exhibited an even higher fraction, suggesting a conversion of ^13^C-labeled lactate and subsequent intracellular flow in these cells. In contrast to citrate fraction, there seemed to be relatively less accumulation of several other ^13^C-enriched TCA intermediates and derivatives, indicating the mitochondrial turnover of ^13^C-carbons through regular TCA cycling (Extended Data Fig. 6c).

Monocarboxylate transporters (MCTs) play a major role in intercellular lactate/H^+^ traffic and pH homeostasis regulation. Among diverse MCT isoforms, MCT1 and MCT4 functionally maintain an appropriate environmental acidity through lactate transport, with their high expression associated with cancer aggressiveness and poor prognosis^33^. We noticed upregulation of MCT1 (PC3) and MCT4 (MDA-MB-231) upon exposure of cells to exogenous lactate (Extended Data Fig. 6d,e). Of note, treatment with syrosingopine, a dual inhibitor of MCT1 and MCT4 (ref. ^34^), caused a significant decrease (>50%) of each of the aforementioned metabolites (Extended Data Fig. 6a,b), supporting the critical role of MCT1 or MCT4 in mediating the transport of exogenous lactate. Former studies indicated that cancer cells utilize stromal cell-derived and energy-rich metabolites in the mitochondrial TCA cycle to promote ATP production via OXPHOS and achieve enhanced malignancy, a phenomenon termed ‘reverse Warburg effect’^35^. To further establish the importance of MCT1 and MCT4 in mediating uptake of lactate from extracellular space, a process that allows development of the ‘reverse Warburg effect’, we measured energy production and proliferative capacity of cancer cells exposed to exogenous lactate and/or syrosingopine. Markedly reduced ATP production and proliferation of cancer cells in the presence of syrosingopine was observed, substantiating the pivotal role of MCT1/MCT4 in supporting such an malignancy-promoting event (Extended Data Fig. 6f,g); however, the detailed mechanism underlying cancer cell responses upon exposure to exogenous lactate remains to be elucidated.

### Therapeutically targeting PDK4 improves preclinical efficacy

**Extended Data Fig. 7:**
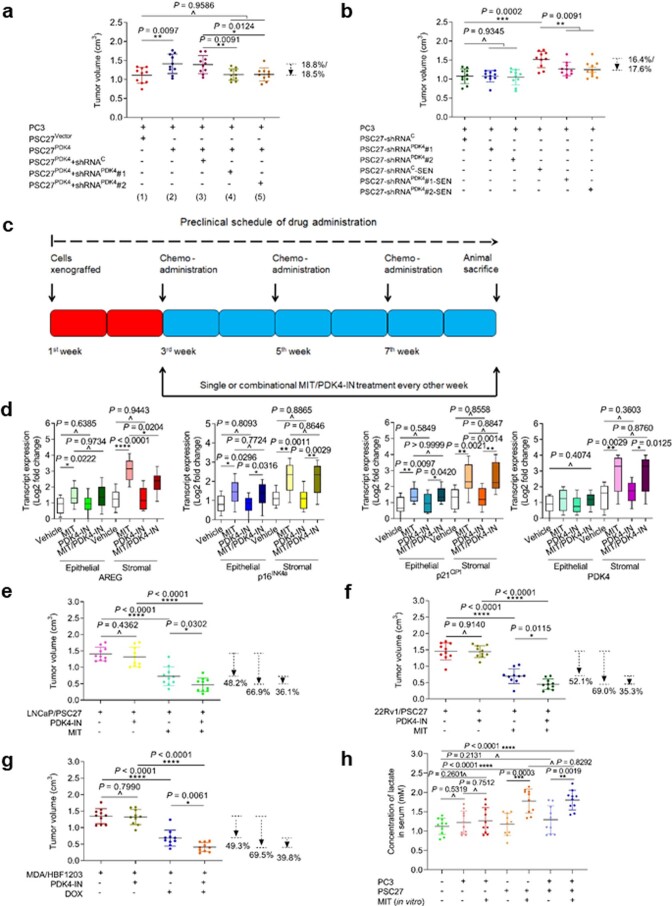
Schematic design of preclinical trial and comparative gene expression analysis. **a**. Statistics of tumor volumes in mice carrying cancer cells (PC3) and stromal cells (PSC27) as indicated. Tumor volumes measured at end of an 8-week period. **b**. Statistics of tumor volumes before animal exposure to chemotherapeutic agent mitoxantrone for senescence induction. **c**. Experimental workflow. Human cells inoculated subcutaneously to NOD/SCID males 2 weeks before chemotherapy. Agents delivered on 1 st day of each week starting from 3 rd week, then given every other week, totally 3 doses. Animals sacrificed 8 weeks later, tumors measured and tissues assessed. **d**. Transcript expression profiling of indicated factors. Individual cell types isolated from tumor tissues via LCM. Data representative of 3 biological replicates (n = 10 animals *per* group). **e**. Statistics of tumor volumes. LNCaP cells xenografted together with PSC27 to the hind flank, with MIT and/or PDK4 inhibitor PDK4-IN delivered via intravenous injection in a manner resembling PC3/PSC27 regimen. **f**. Statistics of tumor volumes. 22Rv1 cells were xenografted together with PSC27 to the hind flank, with MIT and/or PDK4-IN delivered via intravenous injection in a manner resembling PC3/PSC27 regimen. **g**. Statistics of tumor volumes. MDA-MB-231 (MDA) cells were xenografted together with HBF1203 to the hind flank, with DOX and/or PDK4-IN delivered via intravenous injection in a manner resembling PC3/PSC27 regimen. **h**. Statistics of circulating lactate concentration in serum. Peripheral blood of mice subject to lactate measurement at the end of regimen. PC3 and/or PSC27 cells were exposed to MIT in culture for senescence induction before implantation. Data in **a, b, d**-**h** are shown as mean ± S.D. and represent 3 biological replicates. For box and whiskers graphs (**d**), the minima, maxima, median, 25th and 75th percentiles are shown, with whiskers indicating smallest and largest values. For each dataset, *n* = 10 *per* treatment arm. *P* values were calculated by two-sided unpaired Student’s *t*-tests (**a**-**b**, **d**-**h**) or one-way ANOVA (**a**-**b**). MIT, mitoxantrone. DOX, doxorubicin. ^, *P* > 0.05. *, *P* < 0.05. **, *P* < 0.01. ***, *P* < 0.001. ****, *P* < 0.0001. Source data

Given the lactate-enriched microenvironment formed by PDK4-expressing stromal cells and its effects on cancer cell expression and phenotypes in vitro, we queried the pathological consequences of PDK4 induction in vivo. To this end, we constructed tissue recombinants by admixing PSC27 sublines with PC3 cells at a pre-optimized ratio of 1:4 before subcutaneous implantation to hind flank of experimental mice with severe combined immunodeficiency (SCID). Animals were gauged for tumor size at end of an 8-week period. Compared to tumors consisting of PC3 and PSC27^Vector^, xenografts consisting of of PC3 and PSC27^PDK4^ displayed significantly increased sizes (*P* < 0.01) (Extended Data Fig. 7a). Conversely, PDK4 knockdown by shRNA from these PSC27^PDK4^ cells before xenograft implantation markedly reduced tumor volumes (*P* < 0.01 and *P* < 0.05, respectively). We then depleted PDK4 from PSC27 before inducing senescence in vitro, and admixed with PC3 to generate tissue recombinants for xenografting. The data showed that the presence of senescent stromal cells markedly accelerated tumor growth, a tendency albeit significantly retarded upon PDK4 knockdown from stromal cells (Extended Data Fig. 7b). Thus, stromal PDK4 expression in senescent cells represents an important force driving tumor progression in vivo.

**Fig. 5:**
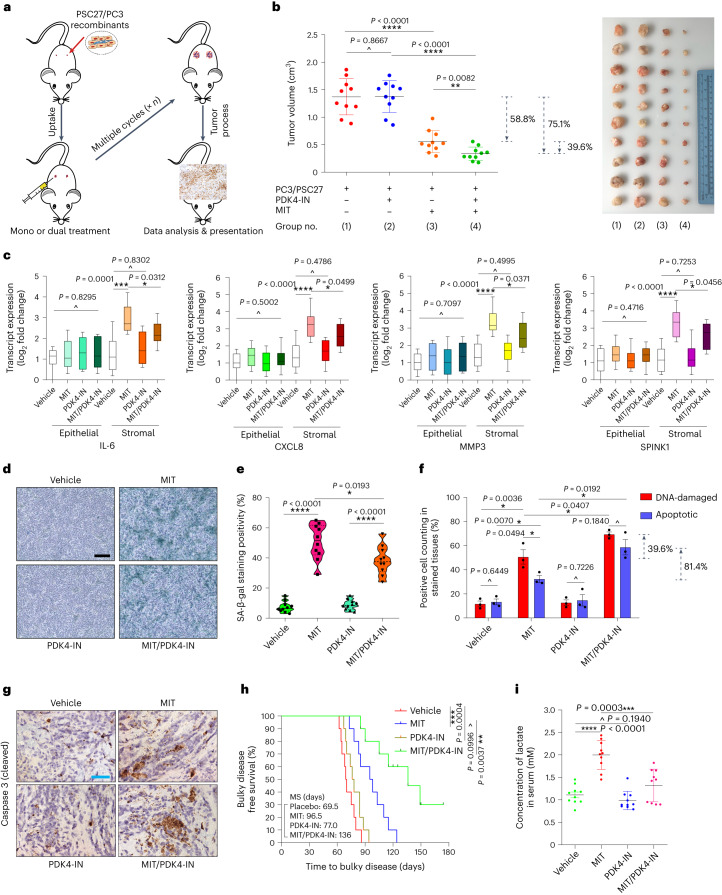
Therapeutically targeting PDK4 promotes anticancer outcome. **a**, Schematic workflow of experimental procedure. Two weeks after subcutaneous implantation and tissue recombinant uptake, animals received metronomic treatments. **b**, Statistical profiling of tumor end volumes. PC3 xenografted alone or together with PSC27 to the hind flank of animals. MIT and PDK4-IN administered either alone or concurrently to induce tumor regression. Representative tumor images (right). **c**, Transcript assessment of canonical SASP factors in stromal cells isolated from tumors. Tissues from animals subject to LCM isolation, total RNA preparation and expression assays. The group measured as of the lowest value was used as normalization baseline per factor. **d**, Representative IHC images of SA-β-gal staining profile of tissues isolated from placebo or drug-treated animals. Scale bar, 100 μm. **e**, Comparative statistics of SA-β-gal staining for mouse tissues described in **d**. **f**, Statistical assessment of DNA-damaged and apoptotic cells in tumor specimens analyzed in **d**. Values are presented as a percentage of cells positively stained by IHC with antibodies against γ-H2AX or caspase 3 (cleaved). **g**, Representative IHC images of caspase 3 (cleaved) in tumors at the end of therapeutic regimens. Biopsies of placebo-treated animals served as negative controls for drug-treated mice. Scale bars, 100 μm. **h**, Bulky DFS plotted against the time of implantation until animal death attributed to advanced bulky disease development. MS, median survival. *P* values calculated by two-sided log-rank (Mantel–Cox) tests. **i**, Measurement of circulating lactate in peripheral blood of mice that underwent therapeutic regimen involving MIT and/or PDK4-IN. Data in all dot, bar or violin graphs are shown as mean ± s.d. For animal assays, *n* = 10 (**b**,**c**,**e**,**h**,**i**) and *n* = 3 (**f**). For the box-and whiskers-graphs (**c**), minima, maxima, median, 25th and 75th percentiles are shown, with whiskers indicating smallest and largest values. *P* values were calculated by two-sided unpaired Student’s *t*-tests (**b**,**c**,**e**,**f**,**i**) or log-rank (Mantel–Cox) tests (**h**). MIT, mitoxantrone. ^*P* > 0.05; *P < 0.05; **P < 0.01; ***P < 0.001; ****P < 0.0001. Source data

To closely mimic clinical conditions involving chemotherapeutic agents, we designed a preclinical regimen incorporating a genotoxic drug (MIT) and/or the PDK4 inhibitor (PDK4-IN) (Fig. 5a). Two weeks after cell implantation when stable uptake of tumors by host animals was generally observed, a single dose of MIT or placebo was administered at the first day of the third, fifth and seventh week until end of the 8-week regimen (Extended Data Fig. 7c). Although PDK4-IN administration did not provide noticeable benefits, MIT treatment caused notable tumor shrinkage (58.8% volume reduction), validating the efficacy of MIT as a cytotoxic agent (Fig. 5b and Supplementary Fig. 4a). When PDK4-IN was combined with MIT, a further decline of tumor volume was observed (39.6%), resulting in a total shrinkage by 75.1% compared to the vehicle.

There was a considerable upregulation of typical SASP factors such as IL-6, CXCL8, MMP3, SPINK1 and AREG, accompanied by expression of typical senescence markers p16^INK4a^ and p21^CIP1^ in stromal cells of PC3/PSC27 xenografts, implying development of in vivo senescence and SASP expression upon MIT treatment (Fig. 5c and Extended Data Fig. 7d). Although PDK4-IN alone neither induced nor affected cellular senescence, it restrained the expression of hallmark SASP factors in the MIT-treated group (Fig. 5c and Extended Data Fig. 7d). Although senescence was induced in cancer cells in animals undergoing MIT treatment, as suggested by p16^INK4a^ and p21^CIP1^ expression, we did not observe a typical and full-spectrum SASP in these epithelial cells, largely consistent with our former findings^36,37^. Of note, PDK4 expression was induced in stromal cell populations, but not in their epithelial counterparts (Extended Data Fig. 7d), basically in line with in vitro datasets (Fig. 1d,e). Histology indicated elevated SA-β-gal positivity in tumor tissues of mice that experienced MIT treatment, but exposure to PDK4-IN resulted in a lower SA-β-gal positivity, suggesting that PDK4 likely contributes to cellular senescence in animals undergoing chemotherapy (Fig. 5d,e). These data indicate the operation of a mechanism allowing PDK4 to promote senescence, although this agent presumably neither targets DNA nor damages other macromolecules. Upon transduction of PDK4 into PCa lines, we observed significantly enhanced expression of a subset, albeit not all SASP factors, as well as senescence markers p16^INK4a^ and p21^CIP1^ in cells overexpressing PDK4 and treated by MIT (compared to cells transduced with vector and damaged by MIT; Supplementary Fig. 5a–c). The data further implied the potential role of PDK4 as a contributing factor for senescence and the SASP, although cancer cells displayed a SASP induction pattern somehow distinct from their normal stromal counterparts.

We next asked how pharmacologically targeting PDK4 could enhance the therapeutic response of tumors. To disclose the possible mechanism(s), we chose to dissect tumors from animals 7 d after initiation of treatment, a timepoint right before the development of resistant colonies. In contrast to the vehicle, MIT per se caused substantial DNA damage and apoptosis in cancer cells (Fig. 5f). Although PDK4-IN alone neither caused typical DNA damage response (DDR) nor induced cell apoptosis, it showed prominent efficacy in enhancing these therapeutic indices upon combination with MIT (*P* < 0.05). IHC staining disclosed increased caspase 3 cleavage, a canonical apoptosis indicator, upon MIT administration, with the tendency further enhanced by PDK4-IN (Fig. 5g).

To expand, we used LNCaP, a second PCa cell line that expresses an androgen receptor (AR) and is routinely employed as a hormone-responsive cell model. To produce an AR-naive setting, we circumvented experimental castration, but followed the same protocol designed for PC3-tailored regimens. We noticed significantly reduced volumes of LNCaP/PSC27 tumors when mice underwent MIT/PDK4-IN co-treatment, in contrast to MIT administration only (36.1%) (Extended Data Fig. 7e). Similar results were observed when 22Rv1, a castration-resistant PCa cell line, was applied to replace LNCaP for in vivo assays (35.3%) (Extended Data Fig. 7f). We further generated xenografts composed of MDA-MB-231 and HBF1203, the latter a breast stromal cell line. The BCa tumor-associated results largely produced those observed in PCa tumors (39.8%) (Extended Data Fig. 7g). Together, these data suggest that targeting of PDK4, specifically in a treatment-damaged TME, which harbors a considerable number of senescent cells, can substantially promote tumor regression in chemotherapeutic settings, a process independent of androgen regulation or AR signaling of prostate tumors per se. We hereby conclude that the resistance-minimizing effects of PDK4-targeting strategy are not limited to a specific cancer type, but may have implications to a wide range of malignancies.

We next assessed tumor progression consequence by comparing the survival of different animal groups in a time-extended preclinical cohort, with PCa mice as a pilot model. During tumor surveillance, bulky disease was considered once the tumor burden became prominent (size ≥2,000 mm^3^), an approach described previously^15,38^. Mice receiving MIT/PDK4-IN combinational treatment displayed the most prolonged median survival, gaining a 40.9% longer survival compared to those treated by MIT only (Fig. 5h; green versus blue); however, PDK4-IN treatment alone did not achieve significant benefits, as it conferred only marginal survival advantage (Fig. 5h; brown versus red). Thus, targeting PDK4 alone affects neither tumor growth nor animal survival, whereas MIT/PDK4-IN co-treatment has the competence to significantly improve both parameters.

Upon measurement of circulating levels of lactate in serum, we noticed a markedly elevated lactate concentration in animals treated by MIT, but not PDK4-IN (Fig. 5i); however, lactate level seemed markedly reduced upon application of PDK4-IN together with MIT in contrast to the MIT-only group. More notably, in vivo data indicated that mouse lactate level increased only in the presence of senescent stromal cells, rather than their control counterparts or cancer cells (regardless of proliferating or senescent) (Extended Data Fig. 7h). Therefore, senescent stromal cells represent a major source of lactate production in treatment-damaged microenvironment, an index technically measurable in peripheral blood.

Data from safety appraisal supported that either single or combinatorial treatment was well tolerated, as evidenced by body weight maintenance throughout the therapeutic timeframe (Supplementary Fig. 6a). There were no significant perturbations in the serum level of creatinine, urea and metabolic activities of liver enzymes (alkaline phosphatase and alanine transaminase (ALT)) (Supplementary Fig. 6b). Data from mice developing BCa carcinomas and treated by doxorubicin (DOX)/PDK4-IN generally phenocopied those in animals with PCa (Supplementary Fig. 6c,d). Therapeutic safety was further demonstrated by MIT/PDK4-IN-treated and DOX/PDK4-IN-treated immunocompetent animals (C57BL/6J), which manifested no routine blood count fluctuations, thus essentially validating the feasibility of these regimens (Supplementary Fig. 7a–f). Thus, strategies combining a PDK4-targeting agent with classical chemotherapy hold the potential to enhance tumor responses without causing severe systemic cytotoxicity.

### Serum lactate adversely predicts survival of patients with cancer

**Fig. 6:**
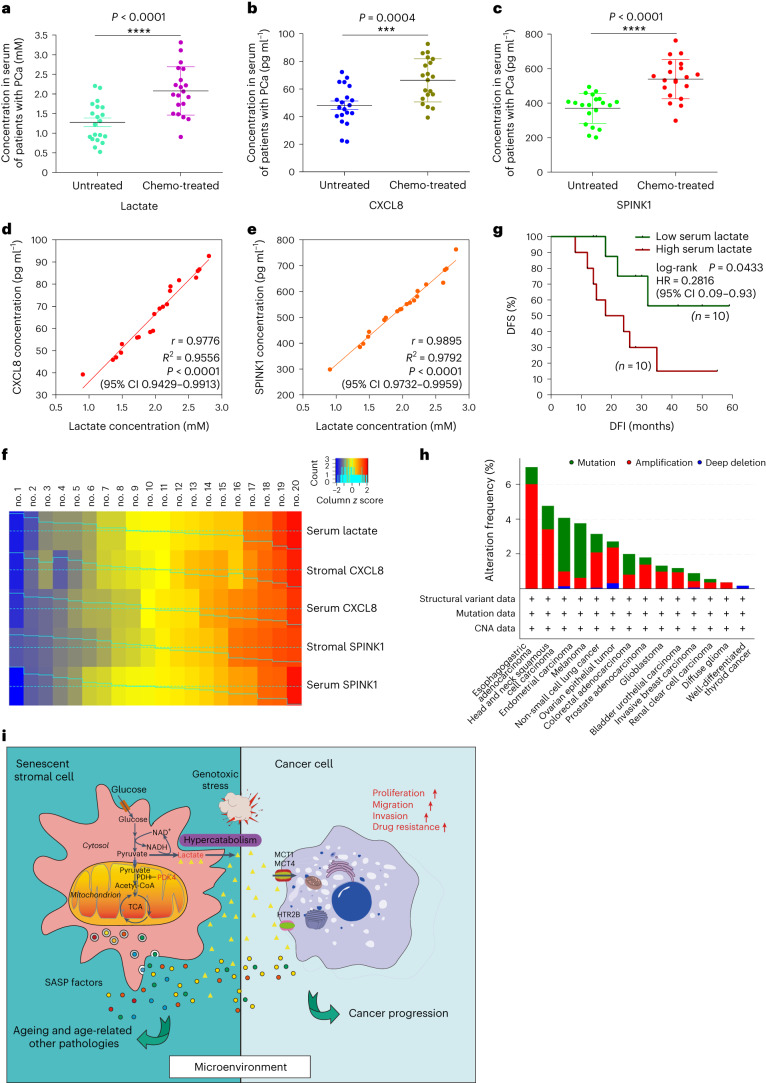
Lactate indicates in vivo SASP development and predicts adverse clinical outcome. **a**, Abundance of lactate in serum of untreated and chemo-treated patients with PCa. Data are derived from ELISA and shown as mean ± s.d. *n* = 20. **b**, Abundance of CXCL8 protein in patient serum analyzed in **a**. Data are from an ELISA and are presented as mean ± s.d.; *n* = 20. **c**, Abundance of SPINK1 protein in patient serum analyzed in **a**. Data are from an ELISA and are presented as mean ± s.d.; *n* = 20. **d**, Scatter-plot showing correlation between lactate and CXCL8 in the serum of individual patients described in **a**–**c**. Pearson’s correlation coefficient, *P* value and confidence interval indicated. **e**, Scatter-plot showing correlation between lactate and SPINK1 in the serum. Pearson’s correlation coefficient, *P* value and confidence interval indicated. **f**, Heat map depicting overall correlation between serum lactate, stromal/serum CXCL8, stromal/serum SPINK1 in chemo-treated patients (*n* = 10). Raw scores of stromal factors from independent pathological reading of primary tumors, with those of serum factors from ELISA. Color key, relative expression. **g**, Kaplan–Meier survival analysis of chemo-treated patients with PCa. DFS stratified according to circulating lactate in serum (low, average score <2, dark green; high, average score ≥2, dark red). DFS represents length (months) of period calculated from the date of chemotherapy to point of first time disease relapse. Survival curves generated according to the Kaplan–Meier method, with *a P* value calculated using a log-rank (Mantel–Cox) test; *n* = 10 per group. DFI, disease-free interval; HR, hazard ratio. **h**, TCGA data showing alterations of PDK4 in a variety of human cancer types at genomic level, including mutation, amplification and deep deletion. Alteration frequency displayed in percentage. **i**, Graphic illustration to summarize metabolic reprogramming of senescent cells and formation of lactate-enriched microenvironment in a genotoxic setting and functional implications of the metabolite lactate in promoting cancer resistance and potentially other age-related conditions. Data in **a**–**c** are shown as mean ± s.d. *P* values were calculated by two-sided unpaired Student’s *t*-tests (**a**–**c**), Pearson correlation tests (**d**,**e**) or log-rank (Mantel–Cox) tests (**g**). ***P < 0.001; ****P < 0.0001. Source data

Despite the correlation of higher PDK4 expression in tumor stroma with lower post-treatment survival (Extended Data Fig. 2g and Supplementary Fig. 1d), whether the metabolite lactate derived from stromal cells developing TIS is technically detectable and whether it can serve as a clinical marker, remains unclear. We acquired peripheral blood samples from patients with PCa, including one cohort that experienced standard neoadjuvant chemotherapy and the other that did not. ELISA assays of serum from chemo-treated patients revealed lactate levels in the treated cohort significantly higher than that of treatment-naive group (Fig. 6a). The pattern was reproduced by a remarkable increase of CXCL8 and SPINK1, canonical SASP hallmarks, in the same cohort of post-treatment patients (Fig. 6b,c). These data suggest that a circulating scale of lactate emerges in the peripheral blood alongside an in vivo SASP, and both are systemically traceable in the serum of treated patients with cancer. More notably, subsequent analysis of ELISA data disclosed a significant and positive correlation between lactate and CXCL8, as well as between lactate and SPINK1 (Fig. 6d,e). Thus, lactate production and SASP expression is mutually linked, largely resembling the correlation between PDK4 induction and SASP development as revealed by data derived from tumors per se (Extended Data Fig. 2f).

As further investigations continue, we performed longitudinal analysis in both primary tumor foci and peripheral blood (20 chemo-treated patients randomly selected). Notably, cross-organ comparisons indicated a pronounced association between in-tissue expression and circulating level per factor, with lactate, CXCL8 and SPINK1 apparently varying in parallel either within the primary tissue or through peripheral blood (Fig. 6f). Altogether, lactate represents a TME-derived biological factor precisely mirroring development of an in vivo SASP and can be exploited to evaluate SASP magnitude in post-treatment patients with cancer.

Clinical profiling subsequently uncovered a negative correlation between plasma level of lactate and post-treatment survival (Fig. 6g). As PDK4 is subject to frequent mutation, amplification and deep deletion as disclosed by TCGA pan-cancer atlas studies (querying 22,179 patients and 22,802 samples in 36 clinical studies)^39,40^ (Fig. 6h), the molecule represents an important predictor of disease progression in treatment-naive patients in clinical oncology^41,42^. Contrasting with previous studies focusing on genomic alterations and pathological behaviors of cancer cells, we herein propose that routine surveillance of lactate, a major metabolic product derived from PDK4-driven glycolysis in stromal cells upon TIS, via a noninvasive avenue such as liquid biopsy, provides a new, practical and accurate strategy for appraisal of advanced pathologies in clinical oncology (Fig. 6i).

### Lactate activates ROS production via NOX1 in senescent cells

**Fig. 7:**
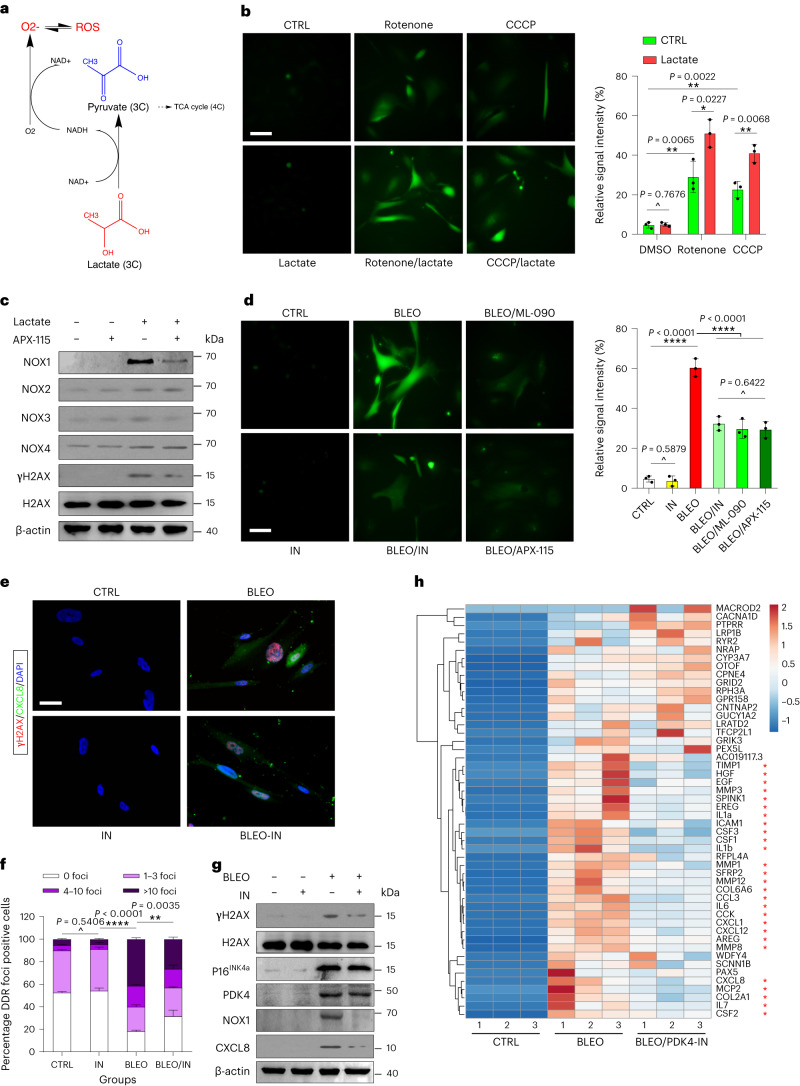
Lactate activates ROS production via NOX1 and enhances SASP intensity. **a**, Biochemical scheme illustrating intracellular mechanisms of ROS generation upon exposure of cells to lactate, a small molecule metabolite derived from either autocrine or paracrine pathways in-tissue microenvironment. **b**, Examination of ROS biogenesis with DCFH_2_-DA, a cell-permeable fluorescent probe sensitive to changes in cellular redox state. Experiments performed 1 d after treatment of PSC27 cells with rotenone (10 μM), CCCP (10 μM) and/or lactate (10 mM). Representative images (left). Scale bar, 10 μm. Statistics (right). DMSO, dimethylsulfoxide. **c**, Immunoblot assay of representative NOX molecules and DDR signaling after exposure of cells to different treatments. β-actin, loading control. **d**, Measurement of ROS production with DCFH-DA. Experiments performed 1 d after treatment of BLEO-induced senescent PSC27 cells with ML-090, PDK4-IN and APX-115. Representative images (left). Scale bar, 10 μm. Statistics (right). **e**, Confocal microscopy of immunofluorescence staining of PSC27 cells treated by BLEO and/or PDK4-IN. Primary antibodies against γH2AX and CXCL8 employed (red and green, respectively, after secondary antibody incubation and laser excitation; blue, 4,6-diamidino-2-phenylindole (DAPI)). Scale bar, 10 μm. **f**, Comparative statistics of DDR in PSC27 cells treated by agents as indicated in **e**. DDR was classified into four sub-categories including 0 foci, 1–3 foci, 4–10 foci and >10 foci per cell. **g**, Immunoblot analysis of the expression of target molecules after exposure of cells to different treatments. CXCL8, a hallmark SASP factor; β-actin, loading control. **h**, Heat map depicting expression change pattern of genes in the transcriptome-wide range. The first 50 genes most upregulated upon BLEO treatment are shown, with their changes in the presence of PDK4-IN lined up correspondingly. Red stars indicate representative SASP factors. The data in the bar graphs of **b** and **d** are shown as mean ± s.d. For datasets in **b** and **d**, *n* = 3. Data in **c**,**g** are representative of two independent experiments. *P* values were calculated by two-sided unpaired Student’s *t*-tests (**b**,**d**) or two-way ANOVA (**f**). ^*P* > 0.05; *P < 0.05; **P < 0.01; ***P < 0.001; ****P < 0.0001. Source data

Our data suggest that suppression of PDK4 activity partially affects senescence and the SASP (Fig. 5c–e and Extended Data Fig. 7d), but the underlying mechanism remains unclear. We next queried whether and how the elevated production of lactate, which can result from PDK4 upregulation in senescent cells, change their neighboring noncancerous counterparts. Lactate triggers ROS generation in mammalian cells via a mechanism involving oxidation of lactate to pyruvate by lactate dehydrogenase (LDH), a process accompanied by the transformation of NAD^+^ to NADH, whereas the latter can be further used by the NADPH oxidase (NOX) to generate ROS in a lactate-NOX-ROS axis (Fig. 7a)^43,44^. Our data suggest that treatment of normal stromal cells (PSC27) with lactate at an experimentally pre-optimized concentration (10 mM) failed to induce ROS elevation (Fig. 7b); however, lactate exposure of cells developing mitochondrial deficiency, which was caused by treatment with chemicals such as rotenone and carbonyl cyanide m-chlorophenylhydrazone (CCCP) (an electron transport chain complex I inhibitor and an OXPHOS uncoupler, respectively), resulted in further elevated ROS production, as evidenced by increased signals of 2′,7′-dichlorofluorescein (DCF), the latter derived from the fluorogenic probe 2′,7′-dichlorodihydrofluorescein diacetate (DCFH_2_-DA) (Fig. 7b).

**Extended Data Fig. 8:**
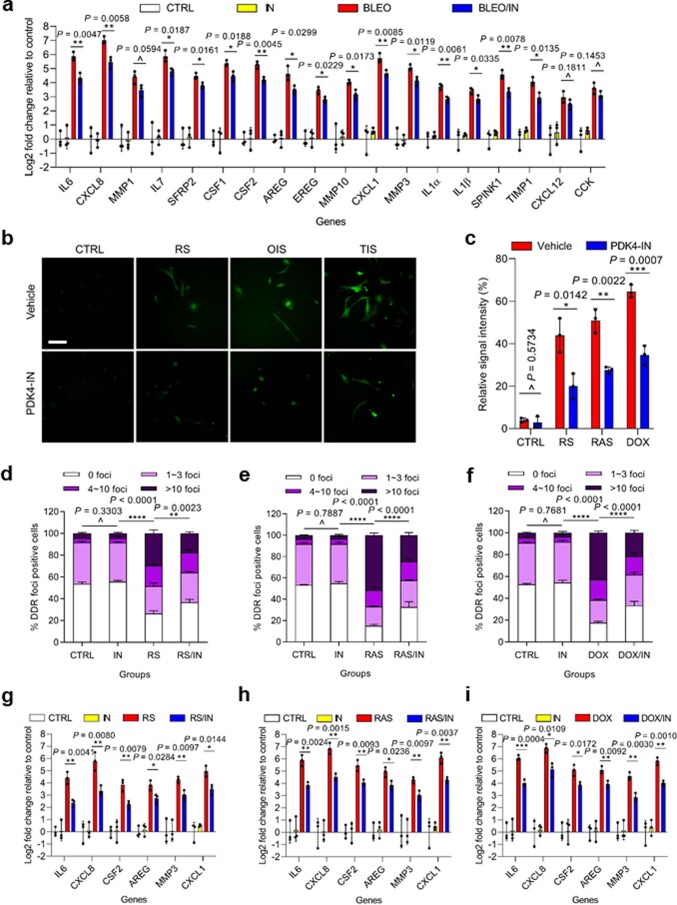
Impact of PDK4 targeting on SASP expression, ROS production and DNA damage intensity. **a**. Transcript-based quantitative examination of typical SASP factors expressed by PSC27 cells upon treatment with the genotoxic agent BLEO and/or PDK4 inhibitor PDK4-IN (IN). Data normalized to CTRL group *per* factor. **b**. Measurement of ROS levels with DCFH-DA, a cell-permeable fluorescent probe sensitive to alterations in cellular redox state. PSC27 cells allowed to reach replicative exhaustion (RS), engineered to overexpress *HRas*^*G12V*^ (OIS) or subject to genotoxic stress by doxorubicin (TIS). Scale bar, 10 μm. **c**. Relative quantification and statistical comparison of DCF signals in each sample as described in **b**. **d**. Comparative statistics of DDR intensity in PSC27 cells undergoing RS and/or treated by PDK4-IN. DDR classified into four sub-categories including 0 foci, 1-3 foci, 4-10 foci and >10 foci *per* cell. **e**. Comparative statistics of DDR intensity in PSC27 cells undergoing *HRas*^*G12V*^-induced OIS and/or treated by PDK4-IN. **f**. Comparative statistics of DDR intensity in PSC27 cells undergoing DOX-induced TIS and/or treated by PDK4-IN. **g**. Quantitative RT-PCR-based transcript assay of representative SASP factors expressed by PSC27 cells undergoing RS and/or upon treatment with PDK4-IN. **h**. Quantitative RT-PCR-based transcript assay of representative SASP factors expressed by PSC27 cells upon senescence induced by the oncogene *HRas*^*G12V*^ and/or treatment with PDK4-IN. **i**. Quantitative RT-PCR-based transcript assay of representative SASP factors expressed by PSC27 cells upon senescence induced by the genotoxic agent DOX and/or treatment with PDK4-IN. Data in all bar plots are shown as mean ± S.D. and represent 3 biological replicates. All *P* values were calculated by two-sided unpaired Student’s *t*-tests (**a**, **c**, **g**-**i**) or two-way ANOVA (**d**-**f**). ^, *P* > 0.05, *, *P* < 0.05. **, *P* < 0.01. ***, *P* < 0.001. ****, *P* < 0.0001. Source data

The NOX family consists of five homologs, NOX1 to NOX5, and two related enzymes, DUOX1 and DUOX2. We observed enhanced expression of NADPH oxidase 1 (NOX1), but not other homologs of the NOX family, after lactate treatment of senescent, but not proliferating PSC27 cells (Fig. 7c and Supplementary Fig. 8a). APX-115, a pan-NOX inhibitor, markedly reduced the expression of NOX1, but not other NOX molecules. We assessed potential treatment-caused effects on lactate production upon exposure of stromal cells to PDK4-IN, an experimental assay that allowed to examine the ROS biogenesis mechanism from the opposite side. Inhibition of PDK4 activity markedly diminished the capacity of genotoxicity-induced senescent cells in producing the ROS, a tendency largely reproduced by treatment with ML-090 or APX-115, the former a chemical inhibitor against NOX1 (Fig. 7d). As DDR events are typically responsible for the ROS generation in senescent cells, we questioned the possibility of lactate in promoting genotoxicity via ROS production. Immunofluorescence staining indicated reduced intensity of DDR foci in BLEO-damaged cells upon exposure to PDK4-IN, although the agent did not cause changes to proliferating cells (Fig. 7e,f). CXCL8, one of the hallmark SASP factors, exhibited reduced level upon PDK4 suppression, a pattern largely consistent with the vast majority of other SASP factors (Fig. 7g,h and Extended Data Fig. 8a). In contrast to signal intensities of NOX1 induction and DDR activation, the latter imaged by phosphorylated H2AX (γH2AX), expression of p16^INK4a^ and PDK4 seemed largely unaffected by PDK4-IN, suggesting differential regulatory mechanisms that modulate the expression or activation of these molecules (Fig. 7g). RNA-seq data indicated that a large array of genes were significantly upregulated upon exposure to genotoxicity, but substantially altered by PDK4-IN (Fig. 7h). Among these genes, many indeed encode secreted factors falling in the SASP spectrum. Therefore, PDK4-mediated lactate production enhances activation of NOX1, which potently drives ROS generation and promotes SASP development, a process accompanied by elevated DDR signaling, while inhibiting PDK4 activity or lactate production pathway can restrain senescence-associated phenotypes, particularly the SASP.

Cell damage can be triggered by multiple stressors, resulting in development of senescence as either RS, OIS or TIS. To substantiate experimentally the findings correlated with the implication of lactate, specifically upon production by senescent cells via the autocrine manner, in cell phenotypic development, we examined the effect of PDK4 suppression in the settings of RS, OIS and TIS (DOX-induced). Cell-based assays demonstrated that ROS production of senescent cells was generally minimized by PDK4-IN (Extended Data Fig. 8b,c), a mitochondria-associated activity largely consistent with alleviated DNA damage (Extended Data Fig. 8d–f). Expression of typical SASP factors was reduced by PDK4-IN, basically reproducing data in BLEO assays (Extended Data Fig. 8g–i). Thus, senescent cells hold the potential to engage the ROS–SASP axis upon uptake of lactate, which can be derived readily from the microenvironment.

Dysfunctional mitochondria are responsible for increased ROS production, whereas mitochondrial deficiency-intervened senescent cells display remarkably reduced mitochondrial ROS production^45^. We next reasoned whether lactate-promoted ROS generation is mediated by dysfunctional mitochondria in senescent cells, and whether targeting OXPHOS affects senescence-associated phenotypes, specifically the SASP. A PARKIN-mediated mitophagy model was employed to functionally remove disabled mitochondria, with PSC27 transduced with a control or *PRKN* construct followed by senescence induction. Alternatively, we applied Gboxin, an inhibitor of OXPHOS and suppressor of F_0_F_1_ ATP synthase activity, to treat cells before inducing senescence. Of note, both ROS production and SASP synthesis substantially declined in senescent cells upon PARKIN-mediated mitophagy (Supplementary Fig. 8b,c). Similarly, both ROS production and SASP expression were markedly weakened in the presence of Gboxin (Supplementary Fig. 8d,e). We observed reduced ATP production in Gboxin-treated senescent cells, although the SA-β-gal positivity and in culture viability remained largely unaffected (Supplementary Fig. 7f–i), implying that inhibition of mitochondria OXPHOS affects both SASP development and ATP production, which cannot be compensated by only enhanced glycolysis. The results suggest that lactate-induced ROS production is basically mediated through dysfunctional mitochondria in senescent cells, whereas functional removal of defective mitochondria or interference of mitochondrial OXPHOS abrogates the SASP, a senescence-featured hallmark that requires sustained energy supply through mitochondrial ATP production via intact OXPHOS.

### Targeting PDK4 alleviates physical dysfunction and extends lifespan

**Fig. 8:**
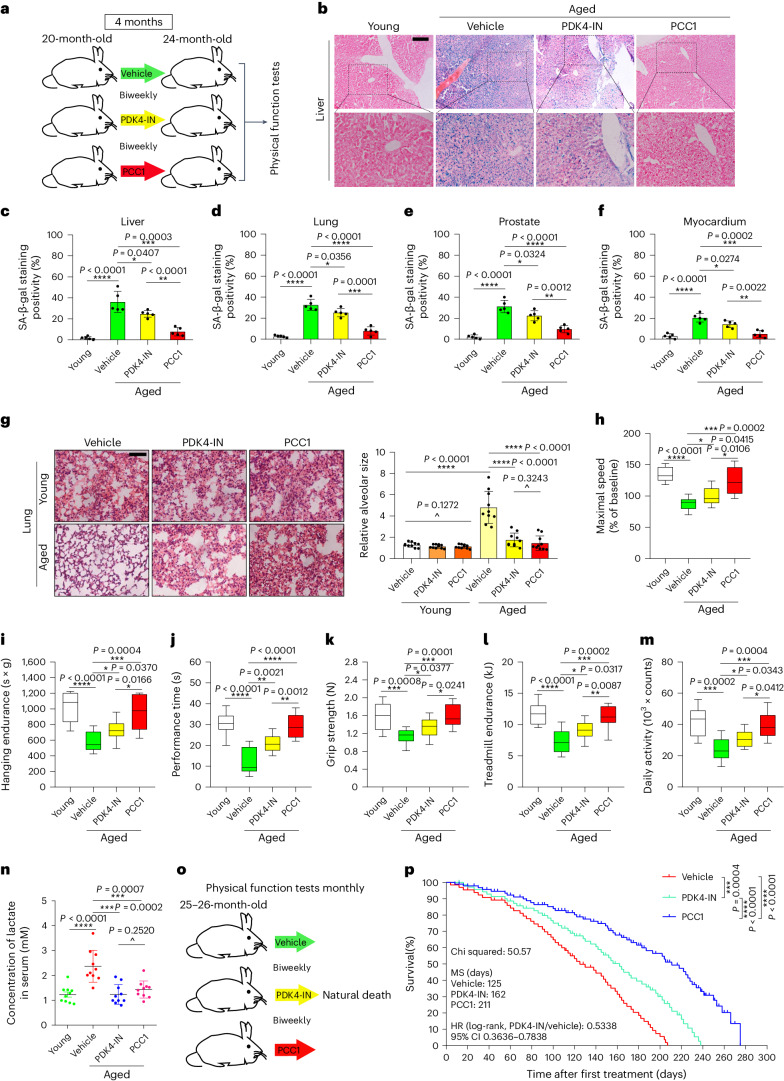
PDK4-targeting alleviates frailty and extends lifespan of aged animals. **a**, Schematic design of physical functional examinations for 20-month-old C57BL/6J mice receiving preclinical treatment by vehicle, PDK4-IN or PCC1 (biweekly) for consecutive 4 months. PCC1, senolytic control. **b**, Representative images of SA-β-gal staining of livers from young and aged mice treated with vehicle, PDK4-IN or PCC1 as described in **a**. Scale bar, 200 μm. **c**, Quantification and comparison of SA-β-gal staining positivity in liver tissues. **d**, Quantification and comparison of SA-β-gal staining positivity in lung tissues. **e**, Quantification and comparison of SA-β-gal staining positivity in prostate tissues. **f**, Quantification and comparison of SA-β-gal staining positivity in myocardium tissues. **g**, Representative hematoxylin and eosin (H&E) staining (left) and quantification of alveolar size (right). Scale bar, 200 μm. **h**, Quantitative measurement of maximal walking speed (relative to baseline) of experimental mice. **i**–**m**, Quantitative measurement of maximal walking speed (relative to baseline) (**i**), performance time (**j**), grip strength (**k**), treadmill endurance (**l**) and daily activity (**m**) of 20-month-old animals after the 4-month treatment. **n**, Measurement of circulating lactate (in mM) in the peripheral blood of mice after the 4-month treatment as described in **a**. **o**, Schematic design for lifespan appraisal of mice (both sexes) at 25–26 months of age. **p**, Post-treatment survival curves of C57BL/6J animals treated biweekly with vehicle (*n* = 58; 31 males and 27 females), PDK4-IN (*n* = 55; 28 males and 27 females) or PCC1 (*n* = 51; 26 males and 25 females) starting at 25–26 months of age. Animals in each group were adapted in three (young) or four (aged) independent cages. For preclinical assays, *n* = 5 per group (**c**–**f**) and *n* = 10 per group (**g**–**n**). Data in all bar and dot graphs are shown as mean ± s.d. (**c**–**g**,**n**). For box-and-whisker graphs (**h**–**m**), the minima, maxima, median, 25th and 75th percentiles are shown, with whiskers indicating smallest and largest values. *P* values were calculated by two-sided unpaired Student’s *t*-tests (**c**–**f**,**g**–**n**) or log-rank (Mantel–Cox) tests (**p**). ^*P* > 0.05; *P < 0.05; **P < 0.01; ***P < 0.001; ****P < 0.0001. Source data

Given the pharmacological development of a series of geroprotective agents, particularly those targeting senescent cells, we queried whether administration of PDK4-specific agents such as PDK4-IN can postpone chronological aging and/or restrain age-related phenotypes. In this study, we chose to treat normal 20-month-old wild-type (WT) mice with vehicle or PDK4-IN (10 mg kg^−1^ via intraperitoneal (i.p.) injection) (once every 2 weeks) for 4 months, after which physical function was experimentally determined (Fig. 8a). Histological evaluation disclosed emerging senescent cells in solid organs, as reflected by elevated SA-β-gal positivity in liver, lung, prostate and myocardial tissues of aged animals, changes that were partially but significantly reversed by PDK4-IN (Fig. 8b–f); however, the efficacy of PDK4-IN in depleting senescent cells seemed to be generally lower than that of PCC1, a natural senolytic agent that can selectively eradicate senescent cell populations in vivo as we recently discovered^46^. Although SA-β-gal activity is not always associated with cellular senescence, particularly in the case of cellular quiescence and macrophage infiltration within the tissue microenvironment^47,48^, overall tendency of reduced SA-β-gal positivity in these organs supports therapeutic effectiveness of PDK4-IN, an agent herein used to target senescent cells in aged animals.

**Extended Data Fig. 9:**
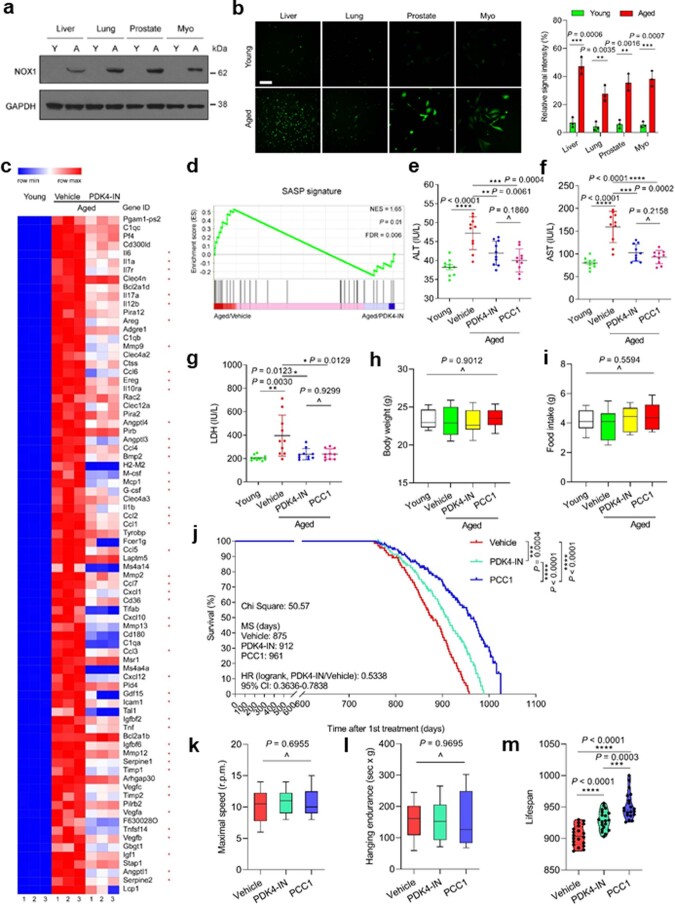
Preclinical profiling of ROS generation, liver physiological index, genome-wide expression and physical function of aged mice. **a**. Immunoblot analysis of NOX1 expression across organ types in young *vs* aged mice. Y, young. A, aged. Myo, myocardium. GAPDH, loading control. **b**. Measurement of ROS levels with DCFH-DA. Left, representative images. Right, statistical comparison. Scale bar, 20 μm. **c**. Heat map depicting expression pattern of genes significantly upregulated upon BLEO-induced senescence but subject to counteraction by PDK4-IN. Red stars, representative SASP factors. **d**. GSEA plot of a significant gene set in SASP spectrum. FDR, false discovery rate; NES, normalized enrichment score. **e**. The serum levels of alanine transaminase (ALT) from young mice and their aged counterparts that received biweekly treatment as indicated at 20 months of age for 4 consecutive months. **f**. The serum levels of aspartate transaminase (AST). **g**. The serum levels of lactate dehydrogenase (LDH). **h**. Measurement of body weights. **i**. Assessment of daily food intake. **j**. Whole-life survival curves of C57BL/6 J mice treated biweekly with vehicle (n = 68; 37 males, 31 females), PDK4-IN (n = 66; 33 males, 33 females) or PCC1 (n = 67; 35 males, 32 females) starting at 24-27 months of age. **k-l**. Maximal walking speed (**k**) and hanging endurance (**l**) averaged over the last 2 months of life. **m**. Appraisal of lifespan for the longest living mice (top 20) *per* group. Animals were adapted in 3 (young) or 4 (aged) independent cages. For in vitro experiments, *n* = 3 for **b**. For preclinical assays, *n* = 10/group for **e**-**i** and **k**-**m**. Data in **b, e**-**i** and **k**-**m** are shown as mean ± S.D. For box and whiskers graphs (**h**-**i, k**-**l**), the minima, maxima, median, 25th and 75th percentiles are shown, with whiskers indicating smallest and largest values. *P* values were calculated by two-sided unpaired Student’s *t*-tests (**b**, **e**, **f**, **g**, **m**), one-way ANOVA (**h**-**i**, **k**-**l**) or Log-rank (Mantel–Cox) tests (**j**). For data in **d**, *P* value was calculated by one-way ANOVA with Tukey’s post hoc comparison. ^, *P* > 0.05. *, *P* < 0.05. **, *P* < 0.01. ***, *P* < 0.001. ****, *P* < 0.0001. Source data

Age-dependent increase of alveolar volume, decrease of maximal speed, hanging endurance, beam balance performance, grip strength and motor skills, and reduction of daily activity were substantially improved in mice receiving administration of PDK4-IN in aged (20-month-old) mice compared to the vehicle group (Fig. 8g–m). Although most geroprotective effects generated by PDK4-IN seemed to be largely inferior to PCC1 group, the efficacy of these two agents still resembling each other in some cases. For instance, age-dependent expansion of the pulmonary alveolus, a pathological change contributing to pulmonary dysfunction, was well controlled by both agents (Fig. 8g). The data suggest a critical role of targeting PDK4 in restraining age-related pulmonary abnormality, as changes in the composition of the airways and the alveoli may result in reduced respiratory function and eventually lead to chronic lung disorders during mammalian aging. We observed upregulation of NOX1 in multiple tissues of aged animals, which was basically in line with elevated ROS levels in vivo (Extended Data Fig. 9a,b). The SASP expression was considerably restrained in tissues such as lungs (alveolar cells) of aged mice treated with PDK4-IN compared to the vehicle group (Extended Data Fig. 9c,d), which is largely consistent with in vitro data (Fig. 7h).

Concentration of circulating lactate in the serum was substantially reduced in aged animals receiving PDK4-IN treatment, but without significant difference from those that were PCC1-treated, implying a notable contribution of PDK4 in mediating lactate production (Fig. 8n). Cellular senescence plays a key role in pathogenesis of nonalcoholic steatohepatitis (NASH) by promoting hepatic fat accumulation and steatosis during aging^49,50^. To dissect the potential of NASH development associated with natural aging and to address the feasibility of controlling this pathology via targeting senescent cells, we examined a subgroup of mice at 20 months of age, and administered them with a vehicle, PDK4-IN or PCC1 for 4 months. A number of aged mice exhibited an increased tendency to develop liver dysfunction, as indicated by elevated serum levels of ALT, aspartate transaminase (AST) and lactate dehydrogenase (LDH) (Extended Data Fig. 9e–g); however, administration of PDK4-IN notably prevented these changes, suggesting that PDK4 inhibition ameliorated liver pathogenesis. In contrast, body weight and food intake levels remained largely unaffected in aged mice (Extended Data Fig. 9h,i), suggesting overall safety of the PDK4-targeting regimen in aged mice.

To establish the potential of limiting lactate production by senescent cells to prolong the remaining lifespan of very aged animals, we performed PDK4 treatment beginning at an advanced stage (Fig. 8o). Mice receiving PDK4-IN administration (biweekly) starting at 25–26 months of age (largely equivalent to an age of 80–85 years in humans) had a 29.6% longer median post-treatment lifespan (or 4.2% longer overall lifespan) and lower mortality (53.4%, *P* < 0.001) than vehicle-treated (Fig. 8p and Extended Data Fig. 9j) mice. These data suggest that PDK4-specific intervention significantly reduces the risk of age-related mortality in aged mice.

**Extended Data Fig. 10:**
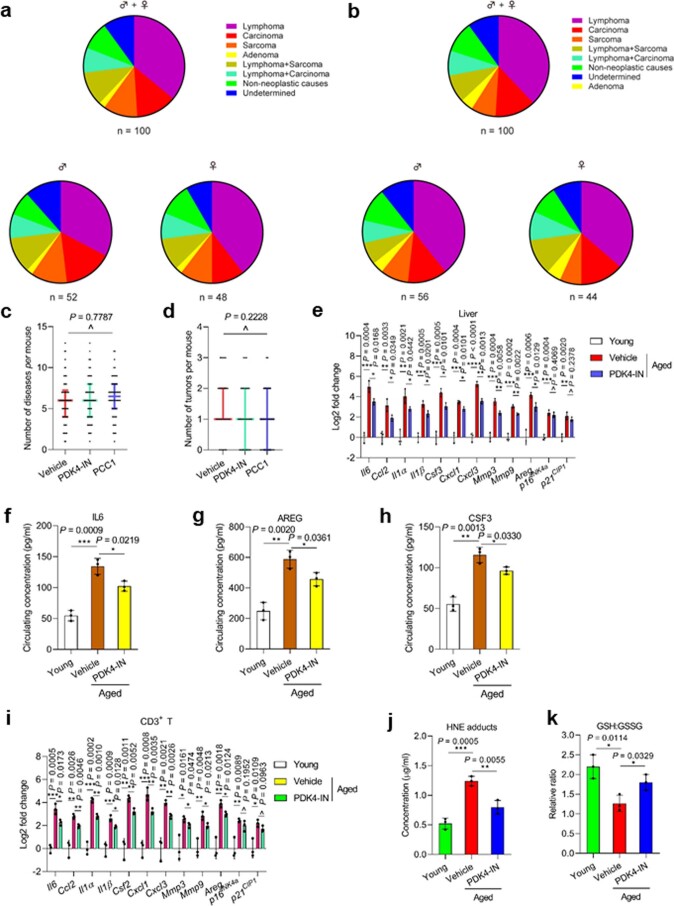
Late-life PDK4 targeting does not change death causes but restrains SASP and minimizes oxidative stress. **a**-**b**. Pie charts profiling the ultimate causes of death of C57BL/6 J mice (males and females) that received vehicle (**a**) or PDK4-IN (**b**) biweekly treatment starting from 25–26 months of age. Note, there was no significant difference between vehicle- and PDK4-IN-treated groups upon analysis using either Chi-square or Fisher’s exact tests. **c**. Overall disease burden of mice at death. For both sexes, *n* = 50 *per* arm. For males (♂), *n* = 26 for vehicle, *n* = 25 for PDK4-IN and *n* = 26 for PCC1. For females (♀), *n* = 24 for vehicle, *n* = 25 for PDK4-IN and *n* = 24 for PCC1. **d**. Tumor burden of animals at death. For both sexes, n = 30 *per* arm. For males (♂), *n* = 15 for vehicle, *n* = 16 for PDK4-IN and *n* = 17 for PCC1. For females (♀), *n* = 15 for vehicle, *n* = 14 for PDK4-IN and *n* = 13 for PCC1. **e**. qRT-PCR profiling of the SASP and senescence marker expression in liver tissues of young (6-month-old, untreated), aged (25–26-month-old) vehicle-treated and aged (25–26-month-old) PDK4-IN-treated animals, respectively. **f**-**h**. Measurement of circulating levels of hallmark SASP factors IL6 (**f**), AREG (**g**) and CSF3 (**h**) in mouse serum by ELISA assays. **i**. Quantitative measurement of the SASP and senescence marker expression in CD3^+^ peripheral T cells of experimental mice described in **e**-**h**. **j**. Assessment of 4-hydroxynonenal (HNE) adducts, a marker of lipid peroxidation and oxidative stress by ELISA measurement with tissue lysates of the liver. **k**. Determination of the ratio of reduced (GSH) to oxidized (GSSG) glutathione measured as an index oxidative stress. Data in bar graphs are shown as mean ± S.D. and represent 3 biological replicates (*n* = 3 independent assays for **e**-**k**). *P* values were calculated by two-sided unpaired *t*-tests (**e**-**k**) or one-way ANOVA (**c**-**d**). ^, *P* > 0.05. *, *P* < 0.05. **, *P* < 0.01. ***, *P* < 0.001. ****, *P* < 0.0001. Source data

As a minor but essential issue, we next sought to clarify whether the reduced mortality came at a cost of elevated late-life morbidity. To address this, we examined the physical function of experimental mice exposed to the vehicle, PDK4-IN or PCC1 (a senolytic control per case) monthly until death. Despite the longer remaining lifespan in PDK4-IN-treated animals, physical function in the final 2 months of life was not significantly lower than that of vehicle-treated mice (Extended Data Fig. 9k–m). According to autopsy datasets, the cause of mortality, incidence of several age-related diseases and tumor burden were not remarkably different between these groups (Extended Data Fig. 10a–d). Nevertheless, SASP expression was reduced in solid organs such as the liver, a tendency consistent with decreased circulating levels of IL-6, AREG and colony-stimulating factor 3 (CSF3), typical SASP markers in peripheral blood (Extended Data Fig. 10e–h).

Upon isolation of immune cells from peripheral blood, we observed reduced expression of the SASP in CD3^+^ T cell subpopulations (Extended Data Fig. 10i), a cell lineage displaying robust upregulation of p16^INK4a^ in the course of human aging^51^. PDK4-IN minimized oxidative stress in liver tissues, as evidenced by a significant decline of lipid peroxidation product 4-hydroxynonenal (HNE) adducts and a substantial increase in the ratio of reduced to oxidized glutathione (GSH:GSSG) (Extended Data Fig. 10j,k), data indicative of prominent benefits of PDK4 suppression in eliminating free radicals and engaging antioxidant defense system^52,53^.

Altogether, targeting PDK4 holds a remarkable potential to restrain the overall pathophysiological impact of senescent cells upon organismal aging, particularly systemic loss of tissue homeostasis and organ dysfunction resulting from lactate overproduced by these cells. As a technical advantage, PDK4-suppressing regimens can significantly extend lifespan without causing elevated morbidity. We hereby present proof-of-principle evidence that, even when administered in advanced stage, such a therapeutic strategy can remarkably postpone age-associated physical dysfunction, prevent age-related degeneration and optimize health conditions, thus establishing a senescence-related and metabolism-oriented avenue to improve the healthspan and lifespan of aged individuals.

## Discussion

Aging is a complex and time-dependent process that causes a progressive decline of physiological integrity, particularly functional degeneration of multiple organ types. Cellular senescence represents a primary risk factor for initiation and development of age-related conditions, such as cancers, diabetes, cardiovascular disorders and neurodegenerative diseases^4,54^. Senescent cells synthesize a large array of pro-inflammatory cytokines, chemokines and extracellular matrix degrading enzymes, a feature known as the SASP^5^. Discovery of the SASP proposes a reasonable and critical mechanism to explain why senescent cells, even accumulating in a low number in vivo during aging, can generate detrimental effects on organismal health; however, whether or not the SASP is the sole source of senescence-associated factors contributing to the loss of tissue homeostasis and organ function, remains yet unknown. In this study, we mapped the metabolic landscape of glucose metabolism and disclosed that senescent cells develop a substantially reprogrammed metabolism and produce an increased amount of metabolites, particularly the glycolysis product lactate; the latter is mediated by PDK4 upregulation and has the potential to alter the host microenvironment. With experimental models, we demonstrated that the consequences of such a metabolic rewiring include, but are not limited to, increased cancer malignancy, specifically drug resistance, and chronological aging accompanied by physical dysfunction in advanced stages.

Belonging to the PDK superfamily and acting as a glucose sensor, PDK4 has become an attractive target for treatment of various metabolic pathologies including hyperglycemia, insulin resistance and hepatic steatosis^55^. Upregulation of PDK4 mediates aerobic glycolysis (the ‘Warburg effect’), favors tumor growth and promotes apoptosis resistance^11,56–58^; however, potential implications of PDK4 in senescence-associated phenotypes remain hitherto underexplored. Our study established a PDK4 expression pattern upon cellular senescence, elucidated its role in diverging glucose metabolism toward glycolysis to produce lactate, and unraveled the correlation of PDK4 upregulation in tumor stroma and post-treatment patient survival. The upregulation of PDK4 in senescent cells is likely cell type- and context-dependent, as PDK4 downregulation has been reported in some cases, such as senescent IMR90 (refs. ^59,60^). Mainly inducible in stromal cells, PDK4 causes overproduction of lactate, a molecule that accumulates in treatment-damaged TME but ultimately enters systemic circulation. In our study, senescent cells (particularly TIS) displayed an enhanced level of glutamate, an intermediate that can be metabolically converted to α-ketoglutarate to enter the TCA cycle and promote ATP production. Although mechanisms supporting glutamate production, such as potential glutaminolysis from glutamine, remain to be determined in senescent cells, alternative pathways such as those involving fatty acid oxidation and lactate reverse metabolism cannot be simply excluded.

ROS cover several subspecies, including the superoxide anion (O_2_^−^), HP (H_2_O_2_) and the hydroxyl radical (OH∙), which are generated as byproducts of aerobic metabolism^61^. Various NOX isoforms appear heterogeneously in a wide variety of cells type and tissues, and are specialized in the deliberate production of ROS^62^. Although cells have evolved an antioxidant defense system to eliminate harmful ROS, excess ROS do override the antioxidant defense framework and cause oxidative damage to various macromolecules, a mechanism underlying the pathogenesis of diverse disorders and organismal aging^63^. Our data suggest the implication of NOX1 in modulating ROS production by senescent cells, illustrating an alternative but important source of senescence-associated stress signals, which can be generated via a positive feedback involving NOX1-activating lactate, a metabolite accessible through either an autocrine or a paracrine manner in tissues harboring senescent cells. In contrast, normal cells are not subject to such an effect mediated by the lactate–NOX1-ROS axis, which is attributed to the structural and functional integrity of their mitochondria, which likely exempts them from senescence-associated damages. For senescent cells, an increased ROS level can trigger modification of cellular redox balance in favor of overall oxidation. Multiple intracellular components undergo acute ROS-triggered damage, compromising the structural and functional integrity of proteins, lipids, particularly nucleic acids (including DNA), exacerbating macromolecular damage and further promoting the SASP expression by senescent cells. All of these changes will ultimately contribute to chronic inflammation and underpin many neurodegenerative, cardiovascular and metabolic conditions^64,65^. Although PDK4 expression represents a cell non-autonomous process in senescent cells, the impact of resulting lactate on senescence through inducing superoxide generation and deteriorating DNA damage, events culminating in enhanced SASP expression, does accelerate aging and organ degeneration. To the contrary, therapeutically targeting PDK4 itself or a ROS-mediated pathological process to abrogate the lactate-involved positive feedback of senescent cells, holds potential to antagonize organismal aging, minimize age-related chronic disorders and even prolong lifespan^43,44,66^.

Cellular senescence is functionally involved in tumor immune surveillance, mainly by initiating immune responses against antigens expressed in pre-malignant senescent cells that express the SASP^67^; however, the SASP can also promote cancer development such as in the case of obesity-associated hepatocellular carcinoma, where dietary or genetic obesity induces gut microbiota to produce deoxycholic acid, a gut bacterial metabolite that causes DNA damage and cellular senescence^68^. A recent study revealed the physiological role of senescent fibroblasts as tissue-resident sentinels in the stem cell niche by monitoring barrier integrity and rapidly responding to local inflammation to promote epithelial tissue regeneration after birth^69^. There is also evidence that acute or continuous depletion of p16^hi^ senescent cells compromises blood–tissue barriers with subsequent liver and perivascular tissue fibrosis in mid-aged animals, suggesting the contribution of senescent cells to structural and functional integrity in the aging organism. Together, these findings substantiate that senescent cells may exert beneficial or deleterious effects, depending on the pathophysiological context in vivo. In light of this, caution should be exercised that future application of PDK4 inhibitors against aging and associated diseases in clinical settings takes specific conditions of individual patients into account, to amplify the advantage of targeting senescent cells while avoiding therapeutic side effects.

Metabolism plays an important role in regulating cellular senescence, a process that dramatically affects the aging process^70^. By metabolic profiling and functional assessments of glucose consumption, we hereby mapped the metabolic landscape of human senescent cells, and propose that PDK4, a PDH-modifying enzyme, acts as a key regulator of biochemical activities to shape a unique form of metabolism, namely hypercatabolism, upon cellular senescence. Senescent cell accumulation is a major cause of age-related inflammation and predisposes the host to a number of chronic pathologies. The observation that PDK4 activity and lactate production are induced during senescence in some cells suggests that they may promote natural aging and age-related physical dysfunction; however, the extent to which PDK4 is upregulated in senescent cells, whether this upregulation is specific to a subset of senescent cells, as well as the mechanisms controlling its expression remain to be determined by future studies. Nonetheless, our data suggest that PDK4 could be a target to limit the overall pathological impact of senescent cells. Chronological age-associated cellular senescence in-tissue microenvironments bridges the gap between lactate overproduction, chronic inflammation and pathological events, whereas accumulation of lactate in solid organs is a pivotal and early event in various diseases^71,72^. Given that high PDK4 expression rewires energy metabolism, holds the potential to cause tissue homeostasis imbalance and overall physical dysfunction, our study raises the possibility that PDK4 is pharmacologically exploited for therapeutic intervention of natural aging and multiple age-related disorders, including but not limited to cancer.

## Methods

### Cell treatments

Stromal cells were grown until 80% confluent (CTRL) and treated with 50 μg ml^−1^ bleomycin (BLEO), 5 μM doxorubicin (DOX), 2 μM mitoxantrone (MIT), 50 nM docetaxel (DTX), 50 μM paclitaxel (PTX) or 20 μM vinblastine (VBL). After treatment, cells were rinsed briefly with PBS and kept for 7–10 d before various examinations were carried out. For experimental assays involving PDK4 inhibition, the anthraquinone derivative PDK4-IN was used at 5 μM. For MCT1/4 dual inhibition, syrosingopine was used at 10 μM. To induce mitochondrial dysfunction, rotenone (10 μM) or CCCP (10 μM) were employed. To enhance ROS production, the chemical lactate (10 mM) was applied. To inhibit oxidative phosphorylation and F0F1 ATP synthase activity, the agent Gboxin (500 nM) was used.

### Recruitment of human patients with cancer and biospecimen analysis

Administration of chemotherapeutic agents was performed for patients with primary PCa (Clinical Trials no. NCT03258320) and patients with infiltrating ductal BCa (NCT02897700), by following the CONSORT 2010 Statement (updated guidelines for reporting parallel group randomized trials). Patients with a clinical stage ≥I subtype A (IA) (T1a, N0, M0) of primary cancer but without manifest distant metastasis were enrolled into the multicentered, randomized, double-blinded and controlled pilot studies. Age between 40–75 years with histologically proven PCa or age ≥18 years with histologically proven infiltrating ductal BCa was required for recruitment into the clinical cohorts. Data regarding tumor size, histological type, tumor penetration, lymph node metastasis and TNM stage were obtained from the pathological records. Tumors were processed as formalin-fixed paraffin-embedded biospecimens and sectioned for histological assessment, with alternatively prepared OCT-frozen chunks processed via LCM for gene expression analysis. Specifically, stromal compartments associated with glands and adjacent to the cancer epithelium were separately isolated from tumor biopsies before and after chemotherapy using an Arcturus (Veritas Microdissection) LCM following previously defined criteria^14^. Immunoreactive scoring (IRS) gives a range of 1–4 qualitative scores according to staining intensity per tissue sample. Categories for the IRS include 0–1 (negative), 1–2 (weak), 2–3 (moderate) and 3–4 (strong)^73^. The diagnosis of PCa and BCa in tissues was confirmed based on histological evaluation by independent pathologists. Randomized controlled trial protocols and all experimental procedures were approved by the Institutional Review Board of the Shanghai Jiao Tong University School of Medicine, with methods carried out in accordance with the official guidelines. Informed consent was obtained from all participants and the experiments conformed to the principles defined in the WMA Declaration of Helsinki and the Department of Health and Human Services Belmont Report. Sex was not considered in the overall study design as the clinical investigations and principal conclusions were applicable to both sexes.

### Metabolic analysis

ECAR was measured with a Glycolysis Stress Test kit (Agilent Technologies, 103020-100), with OCR assessed using a Cell Mito Stress Test kit (Agilent Technologies, 103015-100). ECAR and OCR were determined with an XF24 Extracellular Flux Analyzer (Seahorse Bioscience, 01862) according to the manufacturer’s standard protocol. PSC27 was seeded at a density of 5 × 10^4^ cells per well in the XF24 cell culture microplate (Agilent Technologies, 04721 and Q01321) at 37 °C and 5% CO_2_ in an incubator overnight. To measure ECAR, 10 mM glucose, 1 μM oligomycin and 50 mM 2-DG were injected into each well. To measure the OCR, 1.5 μM oligomycin, 0.5 μM FCCP and 0.5 μM rotenone/antimycin were injected sequentially in order into each well. All Seahorse data were normalized to cell numbers, with all metabolic parameters automatically calculated by WAVE software equipped in the Seahorse. Values were calculated as follows: non-glycolytic acidification was referred to as the last rate measurement before glucose injection; the glycolysis rate was referred to as the maximum rate measurement before oligomycin injection (the last rate measurement before glucose injection); glycolytic capacity was referred to as the maximum rate measurement after oligomycin injection (the last rate measurement before glucose injection); for the OCR, basal respiration was referred to as last rate measurement before the first injection (the minimum rate measurement after rotenone/antimycin injection); and ATP production was referred to as last rate measurement before the oligomycin injection (the minimum rate measurement after oligomycin injection).

### Metabolite labeling and measurement by GC–MS

Cells were resuspended in 0.6 ml cold (−40 °C) 50% aqueous methanol containing 100 µM norvaline as an internal standard, inserted in dry ice for 30 min for thawing. Samples were added with 0.4 ml chloroform and vortexed for 30 s before centrifugation at 14,000*g* (4 °C) for 10 min, with the supernatant transferred to new 1.5-ml tubes for evaporation before storage at −80 °C. Metabolites were processed for GC–MS analysis as follows: first, 70 μl pyridine was added to the dried pellet and incubated for 20 min at 80 °C; after cooling, 30 µl *N-tert*-butyldimethylsilyl-*N*-methyltrifluoroacetamide (Sigma) was added, then samples were re-incubated for 60 min at 80 °C before centrifugation for 10 min at 14,000*g* (4 °C). The supernatant was transferred to an autosampler vial for GC–MS analysis. A Shimadzu QP-2010 Ultra GC–MS was programmed with an injection temperature of 250 °C and injected with 1-µl samples. GC oven temperature started at 110 °C for 4 min, before being raised to 230 °C at 3 °C min^−1^ and to 280 °C at 20 °C min^−1^ with a final hold at this temperature for 2 min. GC flow rate with helium carrier gas was 50 cm s^−1^, with the GC column used at 20 m × 0.25 mm × 0.25 mm Rxi-5ms. The GC–MS interface temperature was 300 °C, while ion source temperature (electron impact) was set at 200 °C with 70 V ionization voltage. The mass spectrometer was set to scan m/z range 50–800 with a 1-kV detector.

GC–MS data were analyzed to determine isotope labeling. To determine ^13^C labeling, the mass distribution for known fragments of metabolites was extracted from the appropriate chromatographic peak. These fragments contained either the whole carbon skeleton of the metabolite or lacked the α-carboxyl carbon or (for some amino acids) contained only the backbone minus the side chain. For each fragment, the retrieved data consisted of mass intensities for the lightest isotopomer (without any heavy isotopes, M0) and isotopomers with increasing unit mass (M1 to M6) relative to M0. These mass distributions were normalized by dividing by the sum of M0 to M6 and corrected for the natural abundance of heavy isotopes of the elements H, N, O, Si and C, using matrix-based probabilistic methods implemented in MATLAB. Labeling results are expressed as the average fraction of the particular compound containing the isotopic label from the particular precursor.

### Assessment of mitochondrial mass

Mitochondrial mass was technically appraised using MitoTracker Deep Red staining followed by cytation and super-resolution imaging. Briefly, cells were first stained with 500 nM MitoTracker Deep Red (Thermo Fisher) in 2 ml culture medium and incubated at 37 °C in 5% CO_2_ for 30 min, followed by fixation in 4% paraformaldehyde (PFA) dissolved in PBS. Fixed cells were then permeabilized in 0.2% Triton X-100 in PBS and blocked with 2% FBS in PBS before staining with Phallodin-AF568 and Hoechst (Thermo Fisher). Coverslips containing stained cells were mounted to slides with ProLong Gold Anti-Fade (Thermo Fisher) mounting medium. Imaging and analysis were performed on a Cytation 3 Cell Imaging Multi-Mode Reader (Biotek), with data acquisition and processing accomplished with Gen 5 software package (Biotek). Results from the cytation image analysis generally displayed an increase in functional mitochondrial mass (as seen by an averagely elevated MitoTracker fluorescence intensity) in senescent cells^74^.

### Intracellular H_2_O_2_ and mitochondrial superoxide measurement

The chemical agents DCFH-DA and MitoSOX allow determination of intracellular H_2_O_2_ and mitochondrial superoxide levels, respectively. DCFH-DA is a cell-permeable non-fluorescent probe with peroxide-selective dye that can passively diffuse into the intracellular matrix of cells, before being sheared by esterase and oxidized by H_2_O_2_, forming fluorescent DCF. MitoSOX is a superoxide indicator dye that specifically recognizes mitochondrial superoxide, producing red fluorescence in live cells. Briefly, cells in culture were loaded with DCFH-DA (10 μM, Beyotime) for 30 min or with MitoSOX (5 μM, Beyotime) for 10 min at 37 °C. Subsequently, all stained specimens were rinsed three times with PBS, then imaged under a fluorescence microscope or quantitatively measured for fluorescence intensity.

### Experimental animals and chemotherapeutic studies

All animals were maintained in a specific pathogen-free (SPF) facility using NOD/SCID (Nanjing Biomedical Research Institute of Nanjing University) mice at an age of approximately 6 weeks (~20 g body weight). All experimental mice were housed (22–25 °C, 30% humidity) under a 12-h light–dark cycle (6:00 to 18:00) with a standard rodent chow diet (5LOD, PicoLab) and water provided ad libitum. Ten mice were incorporated into each group and xenografts were subcutaneously generated at the hind flank upon anesthesia mediated by isoflurane inhalation. Stromal cells (PSC27 or HBF1203) were mixed with cancer cells (PC3, LNCaP, 22Rv1 or MDA-MB-231) at a ratio of 1:4 (250,000 stromal cells admixed with 1,000,000 cancer cells to make tissue recombinants before implantation in vivo). Animals were killed at 2–8 weeks after tumor xenografting, according to tumor burden or experimental requirements. Tumor growth was monitored every 3 d after certain time points, with tumor volume (*v*) measured and calculated according to the tumor length (*l*), width (*w*) and height (*h*) by the formula: *v* = (π/6) × ((*l* + *w* + *h*)/3)^3^ (ref. ^36^). Freshly dissected tumors were either snap-frozen or fixed to prepare formalin-fixed paraffin-embedded samples. Resulting sections were used for IHC staining against specific antigens or subject to H&E staining.

For chemoresistance studies, animals received subcutaneous implantation of tissue recombinants as described above and were given standard laboratory diets for 2 weeks to allow tumor uptake and growth initiation. Starting from the third week (tumors reaching 4–8 mm in diameter), MIT (0.2 mg kg^−1^ doses), DOX (doxorubicin, 1.0 mg kg^−1^ doses), therapeutic agent PDK4-IN (10.0 mg kg^−1^ doses, 200 μl per dose) or vehicle control was administered by i.p. injection (therapeutic agents via the i.p. route) on the first day of the third, fifth and seventh weeks, respectively. Upon completion of the 8-week therapeutic regimen, animals were killed, tumor volumes were recorded and tissues were processed for histological evaluation. All animals (mice) involved in prostate tumor-associated experiments were male, whereas those involved in breast tumor-associated assays were female.

At the end of chemotherapy and/or targeting treatment, animals were anesthetized and peripheral blood was gathered via cardiac puncture. Blood was transferred to a 1.5-ml Eppendorf tube and kept on ice for 45 min, followed by centrifugation at 9,000*g* for 10 min at 4 °C. Clear supernatants containing serum were collected and transferred to a sterile 1.5-ml Eppendorf tube. All serum markers were measured using dry-slide technology on IDEXX VetTest 8008 chemistry analyzer (IDEXX). Approximately 50 μl of the serum sample was loaded on the VetTest pipette tip before securely fitting on the pipettor, with manufacturer’s instructions followed for further examination.

For assessment of the impact of stromal PDK4 on tumor growth, ten mice were incorporated into each group and xenografts were subcutaneously generated at the hind flank upon anesthesia mediated by isoflurane inhalation. Stable sublines of stromal cells (PSC27) infected with lentivirus encoding PDK4-specific (#1 and #2) or scramble shRNA (C) were mixed with cancer cells (PC3) at a ratio of 1:4 (250,000 stromal cells admixed with 1,000,000 cancer cells to make tissue recombinants before implantation in vivo). Animals were killed 8 weeks after tumor xenografting, with final tumor volume (*v*) measured and calculated according to the tumor length (*l*), width (*w*) and height (*h*) by the formula: *v* = (π/6) × ((*l* + *w* + *h*)/3)^3^ (ref. ^36^). Tumors were monitored once every 3 d until the end of experiments to follow tumor growth, with animals tracked for health conditions to control distress. Using a three-dimensional measurement, the average size of tumors was not allowed to exceed 1.5 cm (diameter) or animals were killed immediately. For bulky disease evaluation, tumor volume was controlled at <2,000 mm^3^. The authors confirm that the maximal tumor size or burden was not exceeded throughout this study.

All animal experiments were performed in compliance with National Institutes of Health Guidelines for the Care and Use of Laboratory Animals and the ARRIVE guidelines and were approved by the Institutional Animal Care and Use Committee (IACUC) of the Shanghai Institute of Nutrition and Health, Chinese Academy of Sciences (protocol no. SINH-2022-SY-1).

### Senescent cell targeting and lifespan studies

For age-related studies, WT C57BL/6J mice (both males and females were involved, with sex generally not considered in the study design) were maintained in a SPF facility at 22–25 °C and 30% humidity under a 12-h light–dark cycle (6:00 to 18:00), with free access to a standard rodent chow diet (5LOD, PicoLab) and water provided ad libitum. The experimental procedure was approved by the IACUC at the Shanghai Institute of Nutritional and Health, Chinese Academy of Sciences, with all experiments conducted in accordance with the guidelines for animal experiments defined by the IACUC.

For preclinical studies of natural aging, 20-month-old non-transplanted WT C57BL/6J mice were used, which were generally sorted according to their body weight and randomly assigned to the vehicle, PDK4-IN or PCC1 treatments. Animals were treated once every 2 weeks in an intermittent manner for 4 months before undergoing physical tests at 24 months of age. For intervention trials involving lifespan extension at an advanced age, we employed animals at a very old age. Starting at 25–26 months of age (equivalent to human age of 80–85 years), mice (both sexes) were treated once every 2 weeks (biweekly) with the vehicle, PDK4-IN or PCC1 through i.p. injection (10.0 mg kg^−1^ and 20.0 mg kg^−1^ doses for PDK4-IN and PCC1, respectively) for three consecutive days. Some mice were moved from their original cages during the study to minimize single cage-housing stress. RotaRod (TSE system) and hanging tests were chosen for monthly measurement of maximal speed and hanging endurance, respectively, as these tests are considered sensitive and noninvasive. Animals were killed and scored as having died if they displayed more than one of the following signs: (1) incapable of drinking or eating; (2) reluctance to move, even after stimulus; (3) rapid weight loss; (4) severe balance disorder; or (5) bleeding or ulcerated skin. No mouse was lost due to fighting, accidental death or dermatitis. The Cox proportional hazard model was used for survival appraisal.

### Postmortem pathological examination

Mouse cages were checked every day, with dead animals being removed from the cages. Within 24 h, corpses were opened (abdominal cavity, thoracic cavity and skull) and preserved in 4% PFA individually for at least 7 d, with decomposed or disrupted bodies excluded. The preserved bodies were rendered to pathologists for blind examination, following an assessment routine. Briefly, tumor burden (sum of different types of tumors in each mouse), disease burden (sum of different histopathological changes of major organs in each mouse), severity of each lesion and inflammation (lymphocytic infiltrate) were assessed.

### Physical function appraisal

All physical tests were performed at least 5 d after the last dose of drug treatment. Maximal walking speed was measured using an accelerating RotaRod system (TSE system). Briefly, animals were trained on the RotaRod for 3 d at speeds of 4, 6, and 8 r.p.m. for 200 s on days 1, 2 and 3 for the coordination test. On the test day, mice were placed onto the RotaRod, which was started at 4 r.p.m. The rotating speed was accelerated from 4 to 40 r.p.m. over a 5-min interval. The speed was recorded when the mouse dropped off the RotaRod, with results averaged from three or four trials and normalized to the baseline speed. Training was not repeated for mice that had been trained within the preceding 2 months. The average values of each mouse were used for statistical inference.

Forelimb grip strength (N) was determined using a Grip Strength Meter (Columbus Instruments), with results averaged over ten trials. To measure grip strength the mouse is swung gently by the tail so that its forelimbs contact the bar. The mouse instinctively grips the bar and is pulled horizontally backward, exerting a tension. When the tension becomes overwhelming, the mouse releases the bars. For the hanging test, mice were placed onto a 2-mm-thick metal wire that was 35 cm above a padded surface, while animals were allowed to grab the wire with their forelimbs only. Hanging time was normalized to body weight as hanging duration (s) × body weight (g), with results averaged from two or three trials for each mouse. A Comprehensive Laboratory Animal Monitoring System (CLAMS; Columbus Instruments) was used to monitor daily activity and food intake over a 24-h period (12-h light–dark). The CLAMS system was equipped with an Oxymax Open Circuit Calorimeter System (Columbus Instruments). For treadmill performance, mice were acclimated to a motorized treadmill at an incline of 5° (Columbus Instruments) over 3 d for 5 min each day, starting at a speed of 5 m min^−1^ for 2 min and progressing to 7 m min^−1^ for 2 min and then 9 m min^−1^ for 1 min. On the test day, mice ran on the treadmill at an initial speed of 5 m min^−1^ for 2 min, and then the speed was increased by 2 m min^−1^ every 2 min until the mice were exhausted. The speed when the mouse dropped from the RotaRod was recorded, and results were averaged from three tests. Exhaustion was defined as the inability to return onto the treadmill despite a mild electrical shock stimulus and mechanical prodding. Distance was recorded and total work (kJ) was calculated using the following formula: mass (kg) × g (9.8 m s^−2^) × distance (m) × sin (5°).

In a subset of 8–10 mice per group, habitual ambulatory, rearing and total activity, oxygen consumption (VO_2_), and carbon dioxide production (VCO_2_) of individual animal were monitored over a 24-h period (12-h light–dark) using the CLAMS system equipped with an Oxymax Open Circuit Calorimeter System (Columbus Instruments). Ambulatory, rearing and total activities were summed and analyzed for light and dark periods under fed conditions. The VO_2_ and VCO_2_ values were used to calculate the respiratory exchange ratio (RER) and VO_2_. RER values were used to determine the basal metabolic rate (kcal kg^−1^ h^−1^).

### Statistics, reproducibility and sample size determination

All in vitro experiments were performed at least in triplicate, whereas animal studies were conducted with at least ten mice per group. Data are presented as mean ± s.d. except where otherwise indicated. GraphPad Prism (v.9.5.1) was used to collect and analyze data, with statistical significance determined according to individual settings. Cox proportional hazards regression model and multivariate Cox proportional hazards model analyses were performed with statistical software SPSS. Statistical significance was determined by two-tailed unpaired Student’s *t-*test, one- or two-way ANOVA, Pearson’s correlation coefficients test, Kruskal–Wallis, log-rank test, Wilcoxon–Mann–Whitney test or Fisher’s exact test. For all statistical tests, a *P* value < 0.05 was considered significant.

To determine the sample size, we began by setting the values of type I error (α) and power (1 − β) to be statistically adequate at 0.05 and 0.80, respectively^75^. We then determined *n* on the basis of the smallest effect we measured. If the required sample size was too large, we chose to reassess the objectives or to more tightly control the experimental conditions to reduce variance. Experimental data collection was randomized. For mouse experiments, all mice with the same genotype were randomly assigned to each group and independently followed the same age-dependent schedule in each experimental design. We did not exclude samples, animals or data points. The study was not sex-oriented and sex-based analyses were not performed, as the findings and overall conclusions are applicable to both sexes. Sample sizes were not predetermined by pilot studies and statistical methods were not used to predetermine sample sizes. In general, our sample sizes were similar to those reported in previous publications^46,76^. Data distribution was assumed to be normal but this was not formally tested. Data collection and analysis were not performed blind to the conditions of experiments involving characterization of senescence-associated phenotypes. All data were tested for normality and equalness of s.d., which determined the use of appropriate statistical tests, such as a parametric test versus a nonparametric test.

### Reporting summary

Further information on research design is available in the Nature Portfolio Reporting Summary linked to this article.

### Supplementary information

Supplementary Information Supplementary methods, Supplementary Figs. 1–8, supplementary figure legends, Supplementary Tables 1–4 and source data.

Reporting Summary

### Source data

Source Data Fig. 1 Statistical source data.

Source Data Fig. 1 Unprocessed western blots.

Source Data Fig. 2 Statistical source data.

Source Data Fig. 3 Statistical source data.

Source Data Fig. 4 Statistical source data.

Source Data Fig. 5 Statistical source data.

Source Data Fig. 6 Statistical source data.

Source Data Fig. 7 Statistical source data.

Source Data Fig. 7 Unprocessed western blots.

Source Data Fig. 8 Statistical source data.

Source Data Extended Data Fig. 1 Statistical source data.

Source Data Extended Data Fig. 1 Unprocessed western blots.

Source Data Extended Data Fig. 2 Statistical source data.

Source Data Extended Data Fig. 3 Statistical source data.

Source Data Extended Data Fig. 3 Unprocessed western blots.

Source Data Extended Data Fig. 4 Statistical source data.

Source Data Extended Data Fig. 4 Unprocessed western blots.

Source Data Extended Data Fig. 5 Statistical source data.

Source Data Extended Data Fig. 6 Statistical source data.

Source Data Extended Data Fig. 6 Unprocessed western blots.

Source Data Extended Data Fig. 7 Statistical source data.

Source Data Extended Data Fig. 8 Statistical source data.

Source Data Extended Data Fig. 9 Statistical source data.

Source Data Extended Data Fig. 9 Unprocessed western blots.

Source Data Extended Data Fig. 10 Statistical source data.

## Data Availability

Source data for all main figures and extended data figures are supplied with this paper. Experimental data supporting the plots within this paper and other findings of this study are available from the corresponding author upon reasonable request. The RNA-seq data generated in the present study have been deposited in the Gene Expression Omnibus database under accession codes GSE198110, GSE217808 and GSE222279. Source data are provided with this paper.
