# Author Correction: PDK4-dependent hypercatabolism and lactate production of senescent cells promotes cancer malignancy

**DOI:** 10.1038/s42255-024-01069-w

**Published:** 2024-06-03

**Authors:** Xuefeng Dou, Qiang Fu, Qilai Long, Shuning Liu, Yejun Zou, Da Fu, Qixia Xu, Zhirui Jiang, Xiaohui Ren, Guilong Zhang, Xiaoling Wei, Qingfeng Li, Judith Campisi, Yuzheng Zhao, Yu Sun

**Affiliations:** 1 Key Laboratory of Tissue Microenvironment and Tumour, Shanghai Institute of Nutrition and Health, University of Chinese Academy of Sciences, Chinese Academy of Sciences, Shanghai, China; 2 Department of Pharmacology, Institute of Aging Medicine, Binzhou Medical University, Yantai, China; 3 Department of Urology, Zhongshan Hospital, Fudan University, Shanghai, China; 4 Optogenetics & Synthetic Biology Interdisciplinary Research Center, State Key Laboratory of Bioreactor Engineering, Shanghai Frontiers Science Center of Optogenetic Techniques for Cell Metabolism, School of Pharmacy, East China University of Science and Technology, Shanghai, China; 5 Research Unit of New Techniques for Live-cell Metabolic Imaging, Chinese Academy of Medical Sciences, Beijing, China; 6 Department of General Surgery, Pancreatic Disease Institute, Ruijin Hospital, Shanghai Jiao Tong University School of Medicine, Shanghai, China; 7 Department of Pharmacology, Shandong Technology Innovation Center of Molecular Targeting and Intelligent Diagnosis and Treatment, Binzhou Medical University, Yantai, China; 8 Department of Endodontics, Shanghai Stomatological Hospital and School of Stomatology, Fudan University, Shanghai, China; 9 Shanghai Key Laboratory of Craniomaxillofacial Development and Diseases, Fudan University, Shanghai, China; 10 Department of Plastic & Reconstructive Surgery, Shanghai Ninth People’s Hospital, Shanghai Jiao Tong University School of Medicine, Shanghai, China; 11 Buck Institute for Research on Aging, Novato, CA USA; 12 Lawrence Berkeley National Laboratory, University of California, Berkeley, CA USA; 13 Department of Medicine and VAPSHCS, University of Washington, Seattle, WA USA

**Keywords:** Senescence, Cancer microenvironment, Ageing, Metabolism

Correction to: *Nature Metabolism* 10.1038/s42255-023-00912-w, published online 30 October 2023

In the version of the article initially published, in the phase row of Extended Data Fig. 1b, four images (RS, RAS, RAD and HP) were misused and have now been corrected in the HTML and PDF versions of the article. The original and revised Extended Data Fig. 1b can be seen below as Fig. 1.

**Fig. 1:**
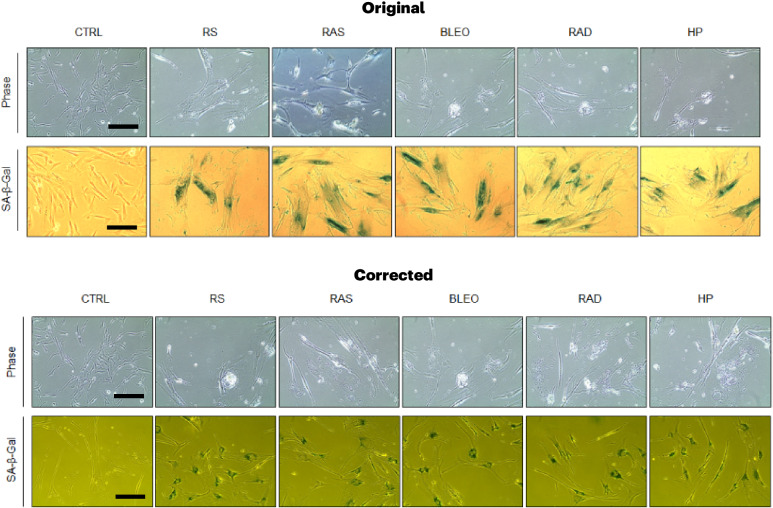
**Original and revised Extended Data Fig. 1b.**

