# Supplementary material for: PDK4-dependent hypercatabolism and lactate production of senescent cells promotes cancer malignancy
Source: Nat Metab. 2023 Oct 30;5(11):1887–910. doi: 10.1038/s42255-023-00912-w (PMC10663165; doi:10.1038/s42255-023-00912-w)
Supplement: Supplementary file 1 — Supplementary methods, Supplementary Figs. 1–8, supplementary figure legends, Supplementary Tables 1–4 and source data. [file 42255_2023_912_MOESM1_ESM.pdf]

nature metabolism

Article

https://doi.org/10.1038/s42255-023-00912-w

PDK4-dependent hypercatabolism and lactate production of senescent cells promotes cancer malignancy

In the format provided by the authors and unedited

Table of Contents

Supplementary Methods

Supplementary Figures

Supplementary Figure Legends

Supplementary Tables

Source Data (uncropped scans of blots and gels)

Supplementary Methods

Cell culture. Primary normal human prostate stromal cell line (PSC27) and breast stromal cell line (HBF1203) were generously provided by Dr. Peter Nelson (Fred Hutchinson Cancer Research Center) and maintained in stromal complete medium as described previously [1]. Human fetal lung stromal lines WI38, IMR90 and HFL1, foreskin stromal line BJ and human embryonic kidney line 293T were from ATCC and cultured with F-12K medium supplemented with 10% FBS. Prostate cancer epithelial cell lines PC3, DU145, LNCaP and VCaP, as well as breast cancer cell line MDA-MB-231 (ATCC) were routinely cultured with RPMI 1640 (10% FBS). Prostate cancer epithelial line M12 was a kind gift from Dr. Stephen Plymate (University of Washington), which originally derived from the benign line BPH1 but phenotypically neoplastic and metastatic [2]. All lines were routinely tested for mycoplasma contamination and authenticated with STR assays.

Vectors, viruses and infection. Full length human $HRAS^{G12V}$, *PDK4* and *PRKN* sequences were cloned into pLenti-CMV/To-Puro-DEST2 (Invitrogen), individually, as described [1]. Small hairpin RNAs (shRNA) targeting sequences for specific genes were cloned in pLKO.1-Puro vector (Addgene). Upon production by 293T cells, lentivirual titers were adjusted to infect ~ 90% of cells. Stromal cells were infected overnight in the presence of polybrene (8 μg/ml), allowed to recover for 48 h and selected for 72 h before subject to further analysis. For expression of target genes in either stromal or epithelial cells, total RNA was prepared and subject to qRT-PCR assays (primers listed in Supplementary Table 2, Supplementary Information).

RNA-seq and bioinformatics analysis. Total RNA samples were obtained from PC3 and DU145 cells cultured with CM of either $PSC27^{Vector}$ or $PSC27^{PDK4}$. Sample quality was validated by Bioanalyzer 2100 (Agilent), and RNA was subjected to sequencing by Illumina NovaSeq 6000 with gene expression levels quantified by the software package RSEM (https://deweylab.github.io/RSEM/). Briefly, rRNAs in the RNA samples were eliminated using the RiboMinus Eukaryote kit (QIAGEN), and strand-specific RNA-seq libraries were

constructed using the TruSeq Stranded Total RNA preparation kits (Illumina) according to the manufacturer's instructions before deep sequencing.

Pair-end transcriptomic reads were mapped to the reference genome (GRCh38.p13) (http://asia.ensembl.org/Homo_sapiens/Info/Index) (ensembl_105) with reference annotation from Gencode v27 using the Bowtie tool. Duplicate reads were identified using the picard tools (1.98) script mark duplicates (https://github.com/broadinstitute/picard) and only non-duplicate reads were retained. Reference splice junctions were provided by a reference transcriptome (Ensembl build 73) [3]. FPKM values were calculated with differential gene expression called by the Cuffdiff maximum-likelihood estimate function [4]. Genes of significantly changed expression were defined by a false discovery rate (FDR)-corrected P value < 0.05. Only ensembl genes 73 of status “known” and biotype “coding” were used for downstream analysis.

Reads were trimmed using Trim Galore (v0.3.0) (http://www.bioinformatics.babraham.ac.uk/projects/trim_galore/) and quality assessed using FastQC (v0.11.5) (http://www.bioinformatics.bbsrc.ac.uk/projects/fastqc/). Differentially expressed genes were subsequently analyzed for enrichment of biological themes using the DAVID bioinformatics platform (https://david.ncifcrf.gov/), the Ingenuity Pathways Analysis (IPA) program (http://www.ingenuity.com/index.html). Raw data of RNA-seq were deposited in the NCBI Gene Expression Omnibus (GEO) database under the accession code GSE198110, GSE217808 and GSE222279.

Venn diagrams

Venn diagrams and associated empirical P-values were generated using the USeq (v7.1.2) tool IntersectLists [5]. The t-value used was 22,008, as the total number of genes of status “known” and biotype “coding” in ensembl genes 73 (Sanger UK). The number of iterations used was 1,000.

RNA-seq heatmaps

For each gene, the FPKM value was calculated based on aligned reads, using Cufflinks [4]. Z-scores were generated from FPKMs. Hierarchical clustering was performed using the R package heatmap.2 and the distfun = “pearson” and hclustfun = “average”.

Immunoblot and immunofluorescence analysis. Whole cell lysates were prepared using RIPA lysis buffer supplemented with protease/phosphatase inhibitor cocktail (Biomake). Nitrocellulose membranes were incubated overnight at 4 °C with primary antibodies listed in Supplementary Table 4, while HRP-conjugated goat anti-mouse or anti-rabbit served as secondary antibody (Vazyme). For immunofluorescence analysis, cells were fixed with 4% formaldehyde and permeabilized before incubation with primary and secondary antibodies, each for 1 hr. Upon counterstaining with DAPI (0.5 μg/ml), samples were examined with an Imager A2. Axio (Zeiss) upright microscope to analyze specific gene expression.

Histology and immunohistochemistry. Formalin-fixed paraffin-embedded (FFPE) tissue sections of 7-10 μm were deparaffinized in xylenes and rehydrated through a graded series of alcohols. Routine histology appraisal was performed with hematoxylin and eosin (H&E) staining. For immunohistochemical (IHC) evaluation, FFPE sections experienced antigen retrieval with sodium citrate, incubation with 3% H_2O_2, treatment with avidin/biotin blocking buffer (Vector Laboratories) and then 3% BSA for 30 min. Staining with primary and secondary antibodies was conducted at 4°C for overnight and at room temperature for 60 min, respectively. Sections were incubated with a H_2O_2-diaminobenzidine (DAB) substrate kit (Vector, SK-4100). Samples were counterstained with hematoxylin, dehydrated and mounted. IHC images were obtained using an upright microscope (Olympus BX51). Brown staining indicates the immunoreactivity of samples.

***In vitro* cell phenotypic characterization.** For proliferation assays of cancer cells, 2×10^4 cells were dispensed into 6 well-plates and co-cultured with the conditioned media from stromal cells. Three d later, cells were digested and counted with hemacytometer. For migration assays, cells were added to the top chambers of transwells (8 μm pore), while stromal CM were given to the bottom. Migrating cells in the bottom chambers were stained by DAPI 12-24 h later, with samples examined with an Observer A1. Axio (Zeiss) inverted

microscope. Invasion assays were performed similarly with migration experiments, except that transwells were coated with basement membrane matrix (phenol red free, Corning). Alternatively, cancer cells were subject to wound healing assays conducted with 6-well plates, with healing patterns graphed with brightfield microscopy. For chemoresistance assays, cancer cells were incubated with stromal CM, with the chemotherapeutic agent MIT provided in wells for 3 d at each cell line's IC50, a value experimentally predetermined. Cell viability was assayed with a CCK-8 kit, with the absorbance at 450 nm measured using a microplate reader.

Lactate production assay. Lactate production was measured using a Lactate Colorimetric Assay Kit (Sigma-Aldrich, MAK058). Cells were homogenized in lactate assay buffer and centrifuged at 13,000 *g* for 10 min to remove insoluble materials. The supernatants were de-proteinized with a 10 kDa MWCO spin filter to remove other enzymes. Next, 50 ml of the supernatants were mixed with 50 ml of the reaction mix, with the reaction incubated for 30 min at room temperature. Lactate levels were measured at 450 nm using a microplate reader, with the relative level of lactate in all groups calculated and normalized to protein concentration.

Glucose uptake assay. The level of glucose uptake was measured using a Glucose Uptake-Glo Assay Kit (Promega, J1341), which provided a homogeneous bioluminescent method for assessing glucose uptake in mammalian cells based on the detection of 2-deoxyglucose-6-phosphate (2DG-6-P). Cells were removed from medium, then washed with PBS, afterwards 50 ml of 1mM 2-deoxyglucose (2DG) was added to the cells and incubated for 10 min at room temperature. Then 25 ml of acid detergent solution (stop buffer) were added to lyse the cells and terminate the uptake; 25 ml of high-pH buffer solution (neutralization buffer) were then added to neutralize the acid. Finally, 100 ml of 2DG-6-P detection reagent were added to the sample wells, with the reaction incubated at room temperature for 1-2 h. A Cytation 5 Cell Imaging Multi-Mode Reader was used to assay the luminescence. The relative level of glucose uptake in all groups was calculated and normalized to protein concentration.

Characterization of SoNar and FiLa *in vitro*

Purified protein was stored at -80 °C before experimental assays. For *in vitro* measurements, the purified sensor protein was diluted with 100 mM HEPES buffer containing 100 mM NaCl (pH 7.4). Fluorescence spectroscopy was performed on a fluorescence spectrophotometer (PerkinElmer, FL6500). Excitation spectra were recorded at an emission wavelength of 530 nm. Slit width was set as 10 nm bandpass and the PMT voltage was set at 500 V.

For nucleotide titration of SoNar and lactate titration of FiLa, the sensor protein was diluted in HEPES buffer (pH 7.4) to a final concentration of 0.2 μM as described previously [6]. The fluorescence intensity was measured by a filter-based Synergy Neo 2 Multi-Mode microplate reader using 420 BP 20 nm or 485 BP 20 nm excitation and 532 BP 40 nm emission band-pass filters (BioTek). All solutions were prepared in HEPES buffer (pH 7.4). Each assay was performed in a 96-well black bottom plate using 50 μl of substrate and 50 μl of sensor protein. Fluorescence intensity was measured immediately.

Live-cell fluorescence imaging

For fluorescence imaging, normal and senescent (TIS) PSC27 cells stably expressing SoNar, iNapc, FiLa or FiLa-C were plated on 35 mm 4-chamber glass-bottom dish. The dosing group was treated with or without 5 μM PDK4 inhibitor for 1 h. Fluorescence images were acquired using a Leica TCS SP8 SMD confocal laser-scanning microscope system with HC Plan Apo CS2 63×1.40 NA oil objective. For dual-excitation ratio imaging, 405 nm excitation laser and 488 nm excitation laser with an emission range of 500-550 nm were used. Raw data were exported to ImageJ software as 12-bit TIF for analysis. The pixel-by-pixel ratio of the 405 nm excitation image by the 488 nm excitation image of the same cell was used to pseudo-color the images in HSB color space as previously described [7, 8].

Tissue SA-β-Gal staining and histological examination. For SA-β-Gal staining, frozen sections were dried at 37 °C for 20-30 min before fixed for 15 min at room temperature. The

frozen sections were washed thrice with PBS and incubated with SA-β-Gal staining reagent (Beyotime) overnight at 37 °C. After completion of SA-β-Gal staining, sections were stained with eosin for 1-2 min, rinsed under running water for 1 min, differentiated in 1% acid alcohol for 10-20 sec, and washed again under running water for 1 min. Sections were dehydrated in increasing concentrations of alcohol and cleared in xylene. After drying, samples were examined under a bright-field microscope.

Liver, lung, prostate and cardiac tissue frozen sections stained with SA-β-Gal were quantified by ImageJ software (NIH) to measure the SA-β-Gal$^+$ area. The total region was quantified by eosin-positive area, while relative quantities of SA-β-Gal$^+$ cells were calculated with the SA-β-Gal$^+$ area divided by the total area. For the statistics of SA-β-Gal$^+$ area of lung, regions of lung were randomly selected to be photographed, avoiding analysis of larger pulmonary blood vessels and the trachea. For statistical analysis of SA-β-Gal-positive area of the liver, regions were randomly selected to be photographed. Each tissue was measured over 10-15 regions. The stained tissue sections were then observed with an Eclipse Ti-S (Nikon) microscope under bright-field illumination. For histochemical staining of the lung, tissues were preserved in OCT compound before cryo-sectioned and stained with H&E reagents. To quantify the alveolar size *via* ImageJ, we defined the areas with the Huang threshold, then measured average areas greater than 1,500 and 2,000 and 40 pixels, respectively, in 3 fields *per* section.

SASP factor production measurement. The amounts of hallmark SASP factors secreted by human senescent stromal cells (PSC27) were quantified in the conditioned media (CM) by enzyme-linked immunosorbent assay (ELISA) with human ELISA kits (ThermoFisher). Specifically, protein levels of IL1α, IL1β, IL6, IL7, CXCL1, CXCL8, CXCL12, MMP1, MMP3, MMP10, M-CSF, GM-CSF, AREG, TIMP1, BMP6, ANGPTL4, HGF and FGF7 were measured using the standard quantitative sandwich ELISA (Cat. no. BMS243-2, KHC0011, BMS213-2, EHIL7, BMS2122, BMS204-3, EHCXCL12A, EHMMP1, BMS2014-3, EHMMP10, EHCSF1, BMS283, EHAREG, EH456RB, EHBMP6, EHANGPTL4, KAC2211, EHFGF7, respectively), with data normalized to the volume of

CM and the number of cells producing the CM.

***In vivo* cytotoxicity evaluation by blood tests.** For routine blood examination, 100 μl fresh blood were acquired from each animal and mixed with EDTA immediately. The blood samples were analyzed with Celltac Alpha MEK-6400 series hematology analyzers (Nihon Kohden). For serum biochemical analyses, blood samples were collected and clotted for 2 h at room temperature or overnight at 4 °C. Samples were then centrifuged (1000 *g*, 10 min) to obtain serum. An aliquot of approximately 50 μl serum was subjected to analysis for creatinine, urea, alkaline phosphatase (ALP) and alanine transaminase (ALT) by an automatic biochemical analyzer (BS-5800M, Mindray Bio-Medical Electronics Co. Ltd). Evaluation of circulating levels of hemoglobin, white blood cells, lymphocytes and platelets were performed using dry-slide technology on a VetTest 8008 chemistry analyzer (IDEXX) as reported previously [9].

All animal experiments were conducted in compliance with the NIH Guide for the Care and Use of Laboratory Animals (National Academies Press, 2011) and the ARRIVE guidelines, and were approved by the IACUC of Shanghai Institute of Nutrition and Health, Chinese Academy of Sciences. For each preclinical regimen, animals were monitored for conditions including hypersensitivity (changes in body temperature, altered breathing and ruffled fur), body weight, mortality, and changes in behavior (i.e., loss of appetite and distress), and were disposed of appropriately according to the individual pathological severity as defined by relevant guidelines.

M**easurement of AST, ALT and LDH levels in serum.** Circulating blood was taken from mice through cardiac puncture and left at room temperature for 15 min. To obtain serum, blood samples were centrifuged at 3,500 *g* for 15 min at 4 °C. Any serum sample with severe haemolysis was excluded from further analyses of AST, ALT and LDH. Serum levels of AST, ALT and LDH were examined individually by commercial kits (Mindray).

Assessment of lipid peroxidation. The levels of 4-hydroxynonenal (HNE)-protein adducts in liver tissue lysates prepared in RIPA buffer were assessed in livers of mice using the OxiSelect HNE Adduct Competitive ELISA Kit (Cell Biolabs), as formerly described [10].

Appraisal of glutathione. Mouse livers fixed in 5% sulfosalicylic acid were prepared and analyzed for the concentration of reduced (GSH) and oxidized (GSSG) glutathione using a Glutathione Assay Kit (Cayman Chemical) as previously described [10]. Sample absorbance was detected at a wavelength of 405 nm using a plate reader and the ratio of GSH: GSSG was obtained for each tested tissue sample.

Supplementary references

1. Sun, Y. *et al.* Treatment-induced damage to the tumor microenvironment promotes prostate cancer therapy resistance through WNT16B. *Nat Med* **18**, 1359-1368 (2012).
2. Bae, V.L. *et al.* Metastatic sublines of an SV40 large T antigen immortalized human prostate epithelial cell line. *The Prostate* **34**, 275-282 (1998).
3. Zerbino, D.R., Wilder, S.P., Johnson, N., Juettemann, T. & Flicek, P.R. The ensembl regulatory build. *Genome biology* **16**, 56 (2015).
4. Trapnell, C. *et al.* Differential gene and transcript expression analysis of RNA-seq experiments with TopHat and Cufflinks. *Nat Protoc* **7**, 562-578 (2012).
5. Nix, D.A., Courdy, S.J. & Boucher, K.M. Empirical methods for controlling false positives and estimating confidence in ChIP-Seq peaks. *BMC Bioinformatics* **9**, 523 (2008).
6. Li, X. *et al.* Ultrasensitive sensors reveal the spatiotemporal landscape of lactate metabolism in physiology and disease. *Cell Metab* **35**, 200-211 e209 (2023).
7. Zhao, Y. *et al.* SoNar, a Highly Responsive NAD+/NADH Sensor, Allows High-Throughput Metabolic Screening of Anti-tumor Agents. *Cell Metab* **21**, 777-789 (2015).
8. Zhao, Y. *et al.* Genetically encoded fluorescent sensors for intracellular NADH detection. *Cell Metab* **14**, 555-566 (2011).
9. Chen, F. *et al.* Targeting SPINK1 in the damaged tumour microenvironment alleviates therapeutic resistance. *Nat Commun* **9**, 4315 (2018).
10. Robinson, A.R. *et al.* Spontaneous DNA damage to the nuclear genome promotes senescence, redox imbalance and aging. *Redox Biol* **17**, 259-273 (2018).

Supplementary Fig. 1

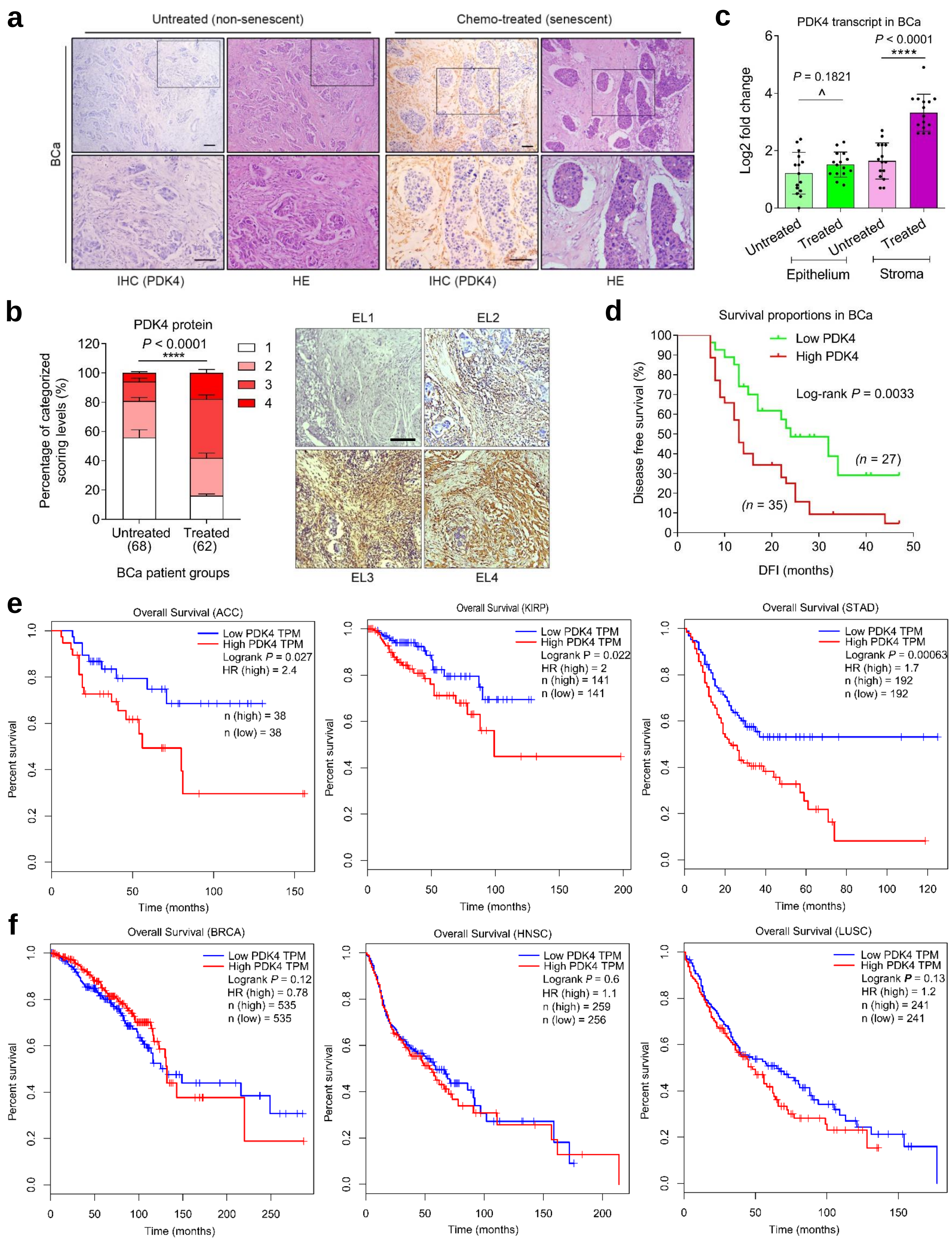

Supplementary Fig. 2

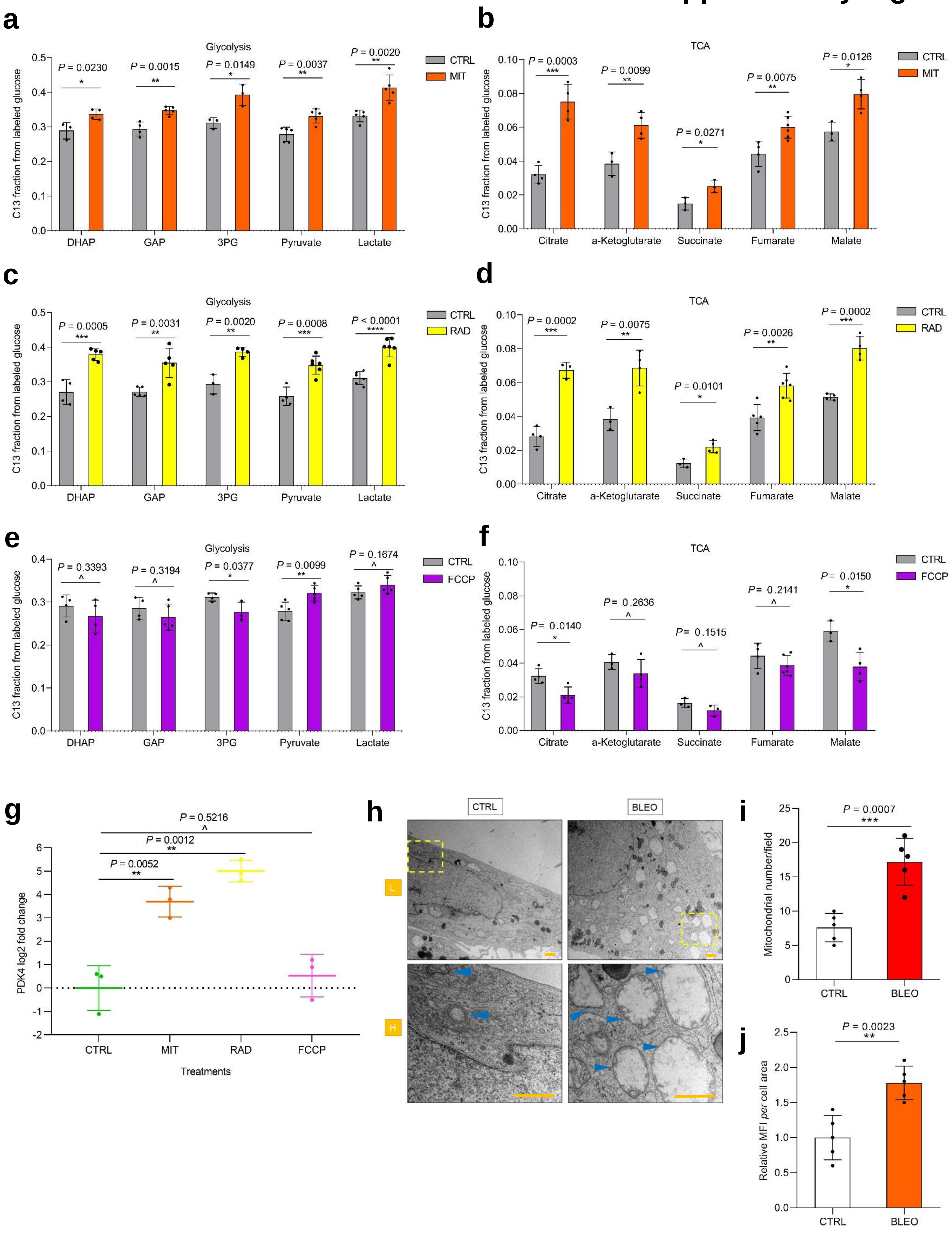

Supplementary Fig. 3

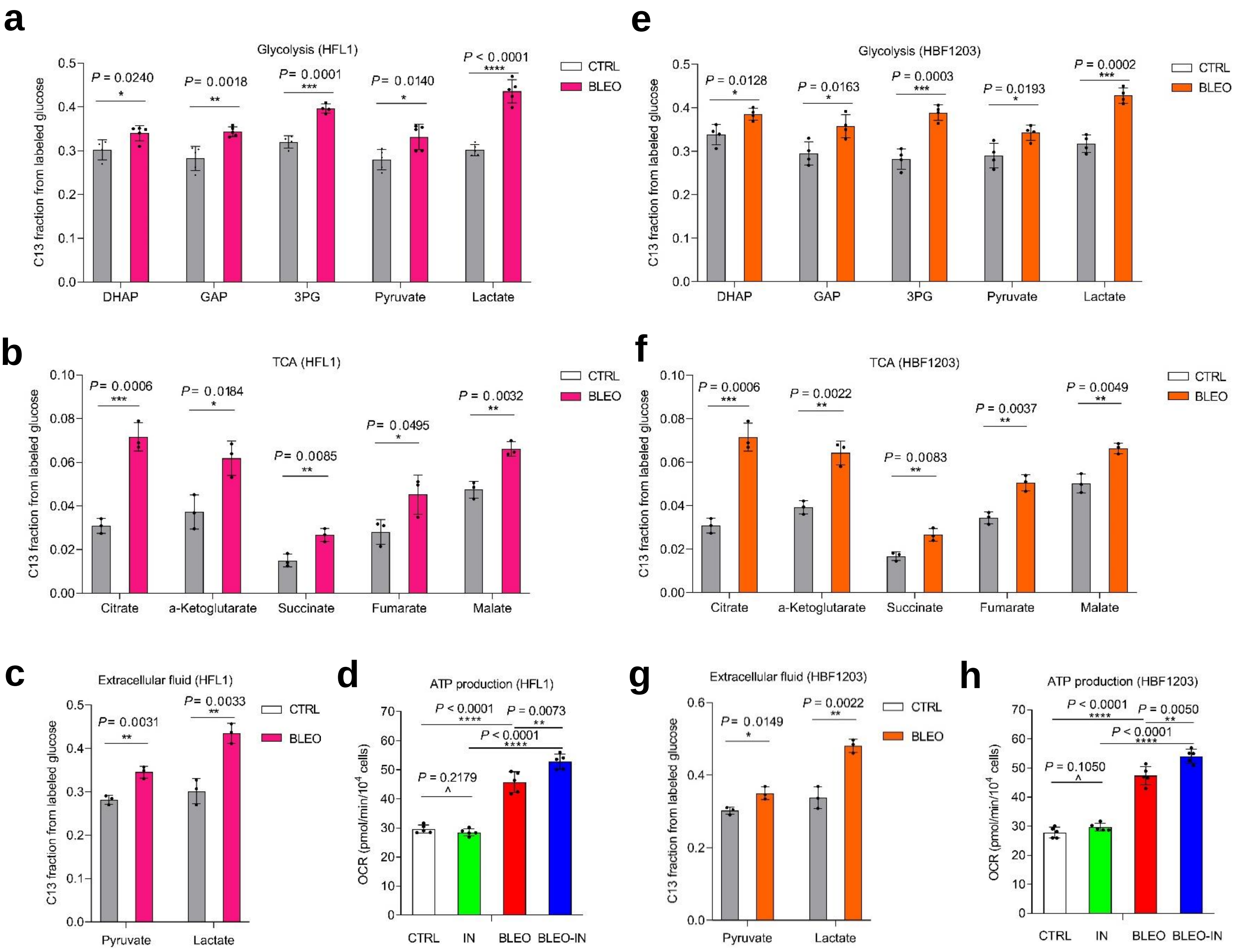

Supplementary Fig. 4

a

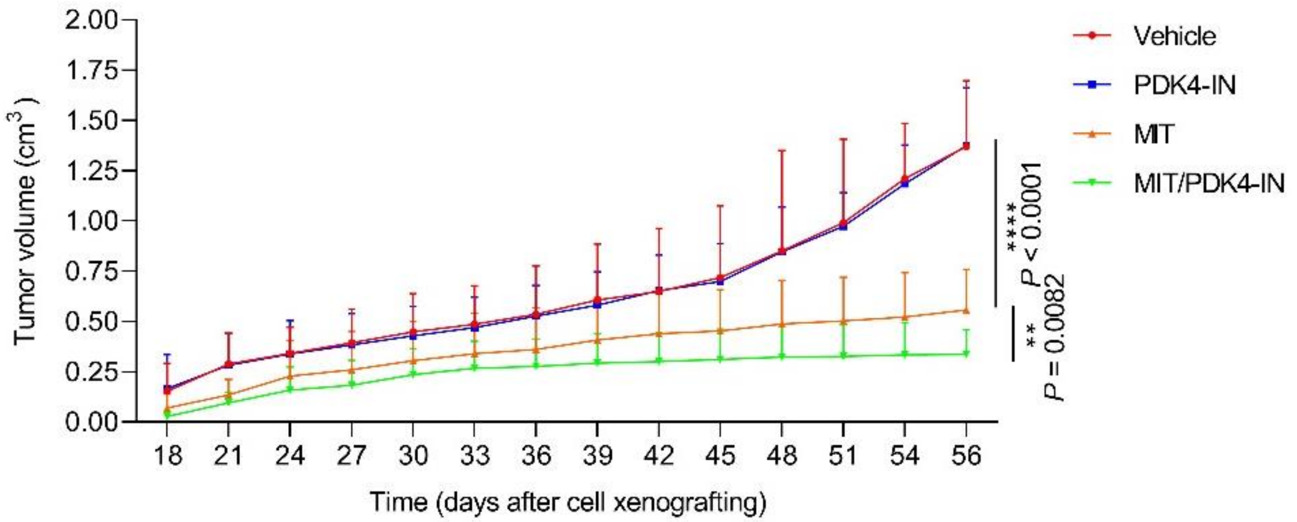

Supplementary Fig. 5

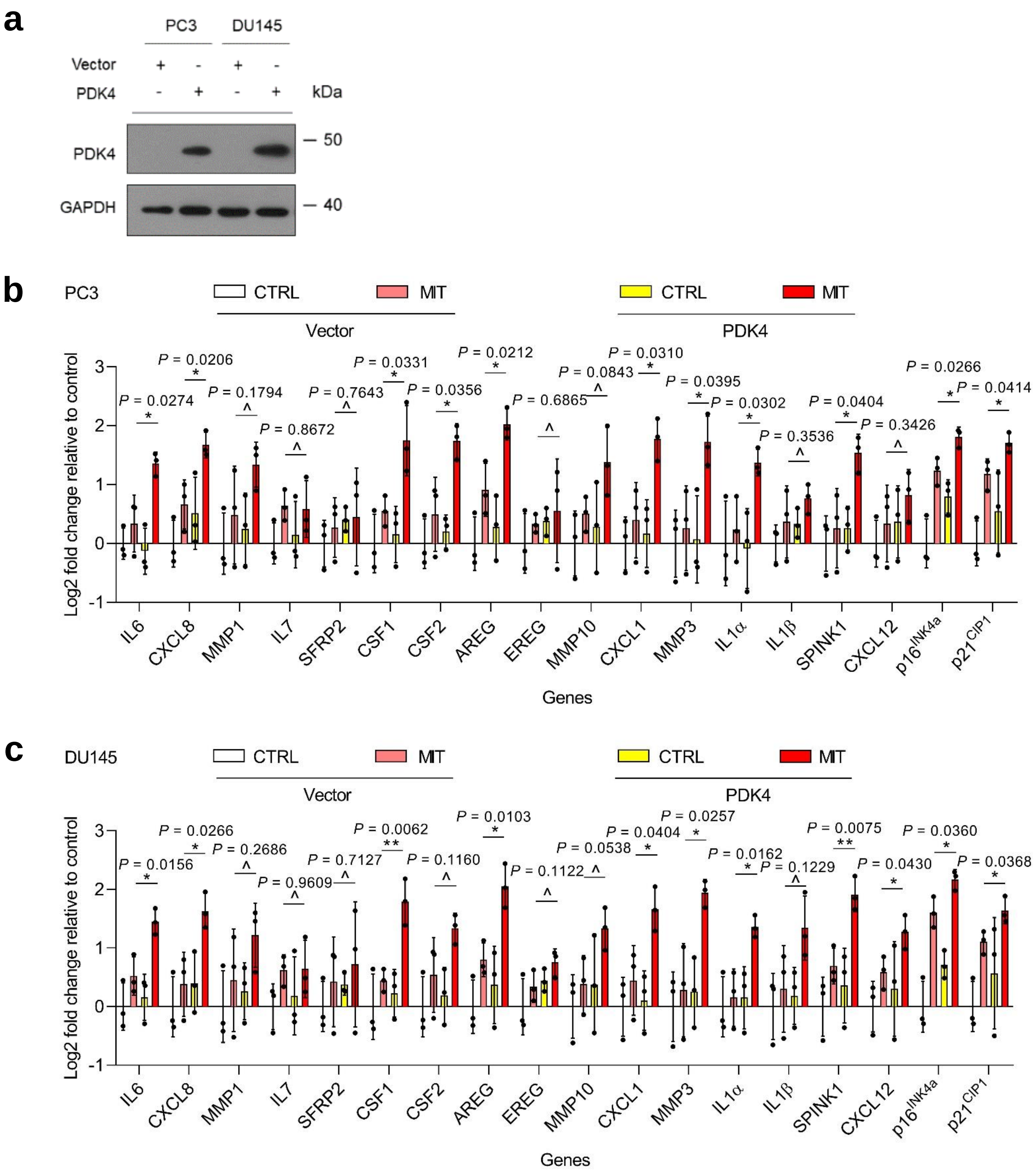

Supplementary Fig. 6

a

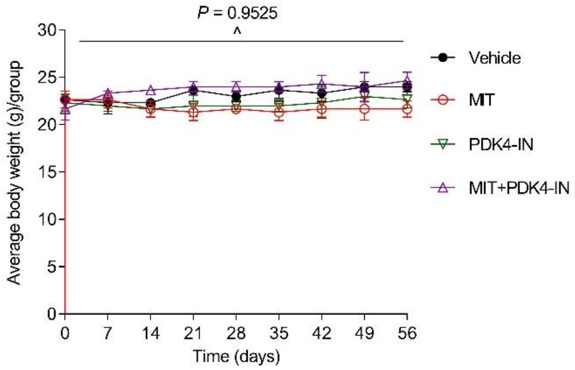

b

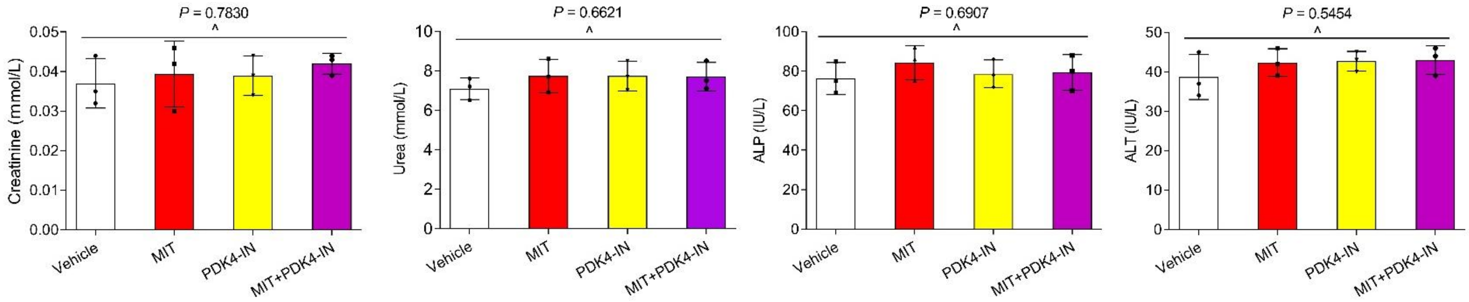

c

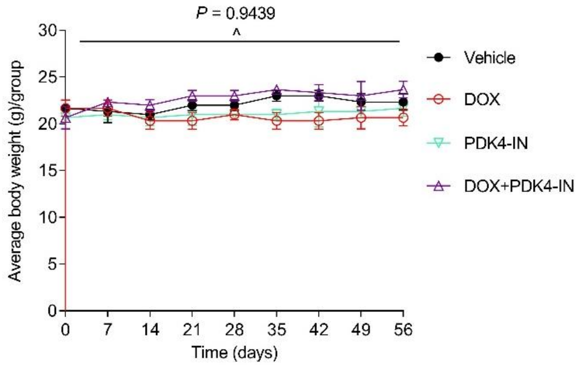

d

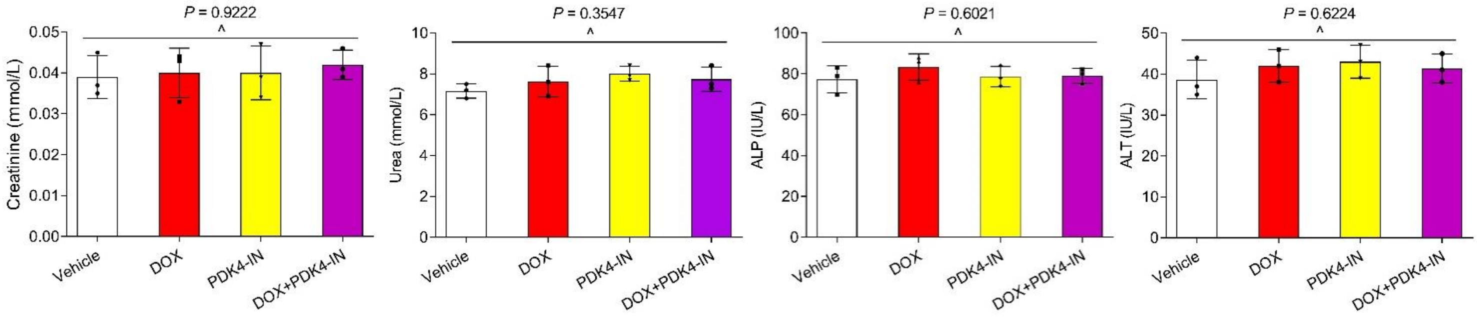

Supplementary Fig. 7

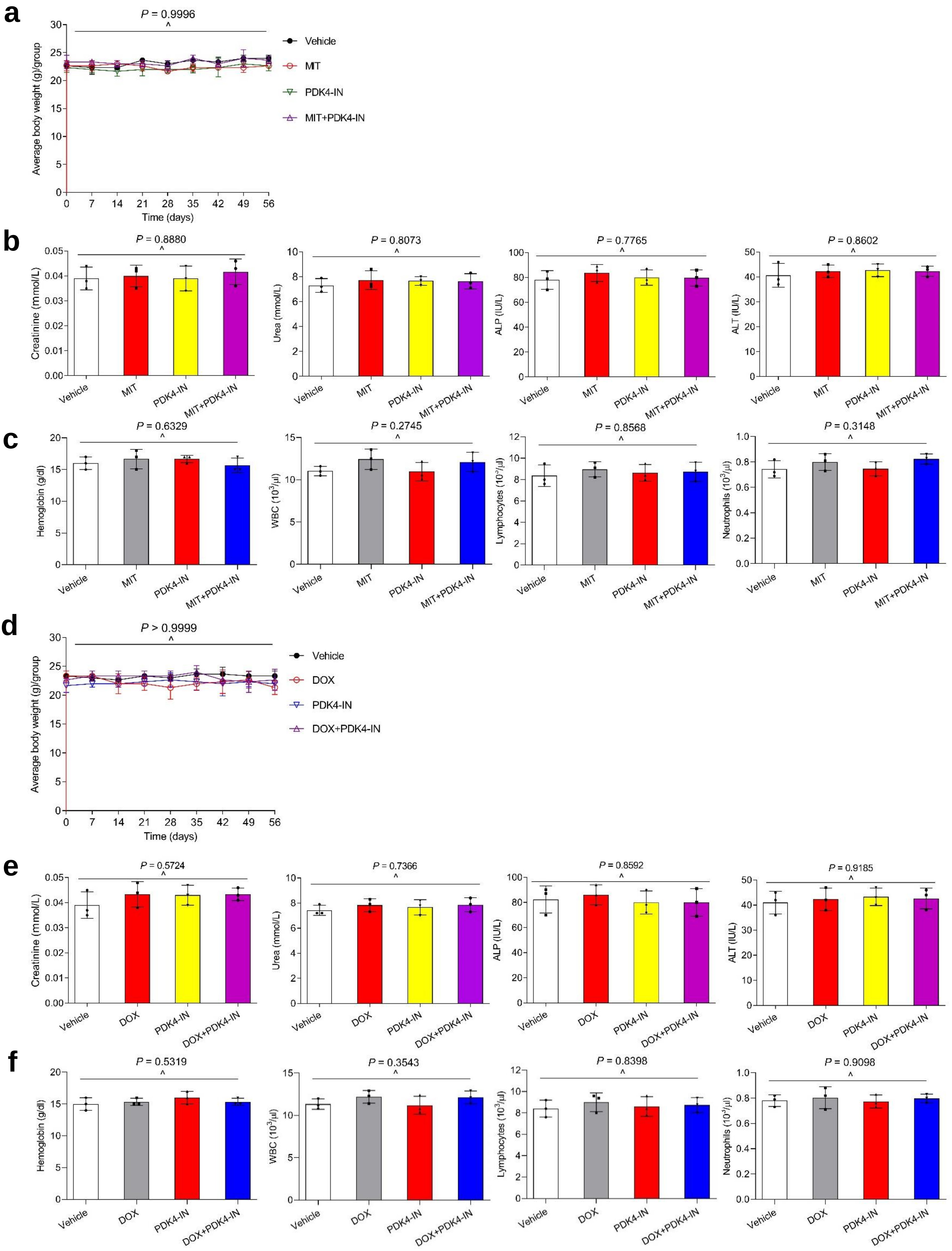

Supplementary Fig. 8

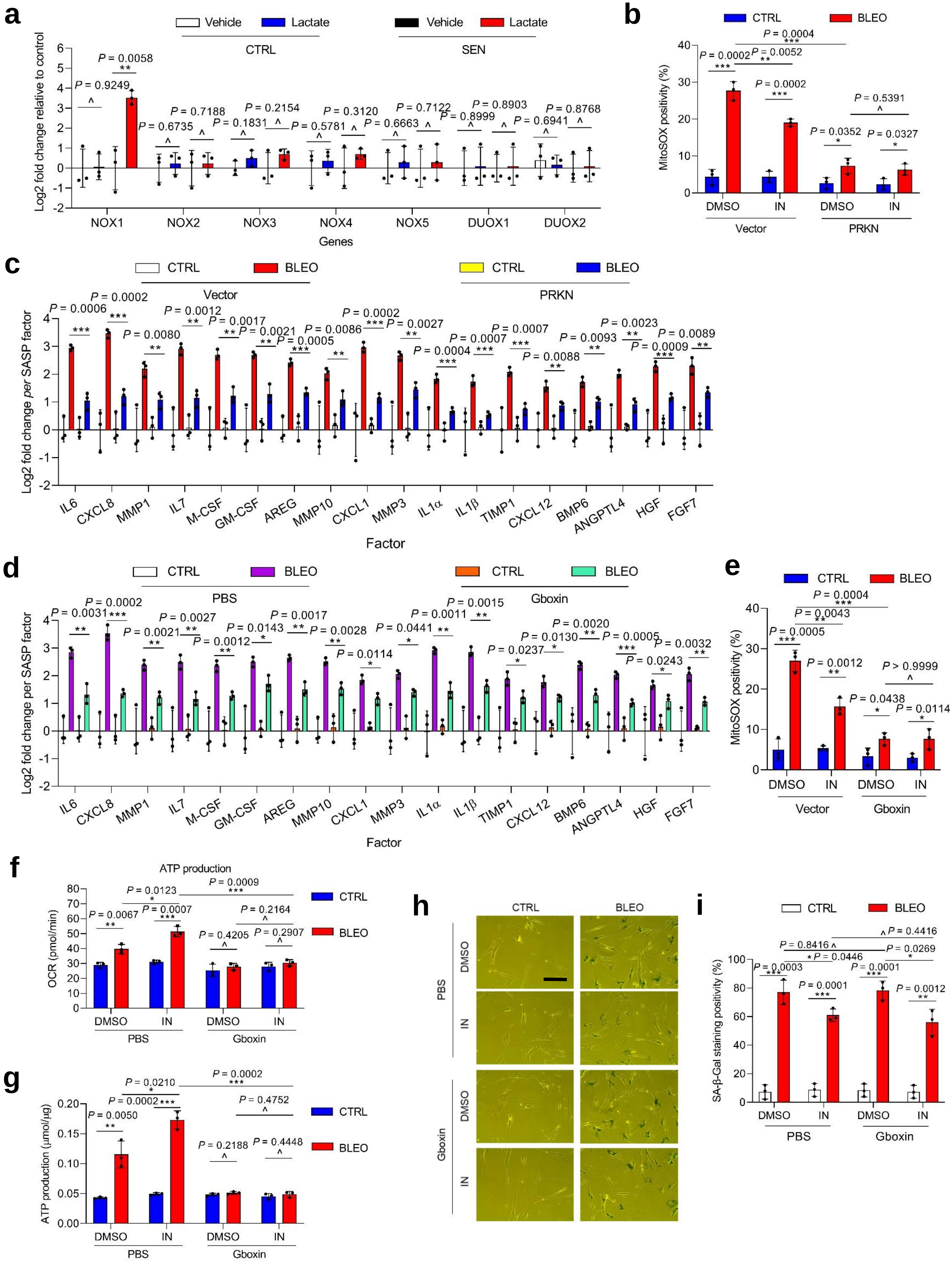

Supplementary Fig. 1. PDK4 expression in human breast stroma after chemotherapy and correlation with posttreatment survival.

a. Representative images of PDK4 expression in biospecimens of human breast cancer (BCa) patients. Left, untreated; right, chemo-treated. Rectangular regions selected in upper images *per* staining amplified into lower images. Scale bars, 100 μm.

b. Pathological assessment of stromal PDK4 expression in BCa samples. Patients pathologically assigned into 4 categories *per* IHC staining intensity of PDK4 in stroma. 1, negative; 2, weak; 3, moderate; 4, strong expression. Left, statistical comparison. Right, representative images of each category regarding PDK4 signals. EL, expression level. Scale bar, 100 μm.

c. Boxplot summary of PDK4 transcript expression upon LCM of cells from tumor and stroma, respectively. Signals normalized to the lowest value in untreated epithelium group, with comparison performed between untreated and treated samples *per* cell lineage. For cells of either epithelium or stroma origin, samples from 15 patients out of untreated and treated groups randomly selected.

d. Kaplan-Meier analysis of BCa patients. Disease free survival (DFS) stratified according to PDK4 expression. DFS represents the length (months) of period calculated from date of BCa diagnosis to point of first time disease relapse. HR, hazard ratio. BCa patients (62 totally) were from treated group of **b**.

e. Overall survival (OS) of patients diagnosed with malignancies and displaying significant correlation with PDK4 in cancer cells, figures profiled from the TCGA database. ACC. adrenocortical carcinoma. XIRP, kidney renal papillary cell carcinoma. STAD, stomach adenocarcinoma. HR, Hazards Ratio, calculated based on Cox PH Model.

f. Overall survival (OS) of patients diagnosed with malignancies and displaying insignificant correlation with PDK4 in cancer cells. BRCA, breast invasive carcinoma. HNSC, head-neck squamous cell carcinoma. LUSC, lung squamous cell carcinoma. HR, Hazards Ratio, calculated based on Cox PH Model.

Data in all bar plots are shown as mean ± S.D. and represent 3 biological replicates. *P* values were calculated by two-sided unpaired Student's *t*-tests (**c**), two-way ANOVA (**b**) or Log-rank (Mantel-Cox) tests (**d**, **e**, **f**). ^, $P > 0.05$. **, $P < 0.01$. ****, $P < 0.0001$.

Supplementary Fig. 2. Metabolic characterization of therapy-induced senescence.

a. Partial metabolic profiling (glycolysis) of senescent cells induced by mitoxantrone (MIT) and incubated with uniformly labeled [U-$^{13}C_6$]-glucose.

Results from GC-MS analysis of metabolites as indicated.

b. Partial metabolic profiling (TCA cycle) of senescent cells induced by MIT. Results from GC-MS analysis of metabolites as indicated. TCA, tricarboxylic acid cycle.

c. Partial metabolic profiling (glycolysis) of senescent cells induced by ionizing radiation (RAD). Results from GC-MS analysis of metabolites as indicated.

d. Partial metabolic profiling (TCA cycle) of senescent cells induced by RAD. Results from GC-MS analysis of metabolites as indicated.

e. Partial metabolic profiling (glycolysis) of stromal cells exposed to carbonyl cyanide 4-(trifluoromethoxy) phenylhydrazone (FCCP) and incubated with uniformly labeled [U-$^{13}C_6$]-glucose. Results from GC-MS analysis of metabolites as indicated.

f. Partial metabolic profiling (TCA cycle) of senescent cells exposed to FCCP. Results from GC-MS analysis of metabolites as indicated.

g. Quantitative measurement of PDK4 transcript expression after exposure of PSC27 cells to individual treatment as indicated. Cell lysates collected for assessment 7 d after completion of *in vitro* treatment. Signals normalized to CTRL. MIT, mitoxantrone. RAD, gamma radiation. FCCP, carbonyl cyanide 4-(trifluoromethoxy) phenylhydrazone. Data are shown as mean ± S.D. in the scatter dot blot.

h. Representative TEM images showing detailed ultrastructural profile of mitochondria in PSC27. L, low resolution (upper). H, high resolution (lower). Areas in rectangles of upper images zoomed into lower images. Arrowheads, mitochondria. Scale bars, 1.0 μm.

i. Comparative quantification of mitochondrial number. Data presented as the average number of mitochondria *per* field determined for each cell group.

j. Evaluation of mitochondrial mass. Cells subject to fluorescence staining with 500 nM MitoTracker™. Analysis performed on a Cytation 3 Cell Imaging Multi-Mode Reader, with signals normalized to MFI *per* cell field. MFI, MitoTracker fluorescence intensity.

Data in all bar and dot plots are shown as the mean ± S.D. and represent 3 (**g**) or 3-6 (**a**-**f**, **i**-**j**) biological replicates. *P* values were calculated by two-sided unpaired Student's *t*-test (**a**-**g**, **i**-**j**). ^, $P > 0.05$. *, $P < 0.05$. **, $P < 0.01$. ***, $P < 0.001$. ****, $P < 0.0001$.

Supplementary Fig. 3. Metabolic profiling of human stromal cell lines upon genotoxicity-induced senescence.

a. Partial metabolic profiling (glycolysis) of senescent cells (HFL1) induced by BLEO (TIS) and incubated with uniformly labeled [U-$^{13}C_6$]-glucose. Results from GC-MS analysis of metabolites including DHAP, GAP, 3PG, pyruvate and lactate are shown.
b. Partial metabolic profiling (TCA cycle) of senescent cells (HFL1) induced by

BLEO (TIS) and incubated with uniformly labeled [U-$^{13}C_6$]-glucose. Results from GC-MS analysis of metabolites including citrate, α-ketoglutarate, succinate, fumarate and malate, are presented. TCA, tricarboxylic acid cycle.

c. Measurement of extracellular fluids collected from HFL1 with XF24 extracellular flux analyzer. Pyruvate and lactate were assayed as indicated.
d. Measurement of ATP production by HFL1 cells. ATP production was measured as (last rate measurement before oligomycin injection) minus (minimum rate measurement after oligomycin injection).
e. Partial metabolic profiling (glycolysis) of senescent cells (HBF1203) induced by BLEO (TIS) and incubated with uniformly labeled [U-$^{13}C_6$]-glucose. Results from GC-MS analysis of metabolites including DHAP, GAP, 3PG, pyruvate and lactate are shown.
f. Partial metabolic profiling (TCA cycle) of senescent cells (HBF1203) induced by BLEO (TIS) and incubated with uniformly labeled [U-$^{13}C_6$]-glucose. Results from GC-MS analysis of metabolites including citrate, α-ketoglutarate, succinate, fumarate and malate, are presented.
g. Measurement of extracellular fluids collected from HBF1203 with XF24 extracellular flux analyzer. Pyruvate and lactate were assayed as indicated.
h. Measurement of ATP production by HBF1203 cells. ATP production was measured as (last rate measurement before oligomycin injection) minus (minimum rate measurement after oligomycin injection).

Data in all bar plots are shown as mean ± S.D. and represent 3 (**b**, **c**, **f**, **g**) or 3-5 (**a**, **d**, **e**, **h**) biological replicates. *P* values were calculated by two-sided unpaired Student's *t*-tests for all datasets. ^, $P > 0.05$. *, $P < 0.05$. **, $P < 0.01$. ***, $P < 0.001$. ****, $P < 0.0001$.

Supplementary Fig. 4. Measurement of tumor growth in the course of preclinical trials.

a. Tumor growth surveillance in the time course of preclinical therapies. Measurement started from the 18th day after *in vivo* xenografting of tissue recombinants until the end of each therapeutic regimen, at a frequency of once *per* 3 days.

Data are shown as mean ± S.D. and represent 3 biological replicates. MIT, mitoxantrone. *P* values were calculated by two-sided unpaired Student's *t*-tests. **, $P < 0.01$. ****, $P < 0.0001$.

Supplementary Fig. 5. Expression profile characterization of the SASP and senescence-associated genes in human prostate cancer cells overexpressing ectopic PDK4 and subject to senescence induction.

a. Immunoblot analysis of PDK4 expression after transduction of a human PDK4 construct to PC3 and DU145 lines, respectively. GAPDH, loading control.
b. Quantitative RT-PCR assessment of the expression of typical SASP factors and

senescence markers including $p16^{INK4a}$ and $p21^{CIP1}$ in PC3 cells. Cancer cells were treated in culture as indicated.

c. Quantitative RT-PCR assessment of the expression of typical SASP factors and senescence markers including $p16^{INK4a}$ and $p21^{CIP1}$ in DU145 cells. Cancer cells were treated in culture as indicated.

Data in all bar plots are shown as mean ± S.D. and represent 3 biological replicates. *P* values were calculated by two-sided unpaired Student's *t*-tests (**b**-**c**). ^, $P > 0.05$. *, $P < 0.05$. **, $P < 0.01$.

Supplementary Fig. 6. Measurement of tumor growth and potential impact of drug-delivered cytotoxicity *in vivo* by pathophysiological appraisal.

a. Body weight determination performed on a weekly basis for immunodeficient mice carrying PCa tumors and receiving the treatment of MIT and/or PDK4-IN.

b. Serum measurement of creatinine, urea, alkaline phosphatase (ALP), and alanine aminotransferase (ALT) with terminal bleeds (cardiac punctures) taken at the end of therapeutic regimens for mice depicted in **a**.

c. Body weight determination performed on a weekly basis for mice carrying BCa tumors and receiving the treatment by DOX and/or PDK4-IN.

d. Serum measurement of creatinine, urea, alkaline phosphatase (ALP), and alanine aminotransferase (ALT) with terminal bleeds (cardiac punctures) taken at the end of therapeutic regimens for mice described in **c**.

Data in all graphs (**a**-**d**) are shown as mean ± S.D., $n = 3$ *per* group. MIT, mitoxantrone. DOX, doxorubicin. All *P* values were calculated by one-way ANOVA (**a**-**d**). ^, $P > 0.05$.

Supplementary Fig. 7. Assessment of potential impact of drug-delivered cytotoxicity including its influence on the immune system of wildtype animals.

a. Body weight determination performed on a weekly basis for immunocompetent animals (C57BL/6J background) receiving treatment by MIT and/or PDK4-IN.

b. Serum measurement of creatinine, urea, alkaline phosphatase (ALP), and alanine aminotransferase (ALT) with terminal bleeds (cardiac punctures) taken at the end of therapeutic regimens for mice described in **a**.

c. Routine analysis of peripheral blood. The circulating levels of hemoglobin, WBCs, lymphocytes and neutrophils at the end of each therapeutic regimen were determined.

d. Body weight determination performed on a weekly basis for immunocompetent animals (C57BL/6J background) receiving treatment by DOX and/or PDK4-IN.

e. Serum measurement of creatinine, urea, alkaline phosphatase (ALP), and alanine aminotransferase (ALT) with terminal bleeds (cardiac punctures) taken

at the end of therapeutic regimens for mice described in **d**.

f. Routine analysis of peripheral blood. The circulating levels of hemoglobin, WBCs, lymphocytes and neutrophils at the end of each therapeutic regimen were determined.

Data in all graphs (**a**-**f**) are shown as mean ± S.D., *n* = 3 *per* group. MIT, mitoxantrone. DOX, doxorubicin. WBC, white blood cell. All *P* values were calculated by one-way ANOVA (**a**-**f**). ^, *P* > 0.05.

Supplementary Fig. 8. Molecular profiling of human NOX family member expression and influence of ATP synthesis interference on senescent cells.

a. Quantitative measurement of transcript expression of human NOX family members. Proliferating (CTRL) or senescent (SEN) PSC27 cells were exposed to vehicle or chemical lactate (10 μM) in culture, with cells lysed 3 days afterwards for expression assays. Signals normalized to vehicle-treated sample *per* factor.

b. Comparison of mitochondrial superoxide levels with the indicator MitoSOX. Cells transduced with a control (vector) or human Parkin construct (PRKN), treated by control (CTRL) or bleomycin (BLEO), before exposed to DMSO or PDK4-IN (IN).

c. Determination of SASP factor production by ELISA. Media conditioned by PSC27 was assayed for the amount of typical SASP factors at protein level and normalized as units of ng/ml/10^4 cells.

d. Measurement of SASP factor production by ELISA. Media experimentally assessed in the way similar to **c**, except that Gboxin, a specific inhibitor of OXPHOS and suppressor of F_0F_1 ATP synthase activity, was used to treat cells before senescence.

e. Assessment of mitochondrial superoxide levels with MitoSOX. Cells were subject to process in a manner resembling **b**, except that the chemical Gboxin was employed to treat cells.

f. Appraisal of ATP production by PSC27. Data derived from an XF24 extracellular flux analyzer and presented as units of pmol/min.

g. Analysis of ATP production level with an ATP assay kit. Data derived from readings of a luminometer and normalized as units of μmol/μg cell lysate.

h. Representative images of cells after SA-β-staining in culture. Cells subject to SA-β-staining after processed in the manner resembling **d**. Scale bar, 20 μm.

i. Comparative statistics of SA-β-staining positivity. Cells treated as described in **d**, with SA-β-staining positivity measured and compared between experimental groups.

Data in all bar plots are shown as mean ± S.D. and represent 3 biological replicates. *P* values were calculated by two-sided unpaired Student's *t*-tests (**a**, **b**, **c**, **d**, **e**, **f**, **g**, **i**). ^, *P* > 0.05. *, *P* < 0.05. **, *P* < 0.01. ***, *P* < 0.001. ****, *P* < 0.0001.

Supplementary Table 1. Top list of genes upregulated in PCa cells upon treatment with media conditioned from PDK4-overexpressing stromal cells

Ranking no.	Cell lines		
	PC3	DU145	M12
1	*ADAMTS9*	*HMGB1P3*	*KRTAP2-3*
2	*GABRA3*	*ADAM1A*	*MAP6D1*
3	*HS3ST3A1*	*AC027796.3*	*AC107068.1*
4	*BCAT1*	*XIST*	*TSNAX-DISC1*
5	*SLCO3A1*	*AC068989.1*	*AC125807.2*
6	*PLXDC2*	*AL161665.1*	*AC004706.3*
7	*GALNT13*	*AC083843.3*	*AL451136.1*
8	*RUNX1T1*	*RORB*	*AC025165.6*
9	*ANO3*	*AC004707.1*	*CTAGE8*
10	*PTCHD1*	*C1QTNF3-AMACR*	*AC010463.1*
11	*GPR27*	*AC090971.2*	*BX470111.1*
12	*AIM2*	*RSAD2*	*IMPDH1P10*
13	*IGFL2-AS1*	*DIO2*	*ZNF625-ZNF20*
14	*AL137129.1*	*AC025442.2*	*AC025165.2*
15	*ZNF667-AS1*	*AC074143.1*	*ZNF883*
16	*AP001267.5*	*ZDHHC4P1*	*AC026464.4*
17	*GAS1*	*IFNB1*	*AC010132.2*
18	*ZNF667*	*SCN3A*	*AC060766.5*
19	*TENM3-AS1*	*SPTA1*	*AC120057.2*
20	*AL049840.5*	*AC001226.2*	*CSNK2A3*
21	*SLC2A3P1*	*AP001486.2*	*AC092143.2*
22	*FGFR2*	*AL049795.2*	*PSG9*
23	*MGAM*	*AL390195.1*	*AC092143.3*
24	*ZFP92*	*AC010422.6*	*AC034229.2*
25	*MAGI2-AS3*	*TMEM183B*	*AC022613.3*
26	*AL133318.1*	*H2AC7*	*AC113189.2*
27	*HIF1A-AS1*	*ARLNC1*	*SLC9A3*

28	*HS3ST3B1*	*MORC2-AS1*	*AC096586.1*
29	*AL034430.1*	*AC027601.3*	*HNRNPA3P9*
30	*DNAAF4-CCPG1*	*AC231657.3*	*AL450467.1*
31	*AL135818.2*	*CH25H*	*AC123768.4*
32	*OLR1*	*LINC02235*	*AP000894.4*
33	*RSPO3*	*AC113348.2*	*LINC00327*
34	*LIMS4*	*IMPG2*	*STRA6*
35	*ADAM28*	*AC016394.2*	*AL645941.2*
36	*EVI2B*	*SAMD9L*	***AC069257.3***
37	*FMN2*	*LRRC39*	*HES5*
38	*AC090877.2*	*AC137932.2*	*GRIP2*
39	*GPR141*	*LINC01252*	*AC007686.3*
40	*RAB43P1*	*AC008522.1*	*AL136988.1*
41	*CHRDL1*	*AC120057.2*	*CFAP299*
42	*CST7*	*KCNT2*	*IGFBP3*
43	*AC011446.1*	*H3C6*	*PEAR1*
44	*LINC00941*	*AC103591.4*	*AC092032.2*
45	*CA2*	*PPM1K-DT*	*EXOSC6*
46	*CITED1*	*AL132639.3*	*NAV2-AS1*
47	*EDARADD*	*LRTM2*	*FGB*
48	*AC093627.1*	*AC107071.1*	*AL049795.2*
49	*COLCA2*	*IFIT2*	*AC020663.3*
50	*FDCSP*	*AC138866.1*	*NLRP3*
51	*MCTP2*	*AC069234.3*	*RPSAP53*
52	*AC018628.2*	*AC099521.3*	*AC087821.2*
53	*AC037471.2*	*AC044802.1*	*AC092675.1*
54	*AC087072.1*	*CICP6*	*BMP8B-AS1*
55	*AL451136.1*	*MUC17*	*AL663070.1*
56	*GABRG1*	*BX324167.2*	*TBC1D22A-AS1*
57	*FAT3*	*AL109811.2*	*AC099850.2*
58	*AC036108.2*	*TRIM22*	*INE2*
59	*HAND2-AS1*	*CAGE1*	*AC116366.1*
60	*BEX1*	*CXCL10*	*BCL11B*
61	*IGSF1*	*CTBP2P10*	*AF235103.3*

62 *ALDH1A1*	*CTBP2P9*	*AC009268.2*
63 *ANKRD20A2P*	*C2orf66*	*LRRN3*
64 *SLC44A5*	*AC005832.4*	*POU5F1P5*
65 *ABCA12*	*AC012531.3*	*AC011317.1*
66 *AL499627.1*	*AL049869.3*	*PPIAP41*
67 *ACADL*	*AC025048.6*	*AC104446.2*
68 *AC007068.1*	***HTR2B****	*RPSAP36*
69 *SPRR2E*	*GRIK1-AS1*	*RPL7AP10*
70 *AC069234.2*	*AC011379.2*	*KCNJ13*
71 *AL031600.3*	*AC009054.2*	*RN7SL4P*
72 *ETNPPL*	*AC124319.1*	*SFT2D3*
73 *TRMT9B*	*ZIK1*	*AC015871.8*
74 *AC008012.1*	*AC004980.2*	*LINC02721*
75 *NKX1-2*	*ADAM20P1*	*AC008012.1*
76 *GDNF*	*CLCA4-AS1*	*NPTX1*
77 *COL25A1*	*AC025475.2*	*AC096536.2*
78 *FOLH1*	*TRAPPC3L*	*AC126335.2*
79 *MDGA2*	*DEPDC1-AS1*	*AC022150.4*
80 *TMEM47*	*CDRT15*	*MEF2B*
81 *FDPSP7*	*JAM3*	*HCG15*
82 *AC005224.3*	*AP001610.2*	*MCUR1P1*
83 *PTGER3*	*AC005899.4*	*AL358613.3*
84 *AC100861.2*	*AC106793.1*	*GRTP1-AS1*
85 *AC092118.2*	*MIR2052HG*	*MID1IP1-AS1*
86 *PPARGC1A*	*AC061975.8*	*AC008786.1*
87 *ANGPT2*	*AC068620.3*	*AL645504.1*
88 *AC092802.2*	*TRPC6*	*AL133415.1*
89 *AC060814.2*	*LINC00547*	*AC117500.2*
90 *AC078860.2*	*AC105339.4*	*DEFB134*
91 *AC022150.4*	*ITGB6*	*FAM90A2P*
92 *NLGN4X*	*AC000068.3*	*NOS2*
93 *AP001486.2*	*AC009070.1*	*AC055840.1*
94 *AFF2*	*ZNF625-ZNF20*	*AC019193.2*
95 *CTSE*	*LINC00452*	*KCNV1*

96	*PPP4R4*	*FP671120.4*	*SPDYE12P*
97	*AP003783.1*	*AC026740.1*	*HTRA4*
98	*LINC00345*	*AC011816.1*	*SCARNA10*
99	*TCEA1P2*	*AC091060.1*	*AC002044.2*
100	*PSG8*	*IFNL3*	*AC003957.1*
101	*PTPN22*	*AC233992.3*	*GAPDHP63*
102	*S100A12*	*AC104446.2*	*AC139530.3*
103	*EGFL7*	*AC092153.1*	*AC020907.4*
104	*PNLIPRP3*	*AL357079.2*	*RPRML*
105	*LONRF2*	*FAM133DP*	*H4C1*
106	*AC116407.4*	*AC007036.5*	*H4C11*
107	*ROR2*	*XIRP2*	*AC068987.3*
108	*SLC19A3*	*KBTBD13*	*TPBGL*
109	*GPR85*	*DPY19L2P1*	*NIM1K*
110	*PSMD6-AS2*	*AC064801.2*	*PEG13*
111	*H1-4*	*MSTN*	*CATSPERZ*
112	*NPY*	*AC103740.2*	*AC135782.3*
113	*SLC2A3*	*FAM230B*	***HTR2B****
114	*AP001350.2*	*AL031055.1*	*ESM1*
115	*AC006001.4*	*PNLDC1*	*RASL10A*
116	*DAPK1*	*NLRP11*	*AL117379.1*
117	*LINC02085*	*GABRA2*	*DUSP2*
118	*AC113346.1*	*OR7E19P*	*NAIPP1*
119	*AC090236.2*	*C10orf143*	*GKN2*
120	*HNRNPCP2*	*AC104837.2*	*TSPAN32*
121	*FAR2P1*	*KCNE2*	*SNX18P4*
122	*AL357992.1*	*PPP1R36*	*NPM1P24*
123	*SLAMF7*	*TMEM225B*	*SLC25A1P5*
124	*AC139272.1*	*TUBA3D*	*AL391069.1*
125	*ELMO1*	*CFP*	*PA2G4P6*
126	*ZNF790*	*IFIT3*	*AL049757.1*
127	*LRRC4*	*AC107027.1*	*AC007161.3*
128	*LINC02026*	*AL445183.2*	***AC026316.4***
129	*CT75*	*TNFSF10*	*AC104472.2*

130	*BBOX1*	*AL356776.2*	*AC211476.5*
131	*AC024560.5*	*VEGFD*	*AC004854.2*
132	*LINC02631*	*AC008758.1*	*AC005831.1*
133	*AL137847.2*	*AC108863.1*	*KRT8P36*
134	*AC079336.2*	*AL929602.1*	*ID2*
135	*LY9*	*SMG7-AS1*	*TSSK4*
136	*SGCZ*	*AC013652.1*	*OMG*
137	*CNR2*	*LINC02795*	*AL109614.1*
138	*IL6*	*CCDC73*	*RDH10-AS1*
139	*AC025809.2*	*AL158835.1*	*TPTE2P2*
140	*MUC12*	*LINCR-0001*	*LINC01993*
141	*TMED10P1*	*CCL4L2*	*AL392183.1*
142	*TMEM74*	*AC090286.3*	*MIR4482*
143	*LDB2*	*LEFTY1*	*SEPTIN7P1*
144	*AC018512.1*	***AL132656.3***	*LNC-LBCS*
145	*SLC6A13*	*AUXG01000058.1*	*AOX2P*
146	*RGPD1*	*AP006565.1*	*AC017028.1*
147	*AL445685.3*	*AC023310.2*	*ITGA4*
148	*LIN7A*	*AC008567.3*	*HIF1A-AS1*
149	*KIF1A*	*LINC01914*	*AKAP17BP*
150	*SLC28A3*	*IL5*	*GAPDHP40*
151	*IL20*	*AC093668.1*	*AL133375.1*
152	*NEK3*	*GRAMD4P7*	*AL136115.1*
153	*AC026464.4*	*EPHX3*	*ODAPH*
154	*CAPN6*	*KHDC1L*	*CLDN14*
155	*CTTNBP2*	*LINC02211*	*PAPOLB*
156	*AC006441.3*	*AC100827.4*	*AC090527.3*
157	*AC073508.2*	*AC004223.3*	*AL162231.3*
158	*AL627309.3*	*RBBP4P2*	*AC004865.2*
159	*TRIM6-TRIM34*	*AC091564.2*	*LINC02028*
160	***HTR2B****	*CICP3*	*AC245884.11*
161	*FAM71A*	*KCNS1*	*AL353729.1*
162	*LINC00540*	*MIR194-2HG*	*STK19B*
163	*AP000919.1*	*AC120024.1*	*LINC00628*

164 *AC068282.1*	*AC018804.1*	*AC037198.2*
165 *AURKAP1*	*CACNA2D4*	*CHRDL2*
166 *AL158212.2*	*EEF1DP2*	*P2RY12*
167 *PRKAG2-AS1*	*MYO16-AS1*	*AC099050.1*
168 *AC091057.7*	*AL353579.1*	*AC022079.2*
169 *GJB6*	*AC007686.3*	*AL096817.1*
170 *AC008147.2*	*TMED10P1*	*AC139426.1*
171 *AC026367.1*	*AC007728.2*	*AL161670.2*
172 *AC100788.2*	*AL136172.1*	*RN7SL466P*
173 *AC055811.2*	*CMPK2*	*AP001020.1*
174 *ARHGAP20*	*CDRT1*	*SPDYE8*
175 *LMOD3*	*PLAAT2*	*SLC9B1*
176 *AL133325.3*	*RRH*	*AKR7A2P1*
177 *AC022613.3*	*AC104472.3*	*UTS2B*
178 *EPHA7*	*AC034193.1*	*NSG1*
179 ***FGD5***	*AL589743.1*	*EXOSC3P1*
180 *SLC7A13*	*AL359258.2*	*AP001781.1*
181 *GJB7*	*AP002373.1*	*AC026333.4*
182 *AL445493.2*	*AC011330.3*	*AC004241.4*
183 *KNOP1P2*	*MEGF10*	*Z83844.1*
184 *AF287957.1*	*PELATON*	*TBC1D3F*
185 *SGPP2*	*ZNF474*	*AC117503.5*
186 *AGGF1P3*	*PKN2-AS1*	*AL121694.1*
187 *CARS1-AS1*	*MARK2P9*	*AC108751.4*
188 *AL033397.1*	*AC005324.5*	*AC112512.1*
189 *PRLR*	*CPO*	*AC100861.2*
190 *C2CD6*	*LINC00112*	*AC018755.1*
191 *PLA2G4A*	*LIPJ*	*AL139156.2*
192 *HIF1A-AS3*	*ARHGAP29-AS1*	*OR7E12P*
193 *GABRQ*	*AL390066.2*	*RBM17P4*
194 *RPL23AP60*	*PDGFD*	*MT1E*
195 *AC107068.1*	*AC005722.3*	*AC009095.1*
196 *AC096541.1*	*CCL8*	*AC027307.1*
197 *GKN2*	*AC091100.1*	*HM13-AS1*

198 *AC114947.2*	*PINX1*	*AP002449.1*
199 *CA3-AS1*	*SLC34A2*	*SLCO1B3-SLCO1B7*
200 *TRIML2*	*AC009902.3*	*AC012513.1*
201 *PRO1804*	*XBP1P1*	*AC079598.3*
202 *ADRA2C*	*GNG13*	*AC012354.9*
203 *RPL12P16*	*FREM1*	*FAM181A-AS1*
204 *PTGS2*	*CX3CR1*	*LRRC14B*
205 *AC012414.5*	*LINC00968*	*TRIM60P18*
206 *FAM90A12P*	*ITGB1BP2*	*AC016027.5*
207 *AP000873.3*	*AP000365.1*	*ANXA8*
208 *FTH1P23*	*TMEM31*	*PSG4*
209 *LINC02416*	*AP000942.5*	*VSTM1*
210 *RPS15AP10*	*AC078850.1*	*CCDC184*
211 *WNT6*	*AC004982.1*	*SLX1B*
212 *AC007546.1*	*ACOD1*	*AL133406.2*
213 *SEC61G-DT*	*USP30-AS1*	*AP000577.2*
214 *AC048344.4*	*AC090229.1*	*AL365223.1*
215 *SMC2-AS1*	*KRT17P1*	*VN1R81P*
216 *SCARNA12*	*ANGPT1*	*IVL*
217 *INSYN2A*	*AC009019.1*	*PLPP7*
218 *DLGAP1*	*LIPI*	*AC105020.4*
219 *AP001625.2*	*AC007938.3*	*AL450405.1*
220 *SLC25A2*	*BAAT*	*VN1R5*
221 *NMUR2*	*FBXW10*	*AL355312.4*
222 *DYRK3-AS1*	*AL136295.17*	*HMGN2P5*
223 *AC005828.3*	*RFPL4A*	*MGC4859*
224 *CYS1*	*EPGN*	*KRT8P22*
225 *MAP3K19*	*MX2*	*RN7SL395P*
226 *AC009269.4*	*UCP3*	*AC084782.1*
227 *LINC01068*	*IFNL2*	*LINC01562*
228 *GPR101*	*AC048341.1*	*SETP5*
229 *AC106779.1*	*LINC01504*	*CCDC144NL*
230 *DDX50P2*	*RTP4*	*PPIAP6*
231 *NCF1C*	*WEE2-AS1*	*HSPE1P4*

232	*FAM183BP*	*POSTN*	*EIF3FP3*
233	*FAM209A*	*AC092143.1*	*TSSK1B*
234	*AC110995.1*	*AC106886.2*	*SMG7-AS1*
235	*AC126177.6*	*AC110275.1*	*AC130456.5*
236	*AF111169.3*	*AL645941.1*	*RNU4-25P*
237	*DRD1*	*TECRP1*	*AC093023.1*
238	*AC009095.1*	*PNPT1P1*	*AC055872.2*
239	*AC073052.1*	*LINC01511*	*AP001269.4*
240	*RSPO2*	*AC019117.3*	*AC007272.1*
241	*AC008734.1*	*HERC2P7*	*AC023424.2*
242	*ENO1P1*	*AL160394.2*	*AL353729.2*
243	*AC010735.1*	*GPR176-DT*	*AC005609.2*
244	*CTRB1*	*AC005914.1*	*AC022137.2*
245	*SCN1A*	*ANKRD26P1*	*AC245748.2*
246	*AL583843.1*	*AL359198.1*	*TTC21B-AS1*
247	*TNFSF18*	*UPK2*	*ZBTB20-AS5*
248	*F2RL2*	*ABCA9*	*AP000879.1*
249	*NR4A3*	*TACR2*	*NSA2P7*
250	*LGR5*	*AC010680.1*	*AC242628.1*
251	*AC002480.2*	*RPL23AP81*	*AP001636.2*
252	*ELMOD1*	*TECRL*	*AC100793.4*
253	*BATF3*	*AL138966.2*	*LRRC71*
254	*AC025031.2*	*AL161662.1*	*FMO6P*
255	*AKR1D1*	*SNORA71B*	*AC005521.1*
256	*LGI2*	*NMRAL2P*	*AL133153.2*
257	*COL4A4*	*AL096870.8*	*NANOG*
258	*COL4A3*	*KLRG1*	*TNF*
259	*ITGA4*	*SPCS2P4*	*AC008147.4*
260	*AP006333.1*	*OR7E29P*	*DYRK3-AS1*
261	*AC245096.1*	*KCNJ9*	*PNPT1P1*
262	*AC133540.1*	*EIF4EP1*	*AC114284.1*
263	*AC027796.3*	*DPYSL5*	*SEMA5B*
264	*AC068647.2*	*FP671120.5*	*LINC00426*
265	*NCMAP-DT*	*FP236315.2*	*ARX*

266	*NMRAL2P*	*RFPL4B*	*AC008749.1*
267	*SLC22A3*	*AC027607.1*	*AL121809.1*
268	*AC069234.5*	*SLC6A1*	*AC068790.8*
269	*IER3-AS1*	*HSFY3P*	*AL365440.1*
270	*AC114760.2*	*CU634019.3*	*LINC01902*
271	*NUS1P2*	*RPL12P43*	*AP000866.3*
272	*HMGB3P14*	*ZNF80*	*AC092809.4*
273	*BX119927.1*	*GCSHP5*	*RN7SL145P*
274	*AC016866.1*	*AC008770.4*	*GPR1*
275	*CENPVL3*	*AL132857.1*	*AL031770.1*
276	*LINC00992*	*CD180*	*CNTFR*
277	*NPPC*	*SOAT2*	***GRIK1***
278	*NLRP10*	*AC021134.1*	*C1QL3*
279	*REELD1*	*RRS1-AS1*	*P2RY14*
280	*AP006587.2*	*LINC02293*	*AC011632.1*
281	*AC017071.1*	*AC114939.1*	*AL359918.2*
282	*CLEC12B*	*AC006946.1*	*CAMTA1-DT*
283	*FP236383.9*	*ATP4A*	*AC037486.1*
284	*WHSC1L2P*	*RN7SL834P*	*STX19*
285	*FOXF2*	*HLF*	*AC073343.1*
286	*AC020765.2*	*IGHEP2*	*RPS19P1*
287	*RPL28P2*	*NCOA4P2*	*AL353608.4*
288	*AC008731.1*	*KIAA0408*	*RPL11P4*
289	*AL050331.1*	*AC002463.1*	*AC022903.2*
290	*AQP4*	*AC068860.1*	*THY1*
291	*VWA8-AS1*	*GP5*	*AL355922.2*
292	*AC092135.3*	*CHCHD4P3*	*TUBBP6*
293	*BBOX1-AS1*	*AC124242.2*	*AL451069.3*
294	*GPR63*	*LINC00565*	*LBHD2*
295	*PPIAP45*	*RFTN2*	*AC139792.2*
296	*RAB7B*	*RASA4CP*	*YWHAZP2*
297	*AC007938.1*	***AC069257.3***	*SMIM18*
298	*AC093535.1*	*H2BC6*	*MINAR2*
299	*AC103591.3*	*ANAPC1P4*	*AL590440.1*

300 *GABRE*	*AC008870.3*	*AP000777.2*
301 *AC024598.1*	*AC008663.2*	***AL132656.3***
302 *HSPA8P15*	*LINC02068*	*AL772337.1*
303 *AC105393.1*	*SLC16A11*	*AL161443.1*
304 *RN7SL146P*	*CSAG3*	*ALPG*
305 *AL008725.1*	*RPL23AP20*	*AC010327.4*
306 *AC023590.1*	*SAMD9*	*SHISAL2B*
307 *CECR2*	*IRF4*	*AL049825.1*
308 *AC090409.1*	*NRIR*	*AL157834.1*
309 *AL445228.3*	*KRT17*	*AL354893.2*
310 *GFY*	*MYO1G*	*SCOCP1*
311 *AP001972.4*	*TNFSF13B*	*AL022341.2*
312 *G3BP1P1*	*CCDC168*	*ZNF385D*
313 *AC004832.6*	*PRAL*	*AC011284.1*
314 *PHBP5*	*FABP4*	*MRPL40P1*
315 *ZNF197-AS1*	*TAF7L*	*MYCL-AS1*
316 *RPL7AP65*	*FAM83E*	*AP001893.3*
317 *AL031595.1*	*P2RY8*	*AC009163.3*
318 *TNIP3*	*IFIT1*	*CR392039.5*
319 *HNF1A-AS1*	*LINC01554*	*AL008582.1*
320 *CPO*	*UGT3A2*	*EEF1E1P1*
321 *RRN3P4*	*LINC01882*	*AC138819.1*
322 *AC004895.1*	*IFIT1B*	*AC004967.1*
323 *BX649632.1*	*LDHAL6B*	*AL158068.1*
324 *FAP*	*DDX60L*	*AC023813.3*
325 *AC006427.2*	*CACNG8*	*GPR22*
326 *GRIN2B*	*HSPD1P4*	*AL022157.1*
327 *SOX2*	*LINC00562*	*MT1X*
328 *AC007342.6*	*AC022395.1*	*SLC2A3*
329 *LINC00996*	*AC068473.1*	*AL022332.1*
330 *FSIP2-AS1*	*XAF1*	*AL133353.1*
331 *FYB2*	*AC132153.1*	*AC015883.1*
332 *AC138430.1*	*PCDHA2*	*OTUD4P1*
333 *IL21R-AS1*	*AL391095.3*	*USP17L6P*

334 *PIP5K1B*	*AC063943.3*	*AP002812.1*
335 *AL445288.1*	*AL645608.1*	*CD44-AS1*
336 *AC022868.2*	*RPL7AP66*	*UBE2V1P1*
337 *PRELID3BP3*	*AL451166.1*	*AC020661.1*
338 *PDE3B*	*AC005785.2*	*AC005020.1*
339 *AC005682.1*	*LINC02798*	*AC245096.1*
340 *NIBAN1*	*DUXB*	*RPS3P2*
341 *VANGL2*	*AC073389.3*	*AC082651.3*
342 *PLEKHS1*	*AC025472.1*	*AC009055.2*
343 *FCMR*	*WBP11P2*	*LRRC37A15P*
344 *IL6-AS1*	*H2AC16*	*AL139243.1*
345 *NRG1*	*AL162615.1*	*RPL26P27*
346 *GCNT4*	*AC006017.1*	*AP000942.5*
347 *PMS2P10*	*AC004453.2*	*PSPC1-AS2*
348 *SOBP*	*LINC02377*	*HSPA8P3*
349 *SLC16A6P1*	*AC127459.1*	*HAUS1P1*
350 *AC104841.1*	*OAS2*	*SNRPCP19*
351 *AC073842.2*	*HCAR2*	*AP005018.2*
352 *GPC5*	*GOLGA8J*	*AL139351.3*
353 *SLC15A3*	*BMP5*	*AC008953.1*
354 *INSL4*	*RN7SL15P*	*AL356952.1*
355 *FGF18*	*AC097518.2*	*MAN2A1-DT*
356 *IL1A*	*RADIL*	*NXT1-AS1*
357 *AARD*	*LINC00487*	*AL138689.2*
358 *PALM3*	*AL158167.1*	*AC097493.3*
359 *LINC01140*	*CYP26C1*	*AC120042.2*
360 *MSI1*	*SLC25A47*	*BSNDP3*
361 *BNIP3P40*	*AL353746.1*	*AP001330.4*
362 ***AC069257.3***	*IRAG1*	*AP000897.1*
363 *AC110491.3*	*WFDC5*	*AC100821.2*
364 *AF196972.2*	*AL157871.2*	*CYP26C1*
365 *ASIC2*	*AP003072.5*	*TPPP3*
366 *EVPLL*	*MTAPP2*	*BLOC1S5-TXNDC5*
367 *AC106873.8*	*TM4SF4*	*AC005726.2*

368	*AL451007.2*	***LINC02617***	*LINC01398*
369	*AC091057.3*	*AC010809.3*	*AC005180.2*
370	*HSP90AB4P*	*IHO1*	*HMGB1P21*
371	*RHOXF2*	*AC008906.2*	*SNORD3B-1*
372	*LINC02133*	*AC090001.1*	*TAGLN*
373	*AC130324.1*	*CYP2C9*	*AC037487.1*
374	*AL355385.2*	*AC027309.1*	*RPL23AP97*
375	*AL023802.1*	*AC011317.1*	*AC139795.3*
376	*RPS3P6*	*AC073342.1*	*AL023803.3*
377	*DHX58*	*RAB1AP1*	*AC007956.2*
378	*DNAJC5B*	*LINC02301*	*AL391839.3*
379	*AC114744.2*	*OR2W6P*	*AL445228.3*
380	*AC074143.1*	*NUP210L*	*AL133500.1*
381	*AL590762.1*	*AL645504.1*	*WNT10B*
382	*EVI2A*	*PRLR*	*CU638689.1*
383	*MC5R*	*AL139811.1*	*AC131009.3*
384	*PKIA-AS1*	*LINC02809*	*AP003068.1*
385	*MMP3*	*RNF144A-AS1*	*ELFN1*
386	*PLSCR2*	*AC006557.5*	*MT2A*
387	*RCSD1*	*ARL4AP5*	*ID1*
388	*AC008687.1*	*AC026320.3*	*AC011603.3*
389	*LINC01309*	*ARHGAP15*	*AC004908.3*
390	*AC087239.1*	*AC011369.2*	*AP000311.1*
391	*AC079341.1*	*AC073130.1*	*AC117498.1*
392	*NANOG*	*PPIC-AS1*	*STXBP5L*
393	*SPATA18*	*AP001885.1*	*AL160171.1*
394	*LINC02269*	*H2BP9*	*AC032044.1*
395	*RPL21P89*	*AP001178.4*	*NCF2*
396	*AL645768.1*	*LMO2*	*AC244093.5*
397	*LINC02395*	*LINC00954*	*RPL12P4*
398	*AL513190.1*	*UBOX5-AS1*	*GSC*
399	*AL158835.1*	*IVL*	*LINC01711*
400	*AC099804.1*	*AL035413.2*	*AL731559.1*
401	*AC005165.3*	*RN7SL333P*	*C11orf91*

402	*FTH1P3*	*AMBP*	*CCL2*
403	*AL162386.2*	*ASDURF*	*SCARNA7*
404	*LINC02540*	*BX664718.2*	*AC114810.1*
405	*DRAXIN*	*AL513128.3*	*AC091057.7*
406	*AL118522.1*	*AL020995.1*	*AC034102.5*
407	*LINC02434*	*AP001619.2*	*AC008403.2*
408	*AC004840.1*	*CRYZL2P-SEC16B*	*SLC2A3P4*
409	*NLRP3P1*	*AL590399.3*	*SNX18P8*
410	*AC034154.1*	*LINC00102*	*AC092079.1*
411	*Z82215.1*	*AC138035.2*	*BLID*
412	*AC015819.1*	*BCRP3*	*PSG7*
413	*WNT10A*	*MIP*	*TUBG1P*
414	*AC074194.1*	*AL450992.1*	*AC068790.5*
415	*HERC2P4*	*AL589993.1*	*AC133540.1*
416	*AC133644.1*	*AC091891.1*	*LEF1-AS1*
417	*AC234772.2*	*AC090469.1*	*AL355990.2*
418	*AL033384.2*	*AP000962.2*	*POU2F3*
419	*GAPDHP2*	*SERPINC1*	*AL162615.1*
420	*AC114781.1*	*GBP5*	*ARPC3P1*
421	*AC010501.2*	*AC234781.1*	*ASAP1-IT2*
422	*AC122713.2*	*AC007325.1*	*SLC38A5*
423	*TAS2R64P*	*AC004224.2*	*HDAC11-AS1*
424	*SNRPGP15*	*AP000777.3*	*AC026124.2*
425	*RPL22P12*	*AC010401.1*	*CPSF1P1*
426	*AC004079.5*	*HSPE1-MOB4*	*DMWD*
427	*TAAR1*	*AL445490.1*	*GPR37L1*
428	*CHST13*	*TMEM151A*	*MIR155HG*
429	*AC107071.1*	*C1orf162*	*CCL20*
430	*AC024451.4*	*PLCB1*	*SERPIND1*
431	*AC012174.1*	*KCNK4*	*AC005837.4*
432	*RPS4XP8*	*H4C5*	*CSNK1A1P1*
433	*AL590235.1*	*IGFL1*	*AC022467.1*
434	*AL157400.4*	*SPTBN5*	*AC107463.1*
435	*ANKRD18EP*	*OASL*	*LINC01864*

436	*BAALC*	*EREG*	*GRIK2*
437	*C9orf106*	*AC115284.4*	*KRT18P20*
438	*CGB2*	*PLGLB2*	*AL662791.1*
439	*OR5H1*	*IFNE*	*ZBTB20-AS1*
440	*AL357054.1*	*AL078604.2*	*ARL4AP4*
441	*VINAC1P*	*DEF6*	*ZNF863P*
442	*AC005899.7*	*H3C14*	*RSL24D1P3*
443	*CDCA4P4*	*H3C15*	*DUTP2*
444	*ZNF829*	*EXOSC6*	*AC007780.1*
445	*AC254562.3*	*TPRG1*	*KIRREL1-IT1*
446	*SMIM15-AS1*	*RPL23AP24*	*AC022217.1*
447	*AC067968.1*	*AL356273.2*	*AL592437.2*
448	*PSG1*	*SNRPA1P1*	*AL390964.1*
449	*AC012501.3*	*TGFB2-OT1*	*AC109631.1*
450	*AC079601.2*	*SLFN14*	*LINC02568*
451	*OFD1P17*	*LINC00310*	*HSPD1P6*
452	*AMTN*	*AC011199.1*	*AC091185.1*
453	*CHST5*	*IFI44L*	*AC020978.8*
454	*AL137918.1*	*IFNL1*	*UBXN7-AS1*
455	*AC114402.1*	*PRUNE2*	*IPO7P2*
456	*NDUFA9P1*	*DDX58*	*TRPC5*
457	*AL354989.2*	*IDO1*	*AC073046.1*
458	*CHRND*	*AL445524.2*	*AL365357.1*
459	*TM4SF20*	*MYH7*	*AC124947.1*
460	*AL359916.1*	*AP3B2*	*FAM169B*
461	*AL021368.5*	*AC011487.1*	*MTCO1P31*
462	*AL355472.2*	*PRDM6*	*AP000648.9*
463	*AC022336.2*	*C20orf202*	*AC078852.2*
464	*HSPE1P3*	*AL356273.3*	*CRLF2*
465	*RPS4XP1*	*AC093843.1*	*AC115220.2*
466	*AL160191.1*	*AC073548.2*	*CFTR*
467	*RN7SKP51*	*NAV2-AS2*	*AC087683.3*
468	*AL033384.1*	*AC006978.2*	*AC234644.1*
469	*AC096543.2*	*AC092798.1*	*FP325317.2*

470	*DDX59-AS1*	*BRWD1-AS1*	*AC015911.8*
471	*RPL31P44*	*AL133338.2*	*KRT18P37*
472	*AC009955.2*	*AC239802.2*	*PCDHGA3*
473	*AC009034.1*	*AC243571.2*	*GUCA1B*
474	*HLX-AS1*	*AC046176.1*	*RIPPLY2*
475	*AC021739.3*	*AL137786.1*	*AC011497.1*
476	*PIGY-DT*	*AL592437.1*	*LINC00383*
477	*LINC00691*	*HNRNPA1P7*	*GRXCR2*
478	*AC073348.2*	*AC007036.3*	*FCN3*
479	*KCNC1*	*FLJ46284*	*AC008734.2*
480	*LINC01398*	*SMIM22*	*AL358216.1*
481	*AC004687.1*	*AC005392.2*	*TDRD9*
482	*AC004704.1*	*TBC1D3C*	*Metazoa_SRP*
483	*AP001107.2*	*CEACAMP4*	*AL079307.2*
484	*PRELID1P6*	***AC026316.4***	*ZMYND19P1*
485	*PPFIA2*	*AL358473.1*	*FAM71C*
486	*TAS1R2*	*AC025186.1*	*Z82206.1*
487	*ADAM33*	*PKD2L1*	*CITED1*
488	*IL36B*	*ASPN*	*SNRPCP3*
489	*AC008543.5*	*AL031777.2*	*RPSAP41*
490	*AC007336.1*	*RAET1L*	*ARHGAP22-IT1*
491	*RYR2*	*LSMEM1*	*AC073115.2*
492	*ANKRD20A4P*	*AC005014.2*	*DNAJB1P1*
493	*AL391280.2*	*BZW1-AS1*	*AC138430.1*
494	*ATP5PBP5*	*AC091516.1*	*AL023284.3*
495	*TRIM55*	*SLC45A2*	*AC004134.1*
496	*AC009220.3*	*AC068790.1*	*SVOPL*
497	*AC092428.1*	*NPY4R2*	*AP000777.3*
498	*AL512343.2*	*ZNF208*	*FSTL5*
499	*MT1JP*	*TEKT2*	*BNIP3P1*
500	*AL022318.5*	*TCP1P3*	*AC026336.2*
501	*AC005258.2*	*SIM1*	*AC004687.3*
502	*AC105339.3*	*AL135999.2*	*IFIT1B*
503	*AL441883.1*	*ZNF732*	*RPL7AP26*

504	*ANKRD34B*	*AC006141.1*	*AC004882.3*
505	*AC005281.1*	*M1AP*	*AL161912.4*
506	*STON1-GTF2A1L*	*C3orf49*	*KCTD9P1*
507	*AMY1B*	*AC068700.2*	*MT3*
508	*LINC02101*	*AC022121.1*	*PIP5K1B*
509	*CROCCP4*	*AL121583.1*	*HDGFP1*
510	*AGGF1P1*	*AL136419.2*	*ZSCAN5C*
511	*AC018545.1*	*AC008750.7*	*SNORA2B*
512	*LINC00355*	*CICP22*	*UBE2FP3*
513	*ZNF587P1*	*AL096711.2*	*RPL31P49*
514	*AC027338.2*	*RPL7P24*	*KCNT1*
515	*AL121758.1*	*P2RX2*	*AL355315.1*
516	*AC097065.1*	*GAGE2A*	*TPM3P7*
517	*FYTTD1P1*	*RAPSN*	*SLC25A6P5*
518	*AP001065.3*	*AC004967.1*	*AL096869.1*
519	*TREML3P*	*RPP38-DT*	*AC004232.1*
520	*AL035078.4*	*LINC01036*	*USP17L22*
521	*AC109454.1*	*SLC7A4*	*RPL6P2*
522	*AC006017.1*	*CXCL11*	*IL1RAPL2*
523	*OR7E121P*	*LINC00477*	*AC003035.1*
524	*AC069234.3*	*AL356804.1*	*IGHEP2*
525	*SNX18P10*	*MZB1*	*AC011509.3*
526	*NTRK2*	*APOC3*	*AC007106.2*
527	*AC002480.1*	*AL117378.1*	*OR6A2*
528	*FAM86B2*	*CASQ1*	*FAM197Y9*
529	*SLC30A3*	*AL121987.1*	*AC040963.1*
530	*STRA6*	*U62317.2*	*LINC01986*
531	*IL1R1*	*SPARCL1*	*AGBL4*
532	*KCNJ13*	*AC026956.2*	*EEF1B2P7*
533	*IGSF9B*	*LINC00158*	*RBPMS2P1*
534	*GBP1*	*AL136360.1*	*MYL2*
535	*Z83840.1*	*CASC18*	*TAF9BP1*
536	*AC093690.1*	*AC078795.3*	*SPCS2P4*
537	*ZNF345*	*ACE2*	*HNRNPMP1*

538	*AC138932.2*	*MIR122HG*	*LINC01132*
539	*AC037487.1*	*AC069499.2*	*HNRNPA1P35*
540	*SLC1A7*	*AL034374.1*	*AL929554.2*
541	*ANGPT1*	*AC015849.5*	*SNORA62*
542	*AC025171.4*	*AC244453.2*	*AC244258.1*
543	*HAL*	*ZNF300*	*TERT*
544	*C6orf58*	*TRBV12-4*	*PDE3A*
545	*AC012358.1*	*VDAC2P5*	*AC006213.7*
546	*CFH*	*DACT3-AS1*	*Z98885.1*
547	*AC079684.1*	*LCE5A*	*AC061975.6*
548	*IL24*	*AC079148.1*	*AC093214.1*
549	*MT1M*	*AL450467.1*	*IFIT1P1*
550	*TSTD1*	*ACTR3P2*	*AL139274.1*
551	*AC008083.2*	*GNA14*	*AC074134.1*
552	*DNAJC6*	*AL139022.1*	*OR10J1*
553	*LINC00536*	*AL161645.2*	*RN7SKP70*
554	*KYNU*	*LINC02228*	*Z99916.1*
555	*RUBCNL*	*AC100812.1*	*WWOX-AS1*
556	*WNT5B*	*SPACA4*	*AC022098.3*
557	*GRAMD2A*	*AC073111.3*	*PXDNL*
558	*PLA2G2F*	*FAM209A*	*AC008687.1*
559	*TSHZ3*	*AC009268.2*	*AL358232.2*
560	*PPP1R14C*	*AC104137.1*	*HOXC12*
561	*AHRR*	*GPR45*	*RPL5P25*
562	*GPA33*	*AC005831.1*	*AL138767.1*
563	*FKTN*	*PNPLA1*	*AD000090.1*
564	*AC002553.2*	*SPON1-AS1*	*RPL35P5*
565	*CFAP73*	*WNK3*	*LINC01018*
566	*AL590064.1*	*KRT18P34*	*AL606760.3*
567	*LINC00310*	*AL359921.2*	*AL139174.1*
568	*AC134407.3*	*RTL9*	*IRAG1-AS1*
569	*PCBP2-OT1*	*RFPL4AL1*	*POU5F1P4*
570	*SERPINA10*	*AC093525.1*	*LINC00475*
571	*BTG1*	*ZNF43*	*OLA1P2*

572	*SCG5*	*AC025048.2*	*LINC02832*
573	*SNAPC1*	*AC253536.3*	*AL513218.1*
574	*PRDM16*	*NLRP4*	*AL356234.1*
575	*AC093110.1*	*AC009560.4*	*MRGPRF*
576	*LHX1*	*AL138831.2*	*YRDCP2*
577	*LINC00466*	*AL353753.1*	*AC105460.2*
578	*PTMAP3*	*PPIAP42*	*THSD4-AS1*
579	*UBE2QL1*	*AC023794.5*	*FAM25A*
580	*AC117382.1*	*ERAS*	*TAGLN2P1*
581	*KRT18P12*	*ENPP7P11*	*C22orf15*
582	*FAM90A24P*	*AL020997.1*	*KLHDC8A*
583	*AC127496.6*	*GPBAR1*	*NPM1P5*
584	*AC092447.1*	*SLC28A2*	*AC009237.9*
585	*ACTG1P14*	*AC011773.3*	*AC099568.1*
586	*LINC01589*	*AXDND1*	*RPL23AP17*
587	*AC091564.2*	*HEPACAM2*	*AC004383.1*
588	*AL139339.1*	*APBA2*	*NRN1*
589	*Z69720.1*	*AC020663.2*	*SNX19P2*
590	*HSFX3*	*AC090159.1*	*AC122713.2*
591	*MEDAG*	*LINC00092*	*AC003005.2*
592	*AC106900.1*	*AC005776.1*	*AC004765.1*
593	*AC069282.1*	*AL110292.1*	*SLC8A3*
594	*DNAJC19P5*	*AL445435.1*	*AL122008.2*
595	*EEF1A1P7*	*NAGPA-AS1*	*AC009292.1*
596	*PAFAH1B3*	*AL096828.2*	*KRT75*
597	*AC132938.3*	*HTR5BP*	*NR0B2*
598	*C6orf47-AS1*	*AC010476.2*	*RN7SL753P*
599	*ANO1-AS1*	*AC009336.1*	*DDTP1*
600	*AL139286.1*	*HAUS1P2*	*RN7SL605P*
601	*AC092979.1*	*AC004918.1*	*TNRC18P2*
602	*FAM237B*	*AC073869.1*	*RTL5*
603	*HMGB1P27*	*AL589743.2*	*AC026100.1*
604	*SHISAL2B*	*AL731556.1*	*AC131888.1*
605	*AC011472.5*	*AC005034.2*	*AL592546.3*

606	*RPSAP12*	*AC026124.1*	*AC140168.1*
607	*AC068234.1*	*INTS4P1*	*AC002550.2*
608	*MTND5P15*	*AL354872.1*	*AL445248.1*
609	*LINC01511*	*GOLGA6L22*	*CELF2-AS2*
610	*AP004290.1*	*AC242376.1*	*IZUMO2*
611	*PAICSP4*	*AC026624.1*	*FP565171.1*
612	*AC004687.3*	*AC005041.4*	*MED28P7*
613	*RGS17P1*	*AL355377.3*	*AC116611.1*
614	*FDX1P1*	*KY*	*TRIM50*
615	*RPL13AP5*	*LDHAL6A*	*EEF1A1P9*
616	*AC087893.2*	*BCHE*	*ST8SIA2*
617	*KRT83*	*AP001267.3*	*AC008766.1*
618	*AL021707.8*	*AC034229.1*	*AP000889.1*
619	*AC112722.1*	*C2CD4D-AS1*	*SLC22A11*
620	*RPS3AP8*	*BX664615.2*	*GP5*
621	*NACA4P*	*AC004449.1*	*AP000902.1*
622	*HTR5BP*	*TRIM26BP*	*RPL3P1*
623	*FCRL5*	*CSNK1A1P1*	*AC106800.2*
624	*LINC02137*	*CHRNA1*	*RPL23AP55*
625	*CD34*	*MX1*	*AC008750.1*
626	*LINC00222*	*LINC01963*	*PGAM1P6*
627	*STARD6*	*LRIT3*	*LINC02515*
628	*AC244250.4*	*RPL23AP1*	*AP002884.3*
629	*TAF9BP1*	*SCRT1*	*AC023090.2*
630	*AC123788.1*	*RYR2*	*AP003969.2*
631	*SEPTIN14P12*	*AC006486.2*	*BRIX1P1*
632	*SHLD2P1*	*AC007106.1*	*AC012676.3*
633	*HKDC1*	*GPR75*	*AC142086.3*
634	*RAB5CP1*	*H2BC4*	*AL391058.1*
635	*TAS2R60*	*CHRNB2*	*AC019131.3*
636	*GPR174*	*AC015813.6*	*DEPDC1-AS1*
637	*LINC01118*	*AC092053.2*	*AC021106.2*
638	*AC131097.3*	*IGFBP7-AS1*	*FMR1-AS1*
639	*AC073257.2*	*CCL5*	*AL022098.1*

640	*GABRG3*	*PLAC8L1*	*MTRNR2L4*
641	*AL772337.3*	*LINC00942*	*OR7E100P*
642	*FGF13-AS1*	*USH2A*	*NANOS3*
643	*AL161645.1*	*AL138787.1*	*AC090159.1*
644	*AL359881.1*	*Z95118.2*	*USP17L7*
645	*AC020656.2*	*AC120057.3*	*RN7SL663P*
646	*AC022973.4*	*GRIN3A*	*AC008011.2*
647	*AC093799.1*	*B4GALT1-AS1*	*AL353718.1*
648	*AC080038.3*	*MYRFL*	*ADGRL2*
649	*AL031778.1*	*AC024560.1*	*FAM53B-AS1*
650	*AL049539.1*	*AC018644.1*	*AP002762.2*
651	*NLRP5*	*MT1E*	*H4C2*
652	*CABCOCO1*	*AC090617.10*	*AC097372.2*
653	*WFDC1*	*CAPSL*	*AC002540.1*
654	*AC093107.1*	*AC092143.2*	*AC007622.1*
655	*AC008073.3*	*U62317.1*	*AL139393.1*
656	*ST8SIA6*	*AC010809.2*	*RPL12P18*
657	*SASH3*	*KMO*	***LINC02617***
658	*FTH1P5*	*SPON1*	*AL137779.1*
659	*HSPA8P3*	*CU633906.5*	*AC084783.1*
660	*AC008537.2*	*BATF2*	*AL133477.2*
661	*AL136452.1*	*RIPOR2*	*CDY4P*
662	*THRSP*	*AC004801.2*	*CAPZA1P2*
663	*AC244093.2*	*PROZ*	*AC024257.4*
664	*RPL35AP32*	*GALR2*	*RN7SL502P*
665	*AL031666.3*	*AC010422.7*	*TAS2R8*
666	*AC005072.1*	*AL159169.2*	*AC145676.1*
667	*AC098818.2*	*CLDN6*	*AL450163.1*
668	*SLC9C2*	*AC037198.1*	*FFAR1*
669	*AC069234.4*	*AC093001.1*	*KLHL2P1*
670	*AC112178.1*	*AC140479.2*	*AL133368.1*
671	*AP006565.1*	*PAPPA-AS1*	*AL136520.1*
672	*AC010997.6*	*HAUS6P1*	*AL031666.1*
673	*CA7*	*OR10A2*	*RN7SKP118*

674 ***GRIK1***	*RBM22P2*	*AC027088.5*
675 *AL391987.3*	*AP000238.1*	*OMD*
676 *AC018647.1*	*AC087289.1*	*HNRNPA3P3*
677 *SLC35E1P1*	*AC027811.1*	*AC073326.1*
678 *AP002336.1*	*TBR1*	*CORT*
679 *AC002044.2*	*SEMA3D*	*AC114744.1*
680 *AC100821.2*	*GBP4*	*AC079336.4*
681 *Z98884.1*	*AL590282.1*	*PTMAP8*
682 *ZDHHC20-IT1*	*AC016745.2*	*AC106820.1*
683 *C4BPAP1*	*FRG2*	*LYPLAL1-AS1*
684 *LDHAP7*	*AC104109.2*	*AC016813.2*
685 *OR10A6*	*TP63*	*AC002451.2*
686 *MTND5P42*	*AC087392.5*	*HTR1DP1*
687 *PSG8-AS1*	*LINC00683*	*AC131934.1*
688 *AL591767.1*	*RSPH10B2*	*RN7SL225P*
689 *LINC01594*	*IFIH1*	*AL008638.5*
690 *AC008429.2*	*AC136297.1*	*SLC25A1P1*
691 *AC069257.1*	*FAM230E*	*TMEM71*
692 *AC011499.1*	*AC092881.1*	*MYLK2*
693 *ARHGAP26-AS1*	*AC110619.1*	*RAB43P1*
694 *KDM3AP1*	*ASB5*	*AP004609.3*
695 *GDNF-AS1*	*PI15*	*TEX38*
696 *AC093484.4*	*AC078795.1*	*STUB1P1*
697 *NPR1*	*GALNT4*	*SNORA11F*
698 *AC005104.1*	*IFI44*	*PIWIL3*
699 *LDLRAD4*	*HACD4*	*AC023389.2*
700 *C3orf80*	*AC004982.2*	*PRR26*
701 *DOCK10*	*AC037198.2*	*NDUFA9P1*
702 *EFCAB5*	*TLR3*	*GLRA4*
703 *AC011676.5*	*AC112907.3*	*OR2W6P*
704 *AL671986.1*	*GBP1P1*	*AADACP1*
705 *ZNF268*	*PEG10*	*CKS1BP2*
706 *PLA2G1B*	*AC068547.1*	*TOMM20L*
707 *INGX*	*PTPRQ*	*HMX2*

708	*CHRNB2*	*TNFRSF8*	*AC007688.2*
709	*CDHR1*	*DDX60*	*AC083967.1*
710	*DLEU7*	*AL360270.2*	*AC008915.2*
711	*DMD*	*AP000893.1*	*CTNNA1P1*
712	*CNTN2*	*AC090607.4*	*FGL2*
713	*AL356608.3*	*RPSAP6*	*AC006963.1*
714	*AL162713.2*	*AC019155.1*	*RPL7AP66*
715	*TMLHE-AS1*	*AC099811.6*	*AC061975.4*
716	*DUTP7*	*AL591893.1*	*Z95114.2*
717	*TGFB3-AS1*	*AC011447.6*	*RPL17P33*
718	*LNCTAM34A*	*AC005532.1*	*AC011477.8*
719	*AC007278.1*	*LINC02843*	*AC005593.1*
720	*AP001615.1*	*AL034417.3*	*PIP5K1P1*
721	*IL1F10*	*SEC23A-AS1*	*OR7E110P*
722	*MMP2*	*PCDHA9*	*BX679664.3*
723	*UTS2B*	*ZNF582*	*RPL7L1P2*
724	*AC023310.4*	*PDE1A*	*AC067945.2*
725	*AF196972.1*	*AL136295.3*	*PPIAP35*
726	*PGA3*	*AC104365.3*	*AC011491.2*
727	*AC079336.6*	*AL645922.1*	*AL031667.3*
728	*ROPN1*	*AC080079.2*	*AL358613.2*
729	***AL132656.3***	*CNNM3-DT*	*AP003469.2*
730	*AL109983.1*	*RNVU1-32*	*TRHDE*
731	*EPHX3*	*LINC02732*	*AL445237.1*
732	*MTND4P23*	*AC139495.1*	*AC124319.1*
733	*AC022395.1*	*LRRC37A15P*	*TREML1*
734	*CPNE4*	*AC117528.1*	*SDHCP3*
735	*CFAP161*	*FAM221B*	*OR2AG1*
736	*DPPA4*	*AC010260.1*	*AC099487.1*
737	*FLT3*	*LENEP*	*PAFAH1B1P1*
738	*NPM1P21*	*AC026333.3*	*AL592114.3*
739	*AP001781.1*	*AL121809.1*	*FTH1P1*
740	*PEF1-AS1*	*SAPCD1-AS1*	*LEMD1-AS1*
741	*AC034229.4*	*AC005786.4*	*AC083805.2*

742	*LINC02599*	*AC007637.1*	*BX072579.1*
743	*AC134669.1*	*AP000866.3*	*AC009236.2*
744	*AC016999.1*	*RPS27AP13*	*FRZB*
745	*RBMXP4*	*LINC00607*	*AC008781.1*
746	*AC006518.2*	*SNX18P8*	*RPS4XP5*
747	*AC073367.1*	*AL353705.2*	*AL162233.1*
748	*CDRT15*	*BLNK*	*AC114402.2*
749	*AC084724.1*	*AL133166.1*	*AC092756.1*
750	*OR11H7*	*FAM181A*	*GLYATL1P1*
751	*SV2B*	*OOSP1*	*RPL21P132*
752	*IL12A-AS1*	*AC016995.1*	*LINC01844*
753	*WFDC2*	*AL157902.2*	*AC006581.2*
754	*AC011284.1*	*AC139491.8*	*AC004594.1*
755	*FILIP1L*	*AC113414.1*	*AC092337.1*
756	*SOX3*	*AC104984.5*	*LINC01111*
757	*LINC00993*	*EFCAB8*	*CGB8*
758	*AC016687.2*	*AC009269.4*	*RPL5P3*
759	*AL607028.1*	*AC123912.3*	*AC004080.3*
760	*AC090809.1*	*ASCL1*	*SNORA71*
761	*AL356274.1*	*RN7SL23P*	*AC002075.2*
762	*AL079343.1*	*AL356274.1*	*AC026624.1*
763	*AC108134.3*	*AL023806.2*	*CES1P1*
764	*OR11H13P*	*LINC01096*	*PSG2*
765	*ZRANB2-AS1*	*AC000089.1*	*AC096631.2*
766	*VWA2*	*KNOP1P2*	*AC034114.2*
767	*OR2A3P*	*KRT18P52*	*AC079336.7*
768	*SYT2*	*MYOT*	*PHKBP1*
769	*KRT18P52*	*AC080128.2*	*SNAP23P1*
770	*AC112206.3*	*OR9A1P*	*AC009560.3*
771	*AL138999.1*	*AC027020.1*	*AP006621.4*
772	*AC107302.1*	*AC092724.1*	*AP001054.1*
773	*SLC25A34-AS1*	*AP001528.2*	*AC091951.1*
774	*AC010624.1*	*AC245452.1*	*LINC00928*
775	*AC145285.1*	*AC008972.1*	*PTTG4P*

776	*AC010889.2*	*SYT14P1*	*NFIA-AS2*
777	*LRRC38*	*RPL3P10*	*AC087276.3*
778	*RUNDC3B*	*AC006195.1*	*CCR6*
779	*ZNF569*	*AC020728.1*	*HBG2*
780	*AL513318.2*	*AP003419.2*	*AL080316.1*
781	*AL139407.1*	*KRT18P55*	*AL159163.1*
782	*AC090589.3*	*CCDC141*	*AL031591.1*
783	*AL121992.1*	*CDSN*	*NRADDP*
784	*AC007384.1*	*HSD17B3*	*DDX18P6*
785	*ISM1*	*AC111170.3*	*DCLRE1CP1*
786	*CILP2*	*TAFA1*	*IGF1*
787	*AL359636.2*	*CYP51A1-AS1*	*AC073610.1*
788	*AC139887.4*	*AC078788.2*	*AC011451.1*
789	*CU639417.4*	*RNASE1*	*RRAS2P1*
790	*TRPC4*	*AC090559.2*	*AC087071.2*
791	*AC146944.1*	*AC003101.2*	*RPS11P5*
792	*RPL21P12*	*AP000925.1*	*AL450263.2*
793	*ADGRB3*	*LRMDA*	*AC093899.2*
794	*MYH7*	*DIO3*	*PIGR*
795	*LY75-CD302*	*IL4*	*LINC01251*
796	*BMP2KL*	*TBX10*	*RPS26P8*
797	*OR2R1P*	*JCHAIN*	*AC008417.1*
798	*PTPRO*	*VCAM1*	*TNRC6C-AS1*
799	*ASTL*	*SLC25A2*	*AC069209.2*
800	*LINC00487*	*IDO2*	*PSMA6P1*
801	*SEPTIN7P9*	*ALG1L13P*	*AL589863.2*
802	*BEND3P1*	*Z98200.1*	*AC087257.1*
803	*AC134407.1*	*AC091185.1*	*U82671.1*
804	*AL354733.1*	*AC012213.2*	*AL357033.2*
805	*ZBTB44-DT*	*FCRL2*	*AL138963.1*
806	*SOHLH2*	*AL354861.3*	*POM121L6P*
807	*HMGA1P4*	*ASS1P12*	*RAB44*
808	*AC093567.1*	*RPL7P18*	*AC067930.3*
809	*TMEM26*	*AC010275.1*	*MAG*

810	*PLP1*	*AC008083.2*	*AC016027.3*
811	*GPR79*	*RPS5P2*	*AC027607.1*
812	*DUSP27*	*AC012254.4*	*AL356750.1*
813	*AL590068.3*	*RNF133*	*GNAT1*
814	*AC010998.2*	*ZPLD1*	*AL355581.1*
815	*RPL13AP6*	*KRT18P7*	*AC093802.1*
816	*AC004910.1*	*MTUS2-AS1*	*AL590133.1*
817	*NME8*	*GPR25*	*AC016245.1*
818	*AC004233.1*	*AC007686.4*	*AL356481.2*
819	*AL445363.2*	*RPS4XP10*	*AC107918.6*
820	*AC010999.2*	*CD209*	*AL356753.1*
821	*AL161729.3*	*AC006116.7*	*AC008686.1*
822	*AC113410.5*	*AC121247.1*	*EEF1A1P17*
823	*RSF1-IT2*	*TCF3P1*	*AMPD1*
824	*MTND4LP30*	*SFTPA2*	*AC010271.2*
825	*AC104461.1*	*AC023790.2*	*AC077690.1*
826	*RPL31P58*	*AC016245.2*	*RPL15P3*
827	*AC004882.3*	*PRMT1P1*	*RPL12P11*
828	*NR4A1AS*	*AL354710.2*	*CLCA4*
829	*AC139783.1*	*EHD4-AS1*	*AC010524.1*
830	*AP000790.1*	*AL157888.1*	*AC027288.1*
831	*CR559946.2*	*IL10*	*AL024508.1*
832	*MATK*	*AL592146.2*	*AC073869.1*
833	*C1QL3*	*LMF1-AS1*	*FAR2P4*
834	*AL357054.3*	*MACROD2*	*EIF1AXP2*
835	*FFAR2*	*CA12*	*AC005803.1*
836	*LINC02240*	*AC005746.1*	*AC111170.3*
837	*PTGIR*	*TAS2R30*	*AC016245.2*
838	*AC116651.1*	*AL355432.1*	*TGFBI*
839	***AC026316.4***	*AP000317.1*	*AL359198.1*
840	*PDZD9*	*XKR6*	*AC097634.4*
841	*AC091167.7*	*CD2BP2-DT*	*COL18A1-AS1*
842	*EIF4A1P9*	*AC015727.1*	*TUBB8P1*
843	*AL596223.1*	*AC018638.6*	*AL031719.2*

844	*AC005479.1*	*AC018647.3*	*WASHC5-AS1*
845	*AC004908.1*	*AP001178.1*	*DNAJA1P3*
846	*L1TD1*	*AC020558.1*	*LINC02610*
847	*AC073136.1*	*LINC00491*	*DLEU2*
848	*AC008786.1*	*AC020910.7*	*AL353152.2*
849	*AL606804.1*	*AC040173.1*	*RPL9P7*
850	*SPDYE18*	*AL162391.1*	*HOXB-AS3*
851	*CATSPERD*	*ATP1B1P1*	*AC008750.6*
852	*POLR3KP2*	*PALMD*	*AP002008.1*
853	*AC021231.2*	*AL109618.1*	*AC048380.2*
854	*AC007620.3*	*CPLX3*	*AC004692.1*
855	*BUD13P1*	*AC139834.1*	*XAF1*
856	*AC010776.3*	*AC026150.3*	*AC007952.7*
857	*AC008443.7*	*RPL23AP77*	*AC016597.1*
858	*KCNG2*	*AL513175.1*	*AL513320.1*
859	*CICP3*	*AL024508.1*	*FGD5*
860	*ADAMTS20*	*AC068282.1*	*NPY4R2*
861	*GPR1*	*AC004832.4*	*NTRK1*
862	*VAV1*	*CELP*	*PNMT*
863	*DRD5P1*	*AL161449.2*	*SLC2A5*
864	*AC016727.3*	*AC140134.1*	*EVI2A*
865	*SOX18*	*IFNA20P*	*FBN2*
866	*PLEK*	*AC004083.1*	*MIR99AHG*
867	*AL137058.2*	*LINC01559*	*MYCBP2-AS1*
868	*AL355607.2*	*CRHR2*	*AC027796.1*
869	*EIF4EBP3*	*SRP72P2*	*AC022784.6*
870	*AL691442.2*	*AC103746.1*	*LINC00618*
871	*STEAP4*	*FOXD4L3*	*AL391095.1*
872	*EEF1AKMT2*	*RHEX*	*AL162311.1*
873	*ATP6V0D2*	*AD001527.2*	*IPO4*
874	*ITK*	*AC021037.1*	*AC010996.1*
875	*HCN4*	*AC010300.1*	*AL121672.1*
876	*ZNF239*	*AC024610.2*	*PTENP1-AS*
877	*LRRC70*	*ZC3H12B*	*TBCEL-TECTA*

878 *IGFBP2*	*Z85996.2*	*AC016397.2*
879 *LINC00365*	*DLEU7*	*CORO1A*
880 *CNTNAP2*	*AC110772.2*	*FAM229B*
881 *DUSP23*	*AP003170.4*	*AL355802.1*
882 *ZNF311*	*SMAD1-AS2*	*AC005632.3*
883 *CPEB1*	*AC087564.1*	*ATOH8*
884 *LYPD6B*	*TAS2R63P*	*TIMM23B*
885 *CRNDE*	*AL117350.1*	*MTND5P11*
886 *S100A8*	*AC136469.2*	*STARD13-AS*
887 *FAHD2P1*	*OR7E104P*	*GCNT7*
888 *MRPS9-AS1*	*SIGLEC10-AS1*	*CSF3*
889 *LINC02561*	*AL096678.1*	*ZNF602P*
890 *TRIM61*	*LINC00867*	*Z97633.1*
891 *AC005332.1*	*OR11M1P*	*ITGB1BP2*
892 *AC064836.3*	*IPCEF1*	*AC007216.3*
893 *DMBT1*	*AC011498.4*	*AC131235.3*
894 *GREM1*	*CNGA4*	*BRCC3P1*
895 *SAA2-SAA4*	*AC080188.2*	*C1orf147*
896 *AC104066.5*	*AC006296.3*	*H2BC17*
897 *OCA2*	*HMGN1P15*	*MBL1P*
898 *AC009065.2*	*AP005432.1*	*AL161891.1*
899 *GABRD*	*AC093928.1*	*MIR17HG*
900 *MAPK4*	*SPATA45*	*AC114550.1*
901 *AC021851.1*	*SALL4P7*	*AC019068.1*
902 *CR2*	*KCNE1*	*DERPC*
903 *TSPAN19*	*FAR1-IT1*	*FOSL1*
904 *RIMS2*	*AC103739.2*	*SLC25A10*
905 *GLI1*	*POU5F1P6*	*AC005829.1*
906 *TSPAN7*	*BCAR3-AS1*	*HOXA7*
907 *LCTL*	*ZNF69*	*AL513128.3*
908 *AC068446.2*	*EEF1A1P3*	*OR7E126P*
909 *AC138956.1*	*MUC4*	*CCBE1*
910 *LINC02392*	*SLC36A2*	*AC130324.3*
911 *LINC01342*	*AC013470.2*	*CEP164P1*

912	*AP003072.5*	*AL162171.2*	*PFKFB3*
913	*AL049833.3*	*AL645768.1*	*COQ3*
914	*AJ011931.1*	*WDR64*	*LINC01116*
915	*RPL37P12*	*AL590235.2*	*AC025062.2*
916	*DLX2-DT*	*AC110056.1*	*Z84478.1*
917	***LINC02617***	*AC108865.2*	*AL450998.2*
918	*EID3*	*LMO7DN*	*GALNT16*
919	*PIK3C2G*	*AC127024.6*	*TCAF2P1*
920	*NPR3*	*DBIL5P2*	*BLNK*
921	*AL353743.4*	*CPHXL*	*AP001178.2*
922	*SEC23A-AS1*	*GTF2IRD1P1*	*AC135279.2*
923	*ALOX5*	*NPM1P21*	*BCL2A1*
924	*AC009879.3*	*ASNSP3*	*VPS9D1-AS1*
925	*DRD5P2*	*RPL7AP30*	*KIAA0408*
926	*FAM189A2*	*OR6S1*	*LINC01097*
927	*NELL2*	*AC004865.1*	*AP001001.1*
928	*IQGAP2*	*AC103691.2*	*AC106873.8*
929	*CPNE9*	*AC011840.1*	*LINC00311*
930	*AL392046.1*	*PTX4*	*AC016598.2*
931	*NUS1P1*	*ALB*	*AC105429.1*
932	*GXYLT1*	*AC097634.1*	*AC148477.3*
933	*ZFPM2-AS1*	*AC016738.1*	*ZBP1*
934	*SLC4A10*	*AC015911.11*	*AC074194.2*
935	*RCOR2*	*CD5*	*AC253576.2*
936	*RAB42*	*AC093675.2*	*ANKRD1*
937	*ZNF619*	*CCL24*	*EPO*
938	*AC010463.1*	*TACR1*	*GADL1*
939	*DGUOK-AS1*	*LARP4P*	*AC064807.4*
940	*RBM24*	*CDRT15L2*	*AC007786.1*
941	*CYTL1*	*SALRNA3*	*ARHGAP42-AS1*
942	*AC245041.2*	*AL139300.2*	*AL138762.1*
943	*HECW2*	*AC024267.5*	*PRR20G*
944	*TMEM255A*	*AC092162.2*	*AC002401.3*
945	*AC009065.5*	***GRIK1***	*FCRL2*

946	*XK*	*ZFP28*	*AC099845.1*
947	*AC074141.1*	*BX088651.4*	*GSG1*
948	*AL117327.1*	*AC009244.2*	*HNRNPA1P27*
949	*AP001020.2*	*FAAH2*	*TGFB2-AS1*
950	*LAG3*	*ABCG8*	*POTEKP*
951	*IL1RN*	*AL592293.2*	*BNIP3P17*
952	*LINC01943*	*AC096719.1*	*AC115618.1*
953	*TNFAIP6*	*AC020934.1*	*EEF1AKMT2*
954	*PLAAT1*	*IL18RAP*	*AC100803.2*
955	*ZNF365*	*MICU3*	*HADHAP1*
956	*AL355916.1*	*BZW1P1*	*UQCC3*
957	*MCPH1-AS1*	*AC010327.3*	*TSSK6*
958	*LINC01828*	*AC004832.6*	*AL138963.3*
959	*GFI1*	*DINOL*	*AC092135.3*
960	*PCED1B-AS1*	*AC004584.3*	*AL022324.2*
961	*TTC26*	*ST8SIA5*	*FLRT2*
962	*RBP7*	*CCND2*	*AC137630.5*
963	*AC068446.1*	*CICP24*	*ZNF469*
964	*LINC00842*	*INA*	*TRPM3*
965	*AC020612.3*	*AP001330.4*	*TUBAP2*
966	*SDS*	*AL390728.3*	*AC099513.1*
967	*DRC1*	*TG*	*AC013643.2*
968	*CSRP1*	*HAS1*	*AC018804.1*
969	*PCDH17*	*CRIP3*	*KCNV2*
970	*RBPMS2*	*FAM230H*	*AC011700.1*
971	*SIAH2-AS1*	*GAPDHP16*	*AC026120.3*
972	*LINC02202*	*GGT5*	*SNX18P23*
973	*DMC1*	*CNGB3*	*GJC3*
974	*CYP2F1*	*AC105411.1*	*SUGT1P4-STRA6LP*
975	*PTPRR*	*CYP2F1*	*HMGA2-AS1*
976	*LINC00900*	*SMLR1*	*CNN2P9*
977	*GBP6*	*AL929472.1*	*PABPC4-AS1*
978	*AP001266.1*	*AL442003.1*	*Z73965.1*
979	*LINC01564*	*AC010883.1*	*AC130651.1*

980	*PLCB2*	*SPP1*	*CFAP46*
981	*TWIST2*	*FAM21EP*	*TDGF1P5*
982	*SYT8*	*TH*	*HSP90B2P*
983	*DIO1*	*YIPF7*	*LRRC4*
984	*LINC00870*	*LINC02548*	*FLJ13224*
985	*AL121820.1*	*AC122688.4*	*PSG5*
986	*AL139156.2*	*AC145207.3*	*AC131009.1*
987	*OLFML2B*	*AC022467.1*	*PDX1*
988	*LINC02073*	***FGD5***	*AL353997.2*
989	*AC087222.1*	*PLAAT1*	*AC039056.2*
990	*LRRC15*	*AC007682.1*	*POP1*
991	*ARGFXP2*	*AC073365.1*	*AL020995.1*
992	*AC124067.2*	*AP001615.1*	*HAGLROS*
993	*AL034405.1*	*AC011406.1*	*AC073592.3*
994	*SILC1*	*AC005521.1*	*AC090515.1*
995	*CFB*	*KCNK9*	*AL034405.1*
996	*FAM91A2P*	*TUBAP2*	*AL590326.1*
997	*AL604028.1*	*LINC02222*	*OPRM1*
998	*AC026979.2*	*AC068802.1*	*ZNF30-AS1*
999	*AC015802.5*	*AC107464.1*	*ZNF114-AS1*
1000	*AC144530.1*	*GREM1*	*AC091076.1*

Note: entries with names highlighted in red indicate genes co-upregulated in all three examined PCa lines.

Supplementary Table 2. A representative list of primer sequences for expression assay by qRT-PCR

Target name	Forward (5'-3')	Reverse (5'-3')
IL6	TACCCCCAGGAGAAGATTCC	TTTTCTGCCAGTGCCTCTTT
CXCL8	GTGCAGTTTTGCCAAGGAGT	CTCTGCACCCAGTTTTCCTT
IL1α	AATGACGCCCTCAATCAAAG	TGGGTATCTCAGGCATCTCC
IL1β	GGGCCTCAAGGAAAAGAATC	TTCTGCTTGAGAGGTGCTGA
IL7	GGACTTCCTCCCCTGATCCT	TCAGTGTTCTTTAGTGCCCATCA
CSF1	CCAGCAACTTCCTCTCAGCA	GAATCCGCTCTCTGAGGCTC
CSF2	CTGCTGCTCTTGGGCACT	CCAGCAGTCAAAGGGGATGA
CXCL1	AGGGAATTCACCCCAAGAAC	TGGATTTGTCACTGTTCAGCA
CXCL3	GCAGGGAATTCACCTCAAGA	GGTGCTCCCCTTGTTCAGTA
MMP1	GGTCTCTGAGGGTCAAGCAG	AGTTCATGAGCTGCAACACG
MMP3	GCAGTTTGCTCAGCCTATCC	GAGTGTCGGAGTCCAGCTTC
MMP10	CGTTGGTCACTTCAGCTCCT	CACTTGGCTGGCATCTCAGA
GM-CSF	CCCCAGTCACCTGCTGTTAT	TGGAATCCTGAACCCACTTC
SFRP2	GCCTCGATGACCTAGACGAG	GATGCAAAGGTCGTTGTCCT
SPINK1	CCTTGGCCCTGTTGAGTCTA	GCCCAGATTTTTGAATGAGG
EREG	CGTGTGGCTCAAGTGTCAAT	TGGAACCGACGACTGTGATA
ANGPTL4	GCCTATAGCCTGCAGCTCAC	AGTACTGGCCGTTGAGGTTG
AREG	TGGATTGGACCTCAATGACA	AGCCAGGTATTTGTGGTTCG
WNT16B	GCTCCTGTGCTGTGAAAACA	TGCATTCTCTGCCTTGTGTC
TIMP1	CATCCTGTTGTTGCTGTGGC	GGAAGCCCTTTTCAGAGCCT
CXCL12	ATGAACGCCAAGGTCGTGG	TCGGGTCAATGCACACTTGT
CCK	GTGCTGATGGCGGTACTGG	CTCATACTCCTCGGCACTGC
p16	GGGTCGGGTAGAGGAGGTG	AATCGGGGATGTCTGAGGGA
p21	CTGGGGATGTCCGTCAGAAC	TCGACCCTGAGAGTCTCCAG
MCT1	ATGATCGCTGGTGGTTGTCT	CAAGTTGAAAGCAAGCCCAA
MCT4	TGGGATGGGACTGACTTTTC	CCATGTGCAGACAAACTGCT
GLUT1	CAGGCTGTGCTGTGCTCAT	TTCAAAGAAGGCCACAAGC
GLUT2	GGAGTTGGCGCTGTAAACAT	AAACTCAGCCACCATGAACC
GLUT3	ACCGGCTTCCTCATTACCTT	AGGCTCGATGCTGTTCATCT
PFK1	CCCGTGTCTTCTTTGTCCAT	GTTGTAGGCAGCTCGGAGTC
PKM2	CTATCCTCTGGAGGCTGTGC	CCAGACTTGGTGAGGACGAT
PGK1	CTGTGGGGGTATTTGAATGG	CTTCCAGGAGCTCCAAACTG
PGI	CGCCCAACCAACTCTATTGT	GGTAGAAGCGTCGTGAGAGG
IDH1	GCTTCATCTGGGCCTGTAAA	GCTTTGCTCTGTGGGCTAAC
IDH2	TGGCTCAGGTCCTCAAGTCT	CTCAGCCTCAATCGTCTTCC
IDH3A	AACATCATGCGGATGTCAGA	CAATGTTGCCACTTGGTGTC
IDH3B	AAGTGGCCATCATTGGAAAG	ATCACACCCCTTGCACTCTC
OGDH	TGAGGAAGCTTTTGCCAGAT	AACTAGCCACATTCCCGATG

CS	CCATCCACAGTGACCATGAG	CTTTGCCAACTTCCTTCTGC
LDHA	ACGTCAGCAAGAGGGAGAAA	CGCTTCCAATAACACGGTTT
PDK4	CCTTTGGCTGGTTTTGGTTA	CCTGCTTGGGATACACCAGT
HTR2B	GCCTTCTTCACACCTCTTGC	TGTCCTTTCGAGAACCATCC
MCT1	TCCAGCTCTGACCATGATTG	GCCCCCAAGAATTAGAAAGC
MCT4	TGGGATGGGACTGACTTTTC	CCATGTGCAGACAAACTGCT
NOX1	TTAACAGCACGCTGATCCTG	CTGGAGAGAATGGAGGCAAG
NOX2	TCACTTCCTCCACCAAAACC	GGGATTGGGCATTCCTTTAT
NOX3	CCAGGGCAGTACATCTTGGT	ACATCTGTCAGGGCAGTTCC
NOX4	CTTCCGTTGGTTTGCAGATT	TGGGTCCACAACAGAAAACA
NOX5	CTACGTGGTAGTGGGGCTGT	ATGCAGGAACTGGAGCAGAT
DUOX1	TGGCTATCACGTGCTTTCAG	GGAATGCGAGGAACCATAGA
DUOX2	GGCTCCCCAGAGGATAAGTC	GTCAGCTCCTCCTTGTCCTG
RPL13A	GTACGCTGTGAAGGCATCAA	CGCTTTTTCTTGTCGTAGGG

Supplementary Table 3. A list of DNA oligos for generation of constructs encoding gene-specific shRNAs

Target name		Primers	Sequences (5'-3')
PDK4	shRNA #1	F	CCGGGTGCAAGACTACAGGAGTTAACTCGAG TTAACTCCTGTAGTCTTGCACTTTTTG
		R	AATTCAAAAAGTGCAAGACTACAGGAGTTAA CTCGAG TTAACTCCTGTAGTCTTGCAC
	shRNA #2	F	CCGGGTTCGAAATAGACACCATAATCTCGAG ATTATGGTGTCTATTTCGAAC TTTTTG
		R	AATTCAAAAAGTTCGAAATAGACACCATAATC TCGAG ATTATGGTGTCTATTTCGAAC
	shRNA #3	F	CCGGCCCGTGTCAATTAACATTTAACTCGAG TTAAATGTTAATTGACACGGGTTTTTG
		R	AATTCAAAAACCCGTGTCAATTAACATTTAA CTCGAG TTAAATGTTAATTGACACGGG
Scramble		F	CCGGCAACAAGATGAAGAGCACCAACTCGAG TTGGTGCTCTTCATCTTGTTGTTTTTG
		R	AATTCAAAAACAACAAGATGAAGAGCACCAAC TCGAGTTGGTGCTCTTCATCTTGTTG

Supplementary Table 4. A list of antibodies applied for immunoblot and immunofluorescence staining assays

Target name	Antibody source	Vendor and Cat. No.	Dilution
PDK1	mouse	Santa Cruz, sc-515944	1:1000, immunoblot
PDK2	mouse	Santa Cruz, sc-100534	1:1000, immunoblot
PDK3	mouse	Santa Cruz, sc-365378	1:1000, immunoblot
PDK4	mouse	Santa Cruz, sc-518061	1:1000, immunoblot; 1:400, IHC staining
LDHA	mouse	Santa Cruz, sc-137243	1:1000, immunoblot
MMP3	mouse	Proteintech, 66338-1-Ig	1:500, immunoblot
IL6	mouse	Proteintech, 66146-1-Ig	1:500, immunoblot
CXCL8	mouse	abcam, ab18672	1:1000, immunoblot; 1:250, IHC staining
Caspase 3 (cleaved)	rabbit	Cell Signaling, 9661	1:1000, immunoblot; 1:250, IHC staining
MCT1	rabbit	Cell Signaling, 36768	1:1000, immunoblot
MCT4	rabbit	abcam, ab244385	1:1000, immunoblot
NOX1	rabbit	abcam, ab131088	1:1000, immunoblot
NOX2	rabbit	abcam, ab129068	1:1000, immunoblot
NOX3	rabbit	abcam, ab81864	1:1000, immunoblot
NOX4	rabbit	abcam, ab154244	1:500, immunoblot
γH2AX	mouse	EMD Millipore, JBW301, 05-636-25UG	1:500, immunoblot; 1:250, IHC staining
H2AX	rabbit	Cell Signaling, 7631	1:1000, immunoblot
$p16^{INK4a}$	mouse	BD Pharmingen, G175-1239, 554079	1:500, immunoblot
β-actin	mouse	Proteintech, 66009-1-Ig	1:4000, immunoblot
GAPDH	rabbit	Abways, AB0037	1:2000, immunoblot

Source Data (uncropped scans of blots and gels)

Supplementary Fig. 5a

PDK4

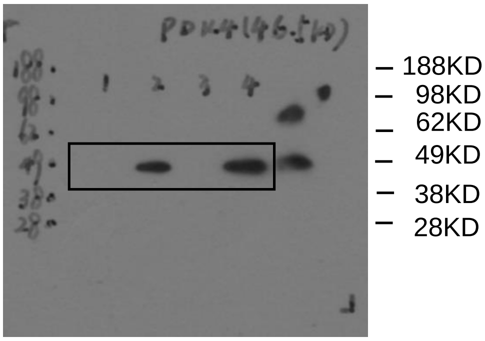

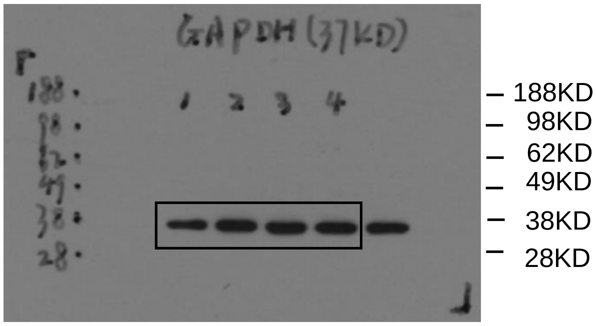

GAPDH
