# Supplementary material for: PDK4-dependent hypercatabolism and lactate production of senescent cells promotes cancer malignancy
Source: Nat Metab. 2023 Oct 30;5(11):1887–910. doi: 10.1038/s42255-023-00912-w (PMC10663165; doi:10.1038/s42255-023-00912-w)
Supplement: Supplementary file 2 — Reporting Summary [file 42255_2023_912_MOESM2_ESM.pdf]

nature portfolio

Corresponding author(s): Yu Sun, Yuzheng Zhao

Last updated by author(s): Sep 19, 2023

Reporting Summary

Nature Portfolio wishes to improve the reproducibility of the work that we publish. This form provides structure for consistency and transparency in reporting. For further information on Nature Portfolio policies, see our Editorial Policies and the Editorial Policy Checklist.

Statistics

For all statistical analyses, confirm that the following items are present in the figure legend, table legend, main text, or Methods section.

n/a	Confirmed	
☐	☒	The exact sample size (*n*) for each experimental group/condition, given as a discrete number and unit of measurement
☐	☒	A statement on whether measurements were taken from distinct samples or whether the same sample was measured repeatedly
☐	☒	The statistical test(s) used AND whether they are one- or two-sided<br>*Only common tests should be described solely by name; describe more complex techniques in the Methods section.*
☒	☐	A description of all covariates tested
☐	☒	A description of any assumptions or corrections, such as tests of normality and adjustment for multiple comparisons
☐	☒	A full description of the statistical parameters including central tendency (e.g. means) or other basic estimates (e.g. regression coefficient) AND variation (e.g. standard deviation) or associated estimates of uncertainty (e.g. confidence intervals)
☐	☒	For null hypothesis testing, the test statistic (e.g. *F*, *t*, *r*) with confidence intervals, effect sizes, degrees of freedom and *P* value noted<br>*Give P values as exact values whenever suitable.*
☒	☐	For Bayesian analysis, information on the choice of priors and Markov chain Monte Carlo settings
☒	☐	For hierarchical and complex designs, identification of the appropriate level for tests and full reporting of outcomes
☐	☒	Estimates of effect sizes (e.g. Cohen's *d*, Pearson's *r*), indicating how they were calculated

Our web collection on statistics for biologists contains articles on many of the points above.

Software and code

Policy information about availability of computer code

Data collection	IN Cell Analyzer 2500HS GE Healthcare<br>QuantStudio Real-Time PCR Software v1.1 Applied Biosystems<br>XF24 Extracellular Flux Analyzer
Data analysis	GraphPad Prism 9 (v 9.5.1)<br>FlowJo (v 10.8.1)<br>Image J (v 1.52p)<br>FASTQC (v 0.11.5)<br>Ensembl (v 72) gene annotation<br>FUNRICH (v 3)<br>Ingenuity Pathway Analysis (v 2020.12)<br>PANTHER (v 16.0)<br><br>R packages (v 4.0.3) from CRAN and Bioconductor (v 3.17):<br>custom R (v 4.0) scripts<br>annotate (v 1.56.2)<br>ensembldb (v 2.2.2)<br>edgeR (v 3.22.3)<br>fdrtool (v 1.2.16)<br>Bedtools2 (v 2.26.0)<br>Bowtie (v 2.2.2)

DESeq2 (v 1.18.1)
ggplot2 (v 3.1.0)
ggrepel (v 0.8.0)
gplots (v 3.0.1)
graph (v 1.56.0)
gridExtra (v 2.3)

pd.hta.2.0 (v 3.12.2)
pheatmap (v 1.0.10)
plotly (v 4.8.0)
plotrix (v 3.7.4)
plyr (v 1.8.4)
preseqR (v 3.1.2)

For manuscripts utilizing custom algorithms or software that are central to the research but not yet described in published literature, software must be made available to editors and reviewers. We strongly encourage code deposition in a community repository (e.g. GitHub). See the Nature Portfolio guidelines for submitting code & software for further information.

Data

Policy information about availability of data

All manuscripts must include a data availability statement. This statement should provide the following information, where applicable:

- Accession codes, unique identifiers, or web links for publicly available datasets
- A description of any restrictions on data availability
- For clinical datasets or third party data, please ensure that the statement adheres to our policy

Source data for all main figures and Extended Data figures are supplied with this paper. Experimental data supporting the plots within this paper and other findings of this study are available from the corresponding author upon reasonable request. The RNA-seq data generated in the present study have been deposited in the Gene Expression Omnibus database under accession codes GSE198110, GSE217808 and GSE222279, respectively.

Human research participants

Policy information about studies involving human research participants and Sex and Gender in Research.

Reporting on sex and gender	Clinical findings regarding prostate cancer (PCa) apply only to male patients (Extended Data Fig. 2), while those regarding breast cancer (BCa) apply only to female patients (Supplementary Fig. 1). Sex or gender was neither considered in study design nor determined on self-reporting or assigned.
Population characteristics	Administration of chemotherapeutic agents was performed for primary PCa patients (Clinical trial no. NCT03258320) and infiltrating ductal BCa patients (NCT02897700), by following the CONSORT 2010 Statement (updated guidelines for reporting parallel group randomized trials). Patients with a clinical stage ≥ I subtype A (IA) (T1a, N0, M0) of primary cancer but without manifest distant metastasis were enrolled into the multicentred, randomized, double-blinded and controlled pilot studies. Age between 40-75 years with histologically proven PCa, or age ≥ 18 years with histologically proven infiltrating ductal BCa was required for recruitment into the clinical cohorts. Data regarding tumour size, histologic type, tumour penetration, lymph node metastasis, and TNM stage were obtained from the pathologic records.
Recruitment	Patients with a clinical stage ≥ I subtype A (IA) (T1a, N0, M0) of primary cancer but without manifest distant metastasis were enrolled into the multicentred, randomized, double-blinded and controlled pilot studies. Age between 40-75 years with histologically proven PCa, or age ≥ 18 years with histologically proven infiltrating ductal BCa was required for recruitment into the clinical cohorts. The participants were recruited according to their clinical diagnosis profiles, and there was no self-selection bias or other biases applied in patient recruitment throughout this study.
Ethics oversight	Randomized control trial (RCT) protocols and all experimental procedures were approved by the Institutional Review Board of Shanghai Jiao Tong University School of Medicine, with methods carried out in accordance with the official guidelines.

Note that full information on the approval of the study protocol must also be provided in the manuscript.

Field-specific reporting

Please select the one below that is the best fit for your research. If you are not sure, read the appropriate sections before making your selection.

☒ Life sciences ☐ Behavioural & social sciences ☐ Ecological, evolutionary & environmental sciences

For a reference copy of the document with all sections, see nature.com/documents/nr-reporting-summary-flat.pdf

Life sciences study design

All studies must disclose on these points even when the disclosure is negative.

Sample size	Sample size was not determined by specific statistical methods a priori, but were based on variability of associated assays. The sample sizes are similar to those reported in previous publications with comparable experiments (Chang et al., Nature Medicine. 2016; Zhang et al., Nature Communications. 2018; Xu et al., Nature Medicine. 2018; Guerrero et al., Nature Metabolism. 2019). We did not focus on a specific effect-size and performed a discovery study, including all available samples that passed QC into analysis. To our knowledge, a comparative senescence-associated metabolic phenotype-targeting investigation with a PDK4-specific chemical or agent has not been previously undertaken.
Data exclusions	All data were included, without specific exclusions.
Replication	All experiments were reproducible. Every figure states how many times the related experiment was performed with similar results. All data presented were from independent biological replicates or independent experiments. All attempts at replication were successful.
Randomization	For high-throughput data acquisition and database generation, samples were randomized between batches to account for possible batch-effect. Stringent inclusion criteria were set to account for other possible confounding variables. For preclinical experiments, animals were randomly assigned to each individual groups.
Blinding	The investigators were not blinded to sample group allocations due to the fact that the phenotypes of human primary cells, needed to be carefully documented by the investigators, so blinding was not always possible during experimental setup, such as in the case of characterization of senescence-associated phenotypic alterations (SA-B-Gal staining, BrdU staining and live-cell fluorescence imaging). When feasible, data analysis was performed blind, including RNA and protein preparation, q-PCR and immunoblots, routine immunofluorescence staining, RNA-seq library preparation, bioinformatics profiling, evaluation of histological sections from preclinical biospecimens, for which all data acquisition was performed blinded to types of individual samples.

Reporting for specific materials, systems and methods

We require information from authors about some types of materials, experimental systems and methods used in many studies. Here, indicate whether each material, system or method listed is relevant to your study. If you are not sure if a list item applies to your research, read the appropriate section before selecting a response.

Materials & experimental systems

n/a	Involved in the study
☐	☒ Antibodies
☐	☒ Eukaryotic cell lines
☒	☐ Palaeontology and archaeology
☐	☒ Animals and other organisms
☐	☒ Clinical data
☒	☐ Dual use research of concern

Methods

n/a	Involved in the study
☒	☐ ChIP-seq
☒	☐ Flow cytometry
☒	☐ MRI-based neuroimaging

Antibodies

Antibodies used	The following antibodies were purchased from the indicated suppliers and used for immunoblotting (if not stated; or otherwise, immunofluorescence staining or immunohistochemistry staining, as stated separately) at indicated concentrations: mouse monoclonal anti-PDK1 (Santa Cruz cat. no. sc-515944), 1:1000; mouse monoclonal anti-PDK2 (Santa Cruz cat. no. sc-100534), 1:1000; mouse monoclonal anti-PDK3 (Santa Cruz cat. no. sc-365378), 1:1000; mouse monoclonal anti-PDK4 (Santa Cruz cat. no. sc518061), 1:1000 (1:400 for immunohistochemistry staining); mouse monoclonal anti-LDHA (Santa Cruz cat. no. sc-137243), 1:1000; rabbit polyclonal anti-HTR2B (Abways cat. no. AY1011), 1:500; mouse monoclonal anti-MMP3 (Proteintech, cat. no. 66338-1-Ig), 1:500; mouse monoclonal anti-IL6 (Proteintech, cat. no. 66146-1-Ig), 1:500; mouse monoclonal anti-CXCL8 (Abcam cat. no. ab18672), 1:1000; rabbit monoclonal anti-Caspase 3 (cleaved) (Cell Signaling cat. no. 9661, clone Asp175), 1:1000 (or 1:250 for immunohistochemistry staining); rabbit monoclonal anti-MCT1 (Cell Signaling cat. no. 36768), 1:1000; rabbit polyclonal anti-MCT4 (abcam cat. no. ab244385), 1:1000; rabbit polyclonal anti-NOX1 (abcam cat. no. ab131088), 1:1000; rabbit monoclonal anti-NOX2 (abcam cat. no. ab129068), 1:1000; rabbit polyclonal anti-NOX3 (abcam cat. no. ab81864), 1:1000; rabbit polyclonal anti-NOX4 (abcam cat. no. ab154244), 1:500; mouse monoclonal anti-γH2AX (EMD Millipore JBW301 cat. no. 05-636-25UG), 1:500 (or 1:250 for immunofluorescence staining); rabbit monoclonal anti-H2AX (Cell Signaling Technology cat. no. 7631), 1:1000; mouse monoclonal anti-p16 (BD Pharmingen G175-1239 cat. no. 554079), 1:500; mouse monoclonal anti-β-actin (Proteintech cat. no. 66009-1-Ig), 1:4000; rabbit monoclonal anti-GAPDH (Abways cat. no. AB0037), 1:2000; goat polyclonal anti-rabbit IgG H&L (HRP) (abcam cat. no. ab6721), 1:500; goat polyclonal anti-mouse IgG H&L (HRP) (abcam cat. no. ab6789), 1:500; goat polyclonal to rabbit (or mouse) IgG Alexa Fluor 488 or 594-conjugated secondary (abcam cat. no. ab150077, ab150080, ab150113, ab150116), 1:400 (for immunofluorescence staining);
Validation	Antibody validations were performed by antibody suppliers per quality assurance literature provided by each supplier for applications used in this study (see links below).<br>mouse monoclonal anti-PDK1 (Santa Cruz cat. no. sc-515944), immunoblotting

https://www.scbt.com/p/pdk1-antibody-e-10?requestFrom=search
mouse monoclonal anti-PDK2 (Santa Cruz cat. no. sc-100534), immunoblotting
https://www.scbt.com/zh/p/pdk2-antibody-s-15
mouse monoclonal anti-PDK3 (Santa Cruz cat. no. sc-365378), immunoblotting
https://www.scbt.com/zh/p/pdk3-antibody-a-4
mouse monoclonal anti-PDK4 (Santa Cruz cat. no. sc518061), immunoblotting and immunohistochemistry staining
https://www.scbt.com/zh/p/pdk4-antibody-b-1
mouse monoclonal anti-LDHA (Santa Cruz cat. no. sc-137243), immunoblotting
https://www.scbt.com/p/ldh-a-antibody-e-9?requestFrom=search
rabbit polyclonal anti-HTR2B (Abways cat. no. AY1011), immunoblotting
http://www.abways.com/showproduct.asp?cid=AY1011
mouse monoclonal anti-MMP3 (Proteintech cat. no. 66338-1-Ig), immunoblotting
https://www.ptglab.com/products/MMP3-Antibody-66338-1-Ig.htm
mouse monoclonal anti-IL6 (Proteintech cat. no. 66146-1-Ig), immunoblotting
https://www.ptgcn.com/products/IL6-Antibody-66146-1-Ig.htm
mouse monoclonal anti-CXCL8 (Abcam cat. no. ab18672), immunoblotting
https://www.abcam.cn/il-8-antibody-807-ab18672.html
rabbit monoclonal cleaved-Caspase 3 (Cell Signaling cat. no. 9661), immunoblotting and immunohistochemistry staining
https://www.cellsignal.cn/products/primary-antibodies/cleaved-caspase-3-asp175-antibody/9661
rabbit monoclonal anti-MCT1 (Cell Signaling cat. no. 36768), immunoblotting
https://www.cellsignal.com/products/primary-antibodies/mct1-slc16a1-e7f6y-rabbit-mab/36768?_=1673847527880&Ntt=MCT1&tahead=true
rabbit polyclonal anti-MCT4 (abcam cat. no. ab244385), immunoblotting
https://www.abcam.com/slc16a3mct-4-antibody-ab244385.html
rabbit polyclonal anti-NOX1 (abcam cat. no. ab131088), immunoblotting
https://www.abcam.com/nox1-antibody-ab131088.html
rabbit monoclonal anti-NOX2 (abcam cat. no. ab129068), immunoblotting
https://www.abcam.com/nox2gp91phox-antibody-epr6991-ab129068.html
rabbit polyclonal anti-NOX3 (abcam cat. no. ab81864), immunoblotting
https://www.abcam.com/products/primary-antibodies/nox3-antibody-ab81864.html
rabbit polyclonal anti-NOX4 (abcam cat. no. ab154244), immunoblotting
https://www.abcam.com/nadph-oxidase-4-antibody-ab154244.html
mouse monoclonal anti-γH2AX (EMD Millipore JBW301 cat. no. 05-636-25UG), immunoblotting and immunofluorescence staining
https://www.merckmillipore.com/CN/zh/product/Anti-phospho-Histone-H2A.X-Ser139-Antibody-clone-JBW301,MM_NF-05-636-25UG
rabbit monoclonal anti-H2AX (Cell Signaling Technology cat. no. 7631), immunoblotting
https://www.cellsignal.com/products/primary-antibodies/histone-h2a-x-d17a3-xp-rabbit-mab/7631
mouse monoclonal anti-p16 (BD Pharmingen G175-1239 cat. no. 554079), immunoblotting
https://www.bdbiosciences.com/zh-cn/search-results?searchKey=554079
mouse monoclonal anti-β-actin (Proteintech cat. no. 66009-1-Ig), immunoblotting
https://www.ptgcn.com/products/Pan-Actin-Antibody-66009-1-Ig.htm
rabbit monoclonal anti-GAPDH (Abways cat. no. AB0037), immunoblotting
http://www.abways.com/showproduct.asp?cid=AB0037
goat polyclonal anti-rabbit IgG H&L (HRP) (abcam cat. no. ab6721), immunoblotting
https://www.abcam.com/goat-rabbit-igg-hl-hrp-ab6721.html
goat polyclonal anti-mouse IgG H&L (HRP) (abcam cat. no. ab6789), immunoblotting
https://www.abcam.com/goat-mouse-igg-hl-hrp-ab6789.html
goat polyclonal to rabbit (or mouse) IgG Alexa Fluor 488 or 594-conjugated secondary (abcam cat. no. ab150077, ab150080, ab150113, ab150116), immunofluorescence staining
https://www.abcam.com/goat-rabbit-igg-hl-alexa-fluor-488-ab150077.html
https://www.abcam.com/goat-rabbit-igg-hl-alexa-fluor-594-ab150080.html
https://www.abcam.com/goat-mouse-igg-hl-alexa-fluor-488-ab150113.html
https://www.abcam.com/goat-mouse-igg-hl-alexa-fluor-594-ab150116.html

Anti-PDK1 mouse monoclonal, Santa Cruz, cat. no. sc-515944. Validated by the company and the following publication: Yoo, H. C., et al. A Variant of SLC1A5 Is a Mitochondrial Glutamine Transporter for Metabolic Reprogramming in Cancer Cells. Cell Metab. 2020. 31(2): 267-283 e12. DOI: 10.1016/j.cmet.2019.11.020.

Anti-PDK2 mouse monoclonal, Santa Cruz, cat. no. sc-100534. Validated by the company and the following publication: Fukushima, A., et al. Acetylation contributes to hypertrophy-caused maturational delay of cardiac energy metabolism. JCI Insight. 2018. 3(10): e99239. DOI: 10.1172/jci.insight.99239.

Anti-PDK3 mouse monoclonal, Santa Cruz, cat. no. sc-365378. Validated by the company and the following publication: Zhao, J., et al. Deamidation Shunts RelA from Mediating Inflammation to Aerobic Glycolysis. Cell Metab. 2020. 31(5): 937-955. e7. DOI: 10.1016/j.cmet.2020.04.006.

Anti-PDK4 mouse monoclonal, Santa Cruz, cat. no. sc518061. Validated by the company and the following publication: Odeh, M., et al. P38alpha MAPK coordinates the activities of several metabolic pathways that together induce atrophy of denervated muscles. FEBS J. 2020. 287(1): 73-93. DOI: 10.1111/febs.15070.

Anti-LDHA mouse monoclonal, Santa Cruz, cat. no. sc-137243. Validated by the company and the following publication: 1. Yang, W., et al. ERK1/2-dependent phosphorylation and nuclear translocation of PKM2 promotes the Warburg effect. Nat Cell Biol. 2012. 14(12): 1295-1304. DOI: 10.1038/ncb2629. 2. Huang, X., et al. The HGF-MET axis coordinates liver cancer metabolism and autophagy for chemotherapeutic resistance. Autophagy. 2019. 15(7): 1258-1279. DOI: 10.1080/15548627.2019.1580105. 3. Sikorski, K., et al. A high-throughput pipeline for validation of antibodies. Nat Methods. 2018. 15(11): 909-912. DOI: 10.1038/s41592-018-0179-8.

Anti-HTR2B rabbit polyclonal, Abways, cat. no. AY1011. Validated by the company and the following publication: Choi, W. G., et al. Inhibiting serotonin signaling through HTR2B in visceral adipose tissue improves obesity-related insulin resistance. J Clin Invest. 2021. 131(23): e145331. DOI: 10.1172/JCI145331.

Anti-MMP3 mouse monoclonal, Proteintech, cat. no. 66338-1-Ig. Validated by the company and the following publication: Lehner, C., et al., Allergy-induced systemic inflammation impairs tendon quality. EBioMedicine. 2022. 75:103778. DOI: 10.1016/j.ebiom.2021.103778.

Anti-IL6 mouse monoclonal, Proteintech cat. no. 66146-1-Ig. Validated by the company and the following publication: Ma, X., et al. A stress-induced cilium-to-PML-NB route drives senescence initiation. Nat Commun. 2023. 14(1):1840. DOI: 10.1038/s41467-023-37362-7.

Anti-CXCL8 mouse monoclonal, Abcam, cat. no. ab18672. Validated by the company and the following publication: Xu, Q., et al. The flavonoid procyanidin C1 has senotherapeutic activity and increases lifespan in mice. Nat Metab. 2021. 3(12):1706-1726. DOI: 10.1038/s42255-021-00491-8.

Anti-cleaved-Caspase 3 rabbit monoclonal, Cell Signaling, cat. no. 9661. Validated by the company and the following publication: Xu, Q., et al. The flavonoid procyanidin C1 has senotherapeutic activity and increases lifespan in mice. Nat Metab. 2021. 3(12): 1706-1726. DOI: 10.1038/s42255-021-00491-8.

Anti-MCT1 rabbit monoclonal, Cell Signaling, cat. no. 36768. Validated by the company and the following publication: Khan, A., et al. Targeting metabolic activity in high-risk neuroblastoma through Monocarboxylate Transporter 1 (MCT1) inhibition. Oncogene. 2020. 39(17): 3555-3570. DOI: 10.1038/s41388-020-1235-2.

Anti-MCT4 rabbit polyclonal, abcam, cat. no. ab244385. Validated by the company and the following publication: Wakamatsu, K., et al. Metabolites and Biomarker Compounds of Neurodegenerative Diseases in Cerebrospinal Fluid. Metabolites. 2022. 12(4): 343. DOI: 10.3390/metabo12040343.

Anti-NOX1 rabbit polyclonal, abcam, cat. no. ab131088. Validated by the company and the following publication: Ko, J. et al. Protective Effect of GIP against Monosodium Glutamate-Induced Ferroptosis in Mouse Hippocampal HT-22 Cells through the MAPK Signaling Pathway. Antioxidants (Basel). 2022. 11(2): 189. DOI: 10.3390/antiox11020189.

Anti-NOX2 rabbit monoclonal, abcam, cat. no. ab129068. Validated by the company and the following publication: Yin, Y. L. et al. Citronellal Attenuates Oxidative Stress-Induced Mitochondrial Damage through TRPM2/NHE1 Pathway and Effectively Inhibits Endothelial Dysfunction in Type 2 Diabetes Mellitus. 2022. Antioxidants (Basel). 11(11): 2214. DOI: 10.3390/antiox11112241.

Anti-NOX3 rabbit polyclonal, abcam, cat. no. ab81864. Validated by the company and the following publication: Liu, Z., et al. IFI6 depletion inhibits esophageal squamous cell carcinoma progression through reactive oxygen species accumulation via mitochondrial dysfunction and endoplasmic reticulum stress. J Exp Clin Cancer Res. 2020. 39(1):144. DOI: 10.1186/s13046-020-01646-3.

Anti-NOX4 rabbit polyclonal, abcam, cat. no. ab154244. Validated by the company and the following publication: Yin, Y. L. et al. Citronellal Attenuates Oxidative Stress-Induced Mitochondrial Damage through TRPM2/NHE1 Pathway and Effectively Inhibits Endothelial Dysfunction in Type 2 Diabetes Mellitus. 2022. Antioxidants (Basel). 11(11): 2214. DOI: 10.3390/antiox11112241.

Anti-γH2AX mouse monoclonal (JBW301), EMD Millipore, cat. no. 05-636-25UG. Validated by the company and the following publication: Gordon, et al. Plasma Progerin in Patients with Hutchinson-Gilford Progeria Syndrome: Immunoassay Development and Clinical Evaluation. Circulation. 2023. Mar 15. DOI: 10.1161/CIRCULATIONAHA.122.060002.

Anti-H2AX rabbit monoclonal, Cell Signaling, cat. no. 7631. Validated by the company and the following publication: Yuan, J., et al., Focus on histone variant H2AX: to be or not to be. FEBS Lett. 2010. 584(17): 3717-3724. DOI: 10.1016/j.febslet.2010.05.021.

Anti-p16 mouse monoclonal (G175-1239), BD Pharmingen, cat. no. 554079. Validated by the company and the following publication: Schlecht, N. F., et al. A comparison of clinically utilized human papillomavirus detection methods in head and neck cancer. Mod Pathol. 2011. 24(10): 1295-305. DOI: 10.1038/modpathol.2011.91.

Anti-β-actin mouse monoclonal, Proteintech, cat. no. 66009-1-Ig. Validated by the company and the following publication: Xu, Q., et al. The flavonoid procyanidin C1 has senotherapeutic activity and increases lifespan in mice. Nat Metab. 2021. 3(12): 1706-1726. DOI: 10.1038/s42255-021-00491-8.

Anti-GAPDH rabbit monoclonal, Abways, cat. no. AB0037. Validated by the company and the following publication: Xu, Q., et al. The flavonoid procyanidin C1 has senotherapeutic activity and increases lifespan in mice. Nat Metab. 2021. 3(12):1706-1726. DOI: 10.1038/s42255-021-00491-8.

Goat polyclonal anti-rabbit IgG H&L (HRP) (abcam cat. no. ab6721). Validated by the company and following publication:
Tian Y, Zhong L, Gao S, et al. LncRNA LINC00974 downregulates miR-122 to upregulate RhoA in oral squamous cell carcinoma. Cancer Biother Radiopharm. 2021 Feb;36(1):18-22. doi: 10.1089/cbr.2019.2907.

Goat polyclonal anti-mouse IgG H&L (HRP) (abcam cat. no. ab6789). Validated by the company and following publication:
Jin K, Wen Z, Wu B, et al. NOTCH-induced rerouting of endosomal trafficking disables regulatory T cells in vasculitis. J Clin Invest. 2021 Jan 4;131(1):e136042. doi: 10.1172/JCI136042.

Goat polyclonal to rabbit (or mouse) IgG Alexa Fluor 488 or 594-conjugated secondary, abcam cat. no. ab150077, ab150080, ab150113, ab150116. Validated by the company and the following publication: Boyi Zhang, Qilai Long, Shanshan Wu, et al. KDM4 orchestrates epigenomic remodeling of senescent cells and potentiates the senescence-associated secretory phenotype. Nat Aging. 2021 May;1(5):454-472. doi: 10.1038/s43587-021-00063-1.

Eukaryotic cell lines

Policy information about cell lines and Sex and Gender in Research

Cell line source(s)	Primary normal human prostate stromal cell line PSC27 (originally isolated from a male patient) and primary normal human breast stromal cell line HBF1203 (originally isolated from a female patient) were kind gifts (not commercialized) of Dr. Peter Nelson (FHCRC) and cultured in PSCC media as described in the Methods section. Human fetal lung primary stromal lines WI38, IMR90, HFL1, foreskin stromal line BJ and human embryonic kidney line 293T were from ATCC and cultured with F-12K medium supplemented with 10% FBS. Prostate cancer epithelial cell lines PC3, DU145, LNCaP and VCaP (ATCC) and breast cancer epithelial cell line MDA-MB-231 (ATCC) were routinely cultured with RPMI 1640 (10% FBS). Prostate cancer epithelial line M12 was a kind gift from Dr. Stephen Plymate (University of Washington), which was originally derived from the benign line BPH1 but phenotypically neoplastic and metastatic.
Authentication	All human cell lines were authenticated by genomic DNA profiling assays (STR) performed by XP Biomed.
Mycoplasma contamination	All cell lines in this study were tested negative for mycoplasma contamination.
Commonly misidentified lines (See ICLAC register)	None of the cell lines used in this study is present in the database of commonly misidentified cell lines.

Animals and other research organisms

Policy information about studies involving animals; ARRIVE guidelines recommended for reporting animal research, and Sex and Gender in Research

Laboratory animals	Nod-obese diabetic and severe combined immunodeficiency (NOD-SCID) mice and wild type C57BL/6J mice (NanJing Model Animal Centry, China) of 6-8 weeks old were housed and maintained in accordance with animal guidelines of Shanghai Institute of Nutrition and Health. All experimental mice were used and housed (22-25 C, 30% humidity) under 12 h light/12 h dark cycle (6 am-6 pm) with a standard rodent chow diet (5LOD, PicoLab) and water provided ad libitum. All animals involved in prostate tumour-associated experiments were male mice, while those involved in breast tumour-associated assays were female mice. For physical function assessments and lifespan extension assays, both male and female animals were used (wildtype C57BL/6J mice), with the number per sex largely equivalent throughout the preclinical trials. Generally, the were no sex- and gender-based analyses performed, as the major findings and overall conclusions are applicable to bother sexes and/or genders.
Wild animals	No wild animals were employed in this study.
Reporting on sex	Experimental findings in this study apply to both male and female animals, including those developing either prostate or breast tumours, as well as those involved in physical function appraisal and lifespan extension assays. As both genders were used for in vivo assays or preclinical trials, relevant experimental procedures and research conclusions are applicable to both sexes.
Field-collected samples	This study did not involve field-collected samples.
Ethics oversight	All animal experiments were conducted in compliance with NIH Guide for the Care and Use of Laboratory Animals (National Academies Press, 2011) and the ARRIVE guidelines, and were approved by the Institutional Animal Care and Use Committee (IACUC) of Shanghai Institute of Nutrition and Health, Chinese Academy of Sciences (protocol no. SINH-2022-SY-1).

Note that full information on the approval of the study protocol must also be provided in the manuscript.

Clinical data

Policy information about clinical studies

All manuscripts should comply with the ICMJE guidelines for publication of clinical research and a completed CONSORT checklist must be included with all submissions.

Clinical trial registration	Chemotherapeutic administration involving genotoxic agents was performed for primary prostate cancer patients (Clinical trial no. NCT03258320) and infiltrating ductal BCa patients (NCT02897700), by following the CONSORT 2010 Statement (updated guidelines for reporting parallel group randomized trials).
Study protocol	Data regarding tumour size, histologic type, tumour penetration, lymph node metastasis, and TNM stage were obtained from the pathologic records. Before chemotherapy, tumours were acquired from these patients as 'Pre' samples (an 'Untreated' cohort). After chemotherapy, remaining tumours in patients were acquired as 'Post' samples (a 'Chemo-treated' cohort, with most tumours collected within 1-6 months after treatment). For some cases, the 'Pre' and 'Post' tumour biopsies from the same individual patient were both accessible, and these samples were subject to further evaluation. Tumours were processed as FFPE biospecimens and sectioned for histological assessment, with alternatively prepared OCT-frozen chunks processed via lase capture microdissection (LCM) for gene expression analysis. Specifically, stromal compartments associated with glands and adjacent to cancer epithelium were separately isolated from tumour biopsies before and after chemotherapy using an Arcturus (Veritas Microdissection) laser capture microscope following previously defined criteria. The immunoreactive scoring (IRS) gives a range of 1–4 qualitative scores according to staining intensity per tissue sample. Categories for the IRS include 0-1 (negative), 1-2 (weak), 2-3 (moderate), 3-4 (strong). The diagnosis of prostate cancer tissues was confirmed based on histological evaluation by independent pathologists. Informed consent was obtained from all subjects and the experiments conformed to the principles defined in the WMA Declaration of Helsinki and the Department of Health and Human Services Belmont Report. No sex and/or gender was considered in the overall study design, nor sex and/or gender of participants determined based on self-report or assigned, as clinical investigations and principal conclusions in the total framework of this study are applicable to bother sexes and/or genders.

Detailed protocol can be referred to at https://www.clinicaltrials.gov/ct2/show/NCT03258320?cond=03258320&draw=2&rank=1 and https://www.clinicaltrials.gov/ct2/show/NCT02897700?cond=NCT02897700&draw=2&rank=1 (registered by the corresponding author and colleagues), or recent publications (Zhang et al. 2018. Nat Commun; Chen et al. 2018. Nat Commun; Xu et al. 2019. Aging Cell; Han et al. 2020. Cancer Res; Zhang et al. 2021. Nat Aging; Wang et al. 2022. Oncogene).

Data collection

Investigators performed a Phase I, single agent exploration, multicenter clinical trial to establish the treatment efficacy of several chemotherapeutic agents in patients with high risk localized prostate or breast cancer who have developed the primary cancer in prostate or breast, respectively. Up to 5 cohorts (PCa) or 6 cohorts (BCa) have been enrolled to determine the effectiveness and safety of single therapeutic strategy. Besides the five-year disease-free survival, overall survival and five-year metastasis-free survival post treatment, investigators also take into account the anticancer agent-induced tumour stroma damage extent, which may provide further evidence to support the treatment efficacy and assess the potential influence of a damaged tumour microenvironment (harboring a large number of senescent cells) on disease progression or regression in clinical settings.
Clinical data were mainly collected at the following sites: Qilu Hospital of Shandong University, Zhongshan Hospital of Fudan University for PCa patients; Ganzhou City People's Hospital, China-Japan Union Hospital of Jilin University, and Shanghai 10th People's Hospital of Tongji University School of Medicine. All these sites are located in China.

Outcomes

Primary Outcome Measures: 5 years disease-free survival. There is no disease-associated progression during the 5 years post treatment (chemotherapy plus surgery).
Secondary Outcome Measures : 5 years overall survival and metastasis-free survival. The overall survival status and distant metastasis-free survival status during the 5 years posttreatment (chemotherapy plus surgery) are evaluated.
Both primary and secondary outcomes were pre-defined according to former similar studies that involve chemotherapeutic administration of genotoxic agents and surgery performed for primary cancer patients , with relevant measures assessed by clinical follow-up of individual patients in posttreatment stage.
