# Supplementary figures and images for: PDK4-dependent hypercatabolism and lactate production of senescent cells promotes cancer malignancy
Source: Nat Metab. 2023 Oct 30;5(11):1887–910. doi: 10.1038/s42255-023-00912-w (PMC10663165; doi:10.1038/s42255-023-00912-w)

Source data Fig. 1c

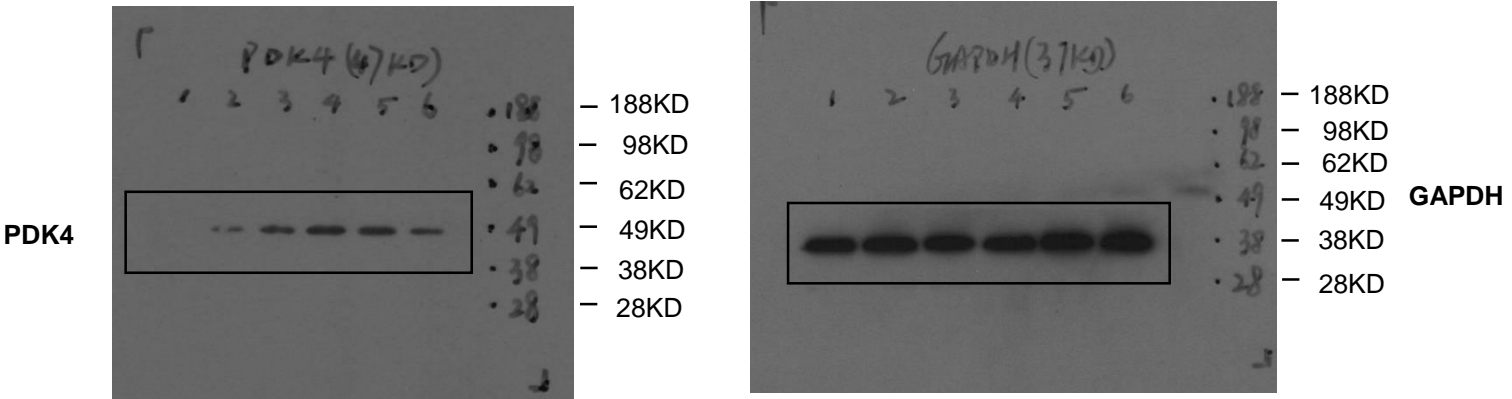

Source data Fig. 1g

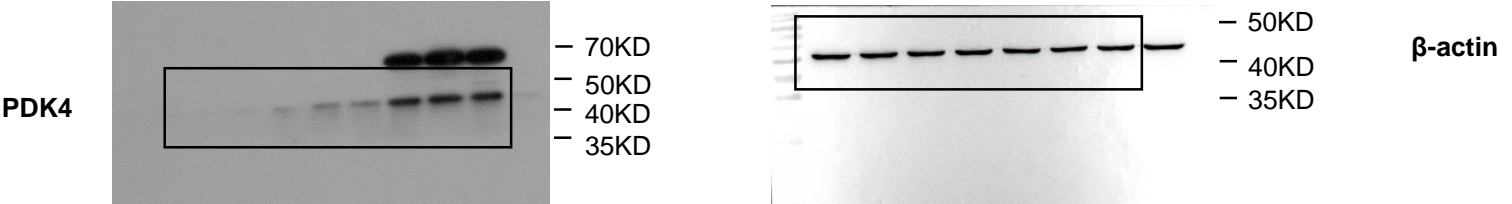

Source data Fig. 1i

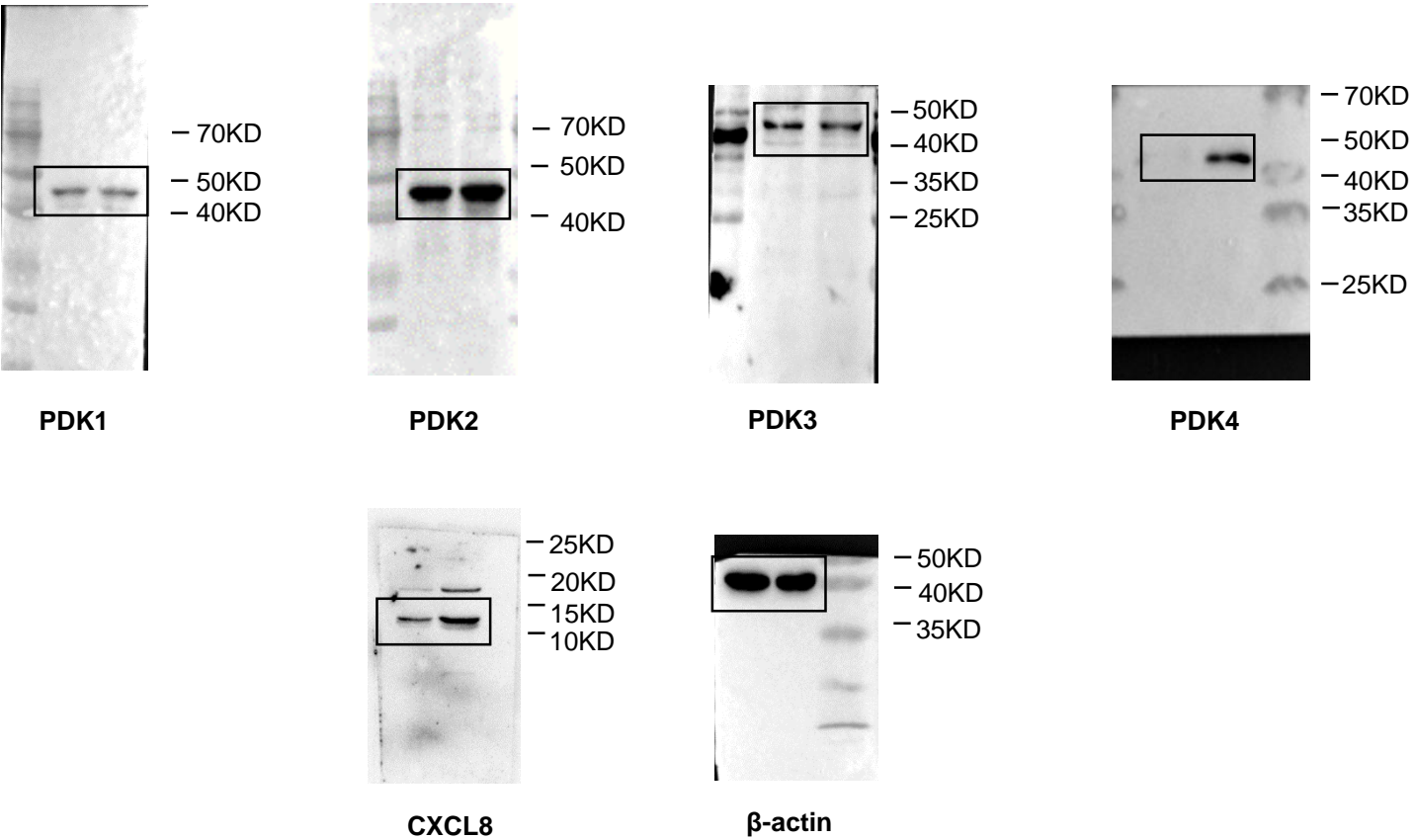

Source data Fig. 1j

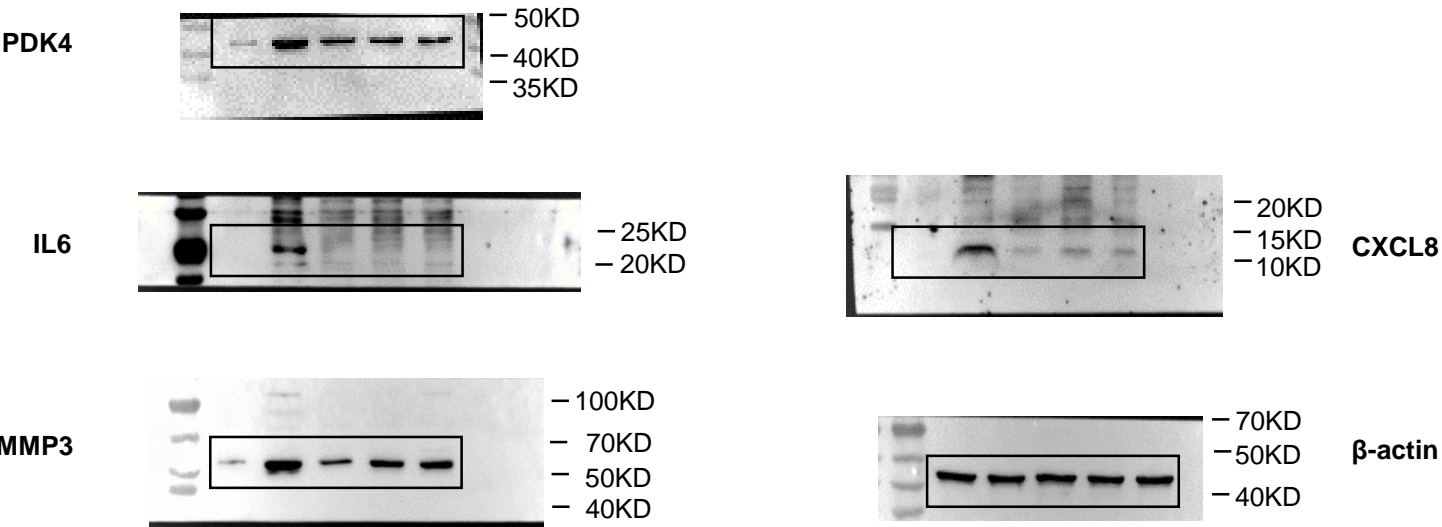

Supplement: Supplementary file 4 — Unprocessed western blots. [file 42255_2023_912_MOESM4_ESM.pdf]

Source data Fig. 7c

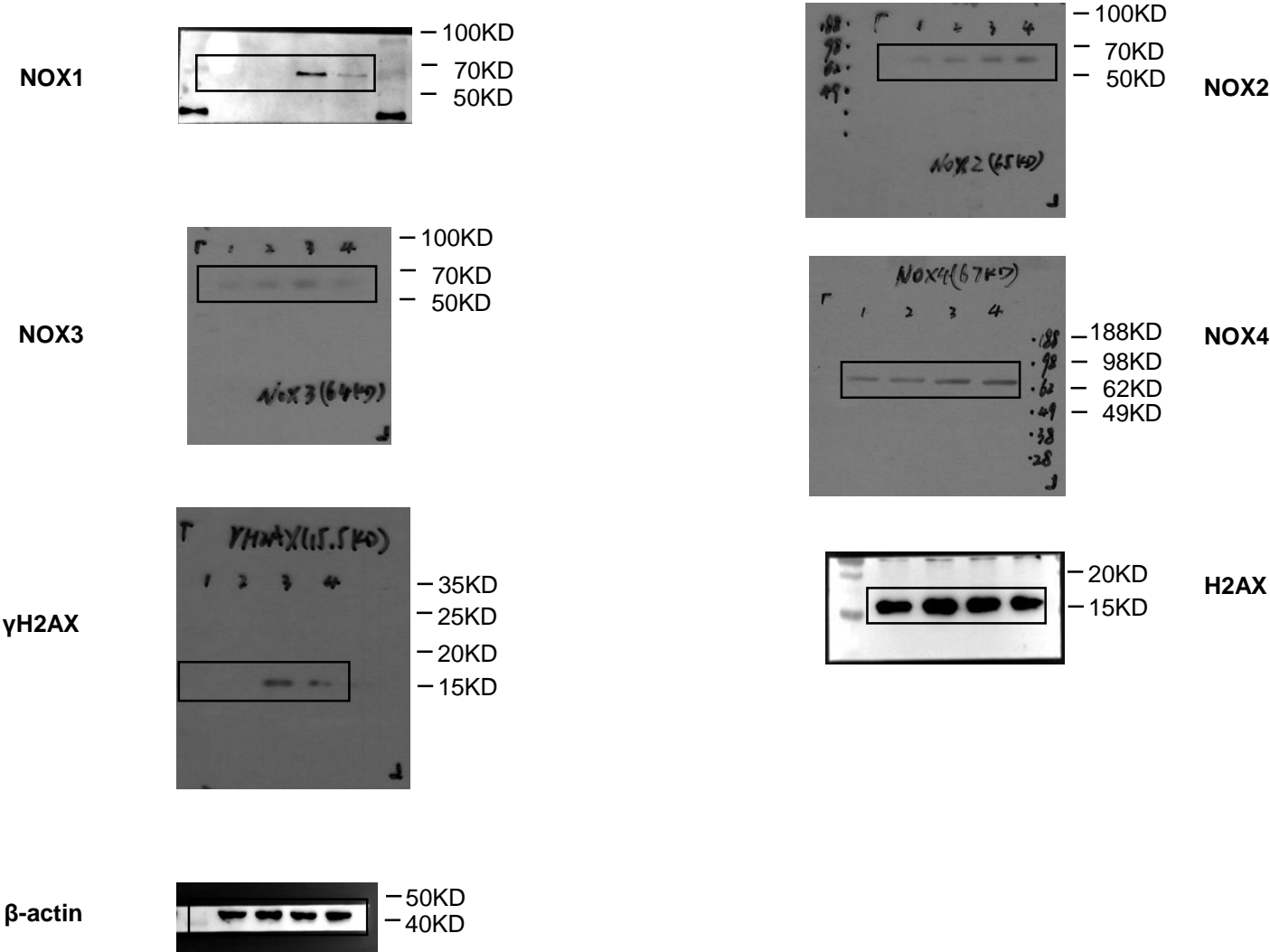

Source data Fig. 7g

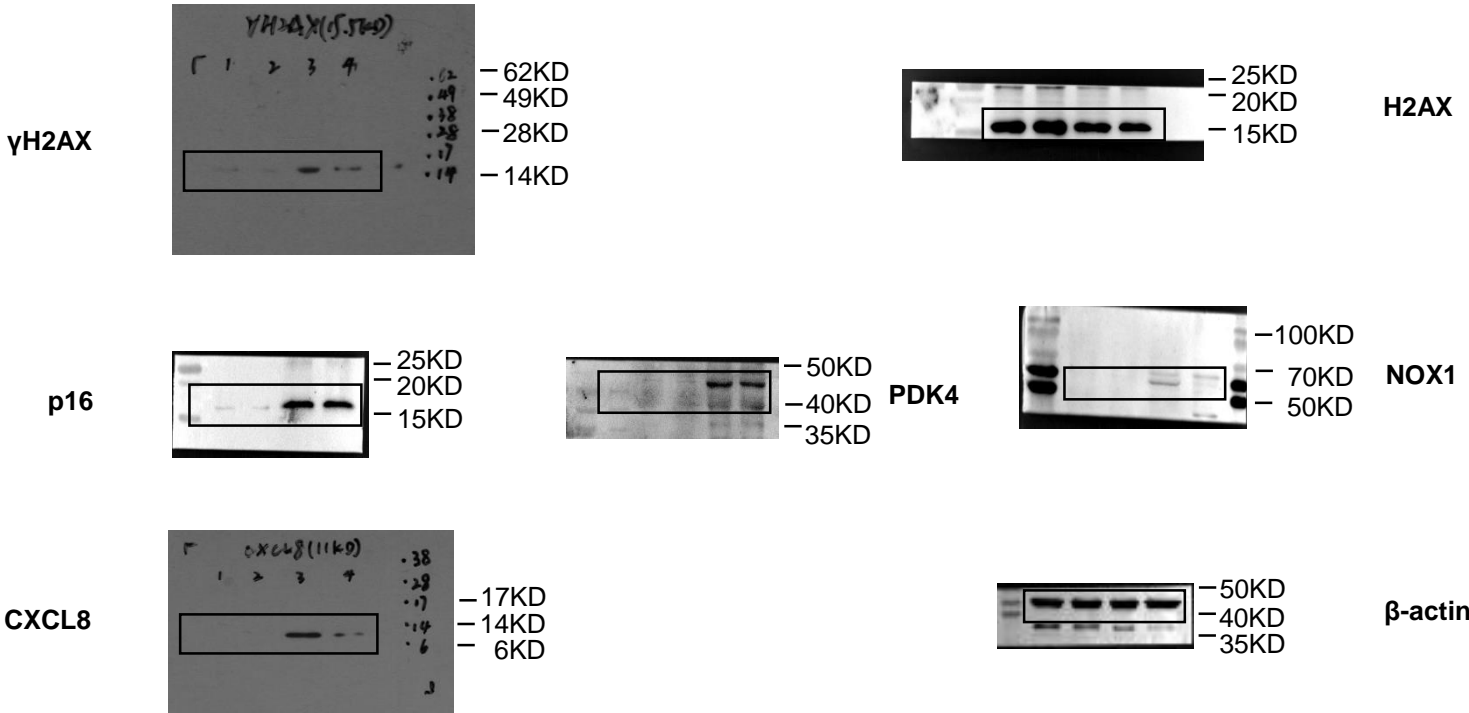

Supplement: Supplementary file 11 — Unprocessed western blots. [file 42255_2023_912_MOESM11_ESM.pdf]

Source data Extended Data Fig. 1e

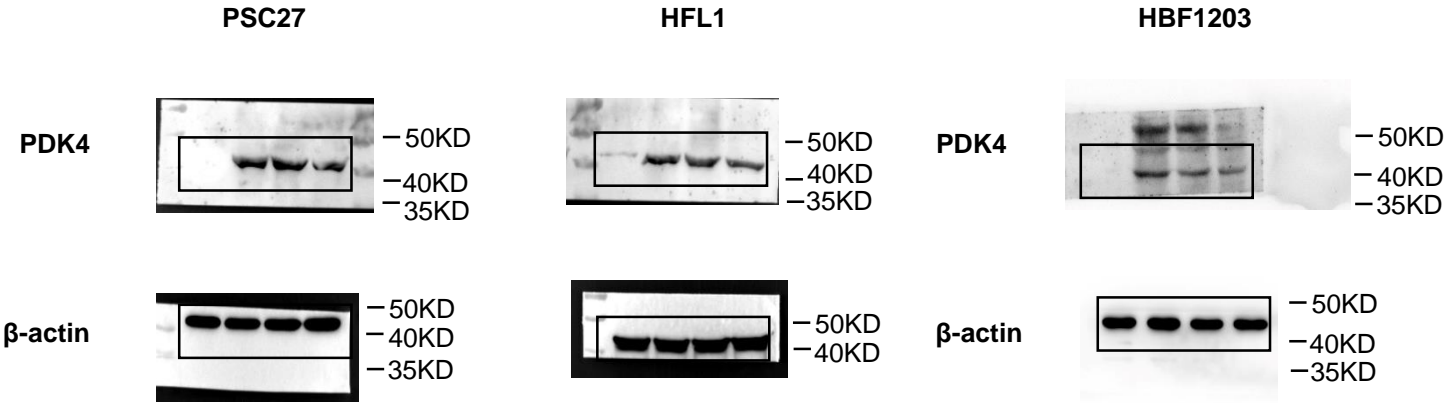

Supplement: Supplementary file 14 — Unprocessed western blots. [file 42255_2023_912_MOESM14_ESM.pdf]

Source data Extended Data Fig. 3o

PDK4

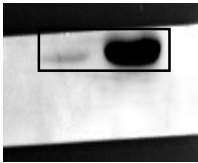

— 50KD  
— 40KD  
— 35KD

IL6

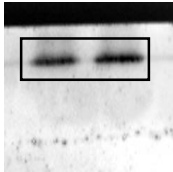

— 35KD  
— 25KD  
— 10KD

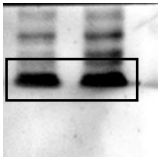

— 35KD  
— 25KD  
— 10KD

CXCL8

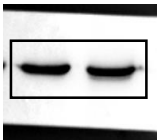

— 50KD  
— 40KD  
— 35KD

$\beta$ -actin

Supplement: Supplementary file 17 — Unprocessed western blots. [file 42255_2023_912_MOESM17_ESM.pdf]

Source data Extended Data Fig. 4o

PDK4 (LE)

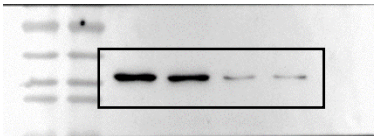

— 50KD  
— 40KD  
— 35KD

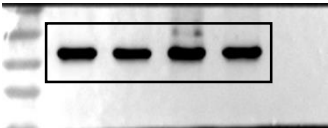

— 50KD  
— 40KD  
— 35KD

$\beta$ -actin

Supplement: Supplementary file 19 — Unprocessed western blots. [file 42255_2023_912_MOESM19_ESM.pdf]

Source data Extended Data Fig. 6d

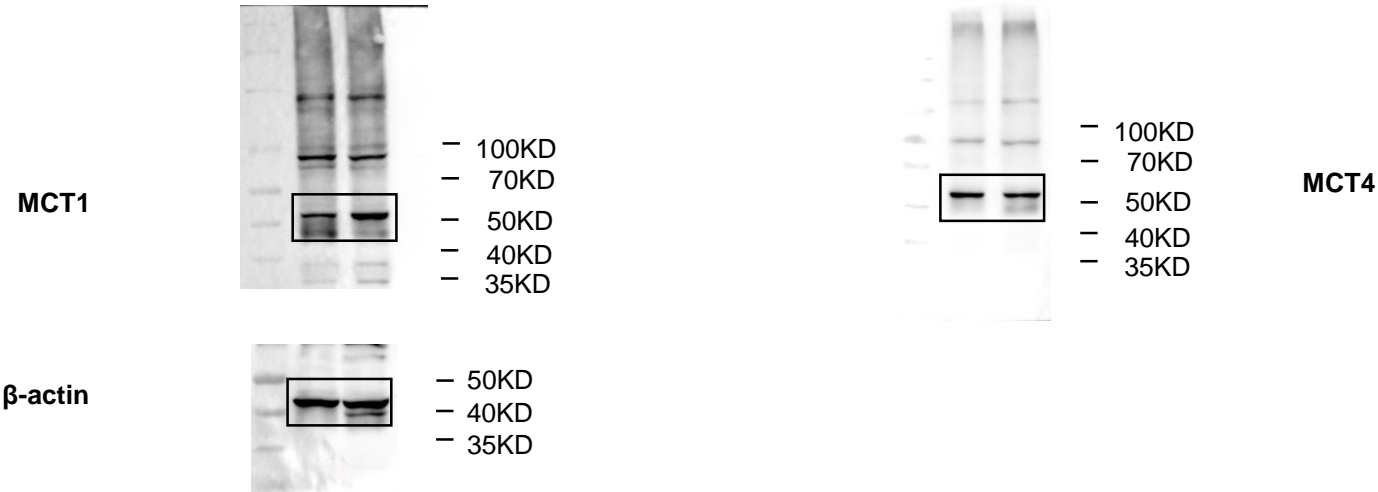

Source data Extended Data Fig. 6e

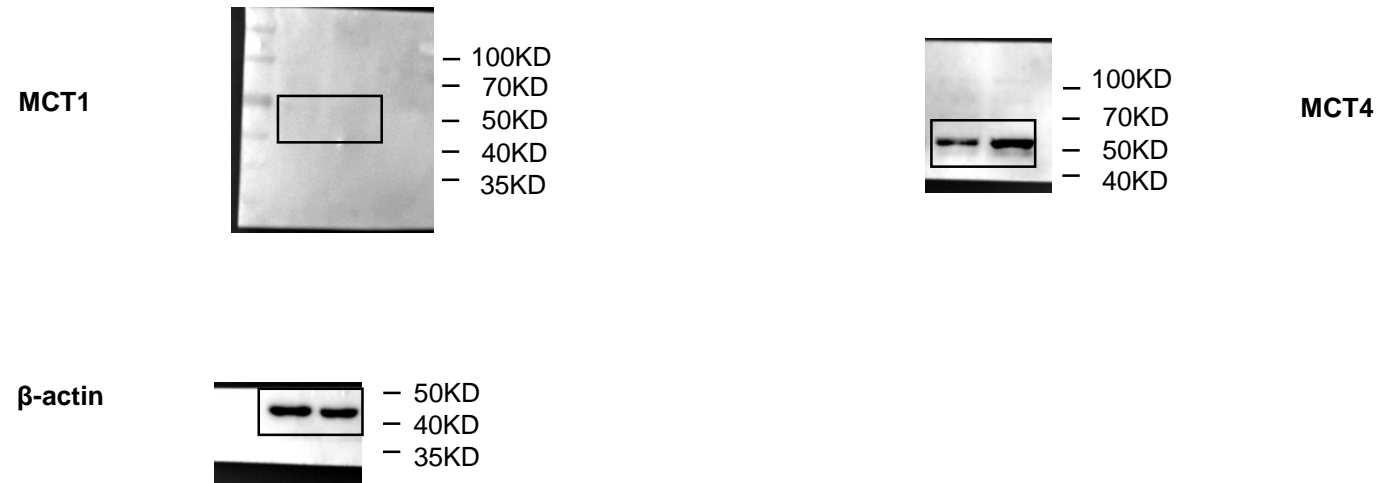

Supplement: Supplementary file 22 — Unprocessed western blots. [file 42255_2023_912_MOESM22_ESM.pdf]

Source data Extended Data Fig. 9a

NOX1

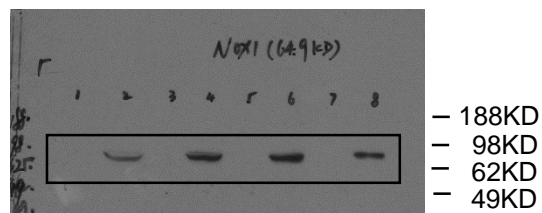

GAPDH

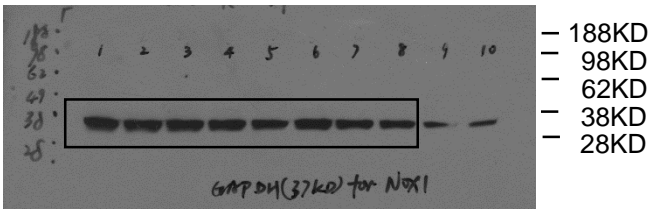

Supplement: Supplementary file 26 — Unprocessed western blots. [file 42255_2023_912_MOESM26_ESM.pdf]
