# Supplementary material for: Author Correction: PDK4-dependent hypercatabolism and lactate production of senescent cells promotes cancer malignancy
Source: Nat Metab. 2024 May 1;6(5):980. doi: 10.1038/s42255-024-01054-3 (PMC11132980; doi:10.1038/s42255-024-01054-3)

Author Correction: PDK4-dependent hypercatabolism and lactate production of senescent cells promotes cancer malignancy

In the format provided by the authors and unedited

Figure 4d

Before correction

Transcript	Fold change (upregulation)			
	PC12	DU145	M12	Average
HTR2B	20.35	31.41	14.40	22.05
AC069257.3	11.74	16.70	27.05	18.50
GRIK1	8.62	31.30	9.13	16.35
AL132656.3	8.43	22.61	8.96	13.34
FGD5	19.61	8.35	5.48	11.15
AC026316.4	8.25	11.41	12.41	10.69
LINC02617	7.17	14.12	6.03	9.11

After correction

Transcript	Fold change (upregulation)			
	PC3	DU145	M12	Average
HTR2B	20.35	31.41	14.40	22.05
AC069257.3	11.74	16.70	27.05	18.50
GRIK1	8.62	31.30	9.13	16.35
AL132656.3	8.43	22.61	8.96	13.34
FGD5	19.61	8.35	5.48	11.15
AC026316.4	8.25	11.41	12.41	10.69
LINC02617	7.17	14.12	6.03	9.11

Figure 8g

Before correction

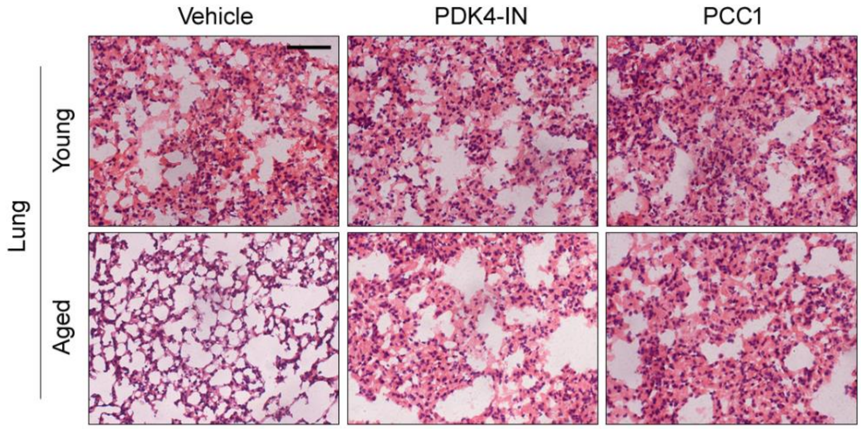

After correction

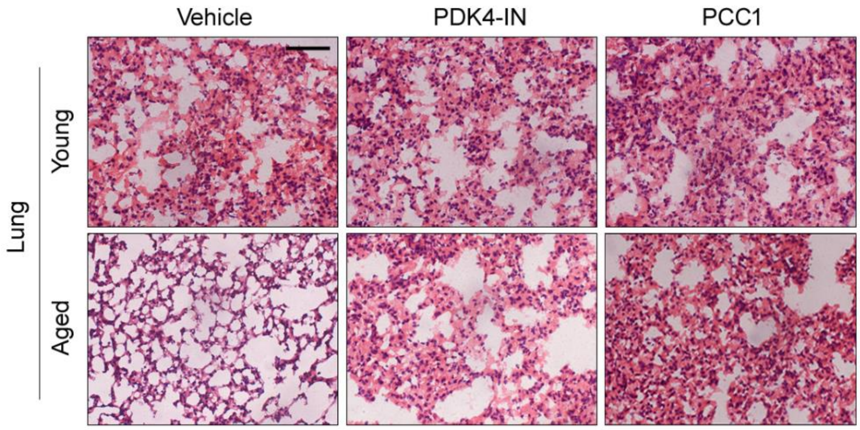

Extended Data Fig. 1b

Before correction

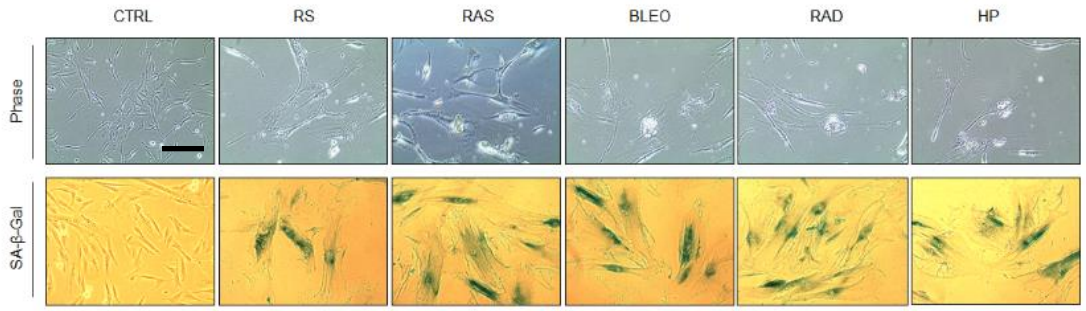

After correction

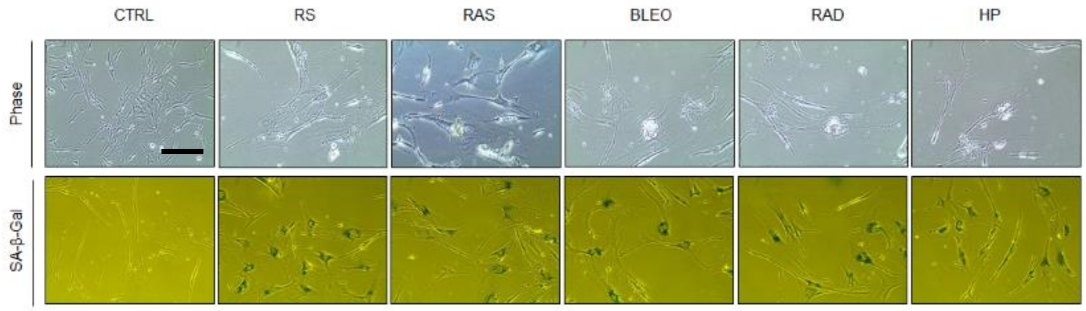

Supplement: Supplementary file 1 — Original and revised Fig. 4d, 8g and Extended Data Fig. 1b [file 42255_2024_1054_MOESM1_ESM.pdf]
